# MicroRNA miR-1275 coordinately regulates AEA/LPA signals via targeting FAAH in lipid metabolism reprogramming of gastric cancer

**DOI:** 10.1038/s41419-023-05584-8

**Published:** 2023-01-26

**Authors:** Qian Yang, Shan Kong, Jiajia Yu, Yanhua Xu, Mei Tao, Shuo Ma, Chenxue Tang, Xianjuan Shen, Zhiyuan Tang, Shaoqing Ju

**Affiliations:** 1 Department of Laboratory Medicine, Affiliated Hospital of Nantong University, Nantong, 226001 Jiangsu China; 2 Centre of Clinical Laboratory, First Affiliated Hospital of Soochow University, Suzhou, 215006 Jiangsu China; 3 Department of Laboratory Medicine, Jiangsu Province Official Hospital, Nanjing, 210009 Jiangsu China; 4 Research Centre of Clinical Medicine, Affiliated Hospital of Nantong University, Nantong, 216001 Jiangsu China; 5 Department of Pharmacy, Affiliated Hospital of Nantong University, Nantong, 226001 Jiangsu China

**Keywords:** Cancer metabolism, Oncogenesis, miRNAs

## Abstract

Glycerophospholipid signal and fatty acid metabolism are closely related to the occurrence and progression of tumours, and metabolic reprogramming caused by hydrolytic enzymes plays an important role in gastric cancer (GC). Here, we performed whole transcriptome sequencing and combined qRT-PCR to screen out the significantly high expression of fatty acid amide hydrolase (FAAH) in GC tissues, which was further verified in both TCGA and Oncomine databases. Functional tests confirmed that FAAH played an oncogene role in GC, and silencing FAAH could delay tumour growth, inhibit tumour metastasis, and promote cell apoptosis both in vitro and in vivo. FAAH-mediated lipid metabolism reprogramming through coordinated regulation of arachidonoyl ethanolamide (AEA)/lysophosphatidic acid (LPA) signalling and activated the cyclooxygenase-2 (COX-2)/prostaglandin E2 (PGE2) axis to promote GC progression. Luciferase reporter assay and immunofluorescence-fluorescence in situ hybridization (IF-FISH) were applied to validate the interactions of miR-1275/FAAH. Overexpression and knockdown of miR-1275 in vitro could indirectly modulate the above lipid signalling by targeting FAAH, thereby affecting GC progression. Our study indicates that deregulated FAAH is a key lipid signal and the miR-1275/FAAH/AEA/LPA axis can serve as a diagnostic biomarker for GC or as a target for therapy development.

## Introduction

Gastric cancer (GC) is one of the most common malignant tumours of the digestive tract, and it ranks sixth in incidence and third in mortality worldwide [1]. Lipolysis and fat production are disrupted in the tumour microenvironment, and free fatty acids (FFAs) derived from abnormally increased lipolysis and other small molecule metabolites encourage the occurrence and development of such tumours [2]. Therefore, it is important to investigate the molecular biological mechanisms of abnormally regulated lipid metabolism enzymes and related lipid signalling pathways during the occurrence and development of GC, to facilitate the development of targeted drugs for GC.

Lipids are one of the main components of cells, which play an essential role in the pathogenesis of inflammatory, autoimmune, malignant, and neurodegenerative diseases. Lipid mediators and hydrolytic enzymes involved in lipid metabolism remodelling are closely related to the development and treatment of GC. Yao et al. [3] found that fatty acid 2-hydroxylase plays a vital role in regulating Hh signal transduction and GC growth, and their results showed that the hydroxylated fatty acids catalysed by fatty acid 2-hydroxylase have the potential to be non-toxic broad-spectrum anti-cancer drugs. Ortega et al. [4] proposed that arachidonoyl ethanolamide (AEA) analogues could induce morphological changes, decreased viability, and increased GC cell apoptosis in a concentration-dependent manner. In addition, lysophosphatidic acid (LPA) was reported to induce the migration and invasion of GC cells via the Notch signalling pathway [5]. Fatty acids were found to cause high levels of O-glycosylation to promote the transcription of CD36 by activating the NF-κB pathway, thereby facilitating the metastasis of GC cells [6]. Prostaglandins, including prostaglandin E2 (PGE2), are catalysed by arachidonic acid (AA) through cyclooxygenase-2 (COX-2), and ω-6 polyunsaturated fatty acids, including AA, could be combined with PGE2 to enhance angiogenesis by promoting the proliferation and migration of human umbilical vein endothelial cells. Moreover, ω-3 polyunsaturated fatty acids inhibit angiogenesis through the intermediate metabolite PGE3 [7].

Fatty acid amide hydrolase (FAAH) is a membrane hydrolase with a complete N-terminal transmembrane domain, and it participates in the regulation of the endocannabinoid system and decomposes biologically active lipids, including AEA and 2-arachidonoylglycerol [8]. The endocannabinoid system is a pleiotropic signalling system that is activated in a time- and tissue-specific manner during pathological conditions, such as in tumours. Abnormally expressed FAAH in tumours could promote the hydrolysis of AEA, weaken the anti-tumour effect mediated by AEA, and enhance the hyperalgesia of patients with tumour. However, AA catalysed by FAAH was found to be highly expressed in the tissues of patients with colorectal cancer metastasis [9]. Moreover, accumulation of AA enhances the migration and invasion capabilities of breast cancer cells [10]. Therefore, inhibiting the expression of FAAH retained the level of endocannabinoid AEA and reduced the accumulation of FFA, which represents a potential method of treating cancer.

In this study, we detected the expression of FAAH in GC tissues and clarified its effects on tumour progression. In addition, we evaluated the changes in downstream lipid signals after FAAH interference and searched for upstream miRNAs to explore the role of the miR-1275/FAAH signalling axis in the malignant progression of GC by regulating lipid metabolism reprogramming.

## Results

### Correlation between FAAH expression and clinicopathological parameters in GC

**Fig. 1:**
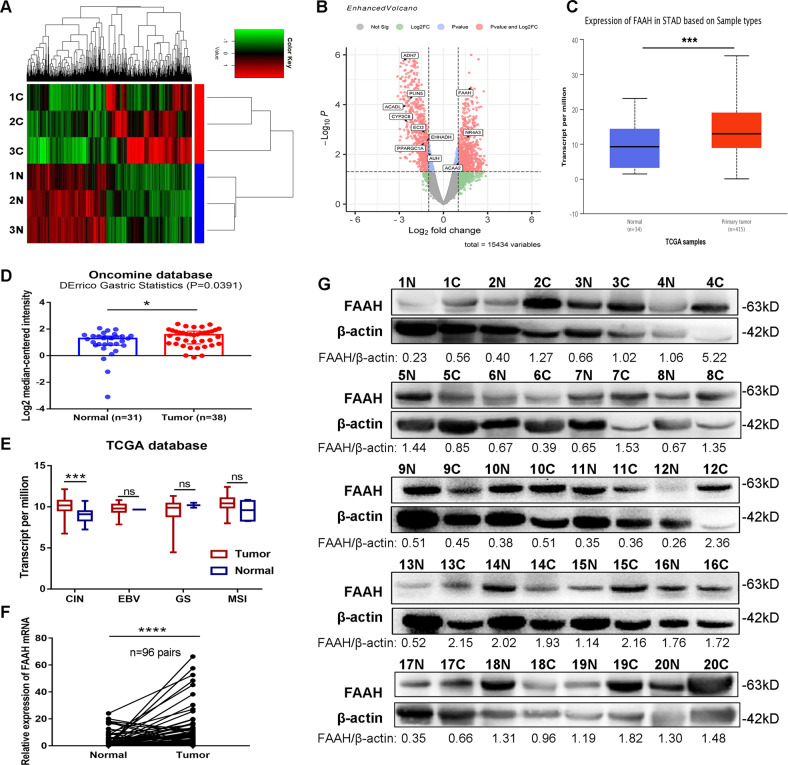
FAAH is highly expressed in GC tissues. **A** Clustered heatmap. Each row represents a tissue sample, and each column represents a coding gene. **B** Volcano plots. The red points on the right indicate the differentially upregulated genes with statistical significance while the red points on the left indicate the downregulated genes. Differential expression of FAAH in tissues from **C** TCGA database and **D** Oncomine database. **E** Differential expression analysis of FAAH in 4 GC subtypes (data from TCGA). **F** mRNA levels of FAAH in 96 pairs of GC tissues. **G** Protein levels of FAAH in 20 pairs of GC tissues. *p < 0.05, ***p < 0.001, ****p < 0.0001. N non-cancerous tissue, C cancerous tissue, CIN tumours with chromosomal instability, EBV tumours positive for Epstein-Barr virus, GS genetically stable tumours, MSI microsatellite unstable tumours.

We performed high-throughput sequencing in three pairs of fresh GC tissues to construct differential mRNA expression profiles in GC (Fig. 1A). The DESeq algorithm analysis revealed that 762 genes were upregulated. We used GO and KEGG enrichment analyses to screen 11 genes related to fatty acid metabolism/synthesis and lipid modification. FAAH showed the highest fold change among the upregulated genes; thus, we chose this for further research (Fig. 1B). We checked the expression of FAAH in the TCGA and Oncomine databases, and the findings showed that the expression trend was consistent with the sequencing results despite the limited number of sequencing samples (Fig. 1C, D). Based on integrated genomics analysis, TCGA divides GC into four subtypes. Through further analysis, we found that the expression of FAAH in tumours with chromosomal instability (CIN) was significantly different from that in normal tissues (Fig. 1E).

The FAAH gene sequence was easily amplified by its specific primers indicating high operability for clinical application (Supplementary Fig. S1a–c). We subsequently collected another 96 pairs of cancerous and adjacent non-cancerous tissues and detected FAAH expression by qRT-PCR; we found that FAAH was relatively upregulated in 77 GC tissues (Fig. 1F, *p* < 0.0001). In addition, we randomly selected 20 pairs of the above 96 tissues to verify the expression of the FAAH protein. Moreover, we discovered that FAAH was highly expressed in 70% (14/20) of GC tissues compared to adjacent tissues (Fig. 1G). Analysis of clinicopathological parameters showed that the high expression of FAAH was related to the patient’s sex, tumour size, invasion depth, WHO classification and lymph node metastasis, but was not related to Lauren classification, degree of differentiation and *H. pylori* status of patients (Table 1). We also examined the levels of FAAH in other common solid tumours, such as lung, breast, and colorectal cancer, and found that the expression of FAAH was specific in GC (Supplementary Fig. S1d). Furthermore, ROC analysis showed that FAAH had diagnostic power as a marker of GC, with an AUC of 0.7059 (Supplementary Fig. S1e).

**Table 1:** The relationship between FAAH expression and clinicopathological characteristics of GC patients.

Characteristics	No.	FAAH	*U* value	*p* value
		Median (IQR 25–75)		
Gender			580.0	0.016*
Male	72	1.842 (0.513–8.246)		
Female	24	6.074 (2.111–11.44)		
Age (year)			746.0	0.426
≥60	73	2.836 (0.731–9.243)		
<60	23	2.096 (0.370–9.193)		
Tumour size (cm)			719.0	0.002**
≥5	58	4.486 (1.164–13.01)		
<5	41	0.918 (0.425–3.990)		
Invasion depth			507.5	<0.001***
Infiltration into Serous layer	62	4.104 (1.344–13.17)		
Without Infiltration into Serous layer	34	0.630 (0.333–3.560)		
WHO classification			/	/
Adenocarcinoma NOS	38	1.179 (0.576–4.041)		/
Adenocarcinoma SignetRing	9	9.193 (2.152–22.67)		0.143^a^
Adenocarcinoma Tubular	19	9.129 (4.161–27.02)		<0.001^b^***
Adenocarcinoma Papillary	6	10.54 (1.494–13.67)		0.836^c^
Adenocarcinoma Mucinous	13	0.810 (0.341–1.923)		0.998^d^
Other	11	2.921 (0.593–13.05)		0.340^e^
Lauren classification			/	/
Intestinal type carcinoma	41	5.712 (1.159–12.96)		0.091^f^
Diffuse gastric carcinoma	40	1.123 (0.479–8.050)		0.400^g^
Other	15	2.096 (0.881–4.161)		0.963^h^
Differentiation			829.5	0.165
Poorly	65	2.687 (0.830–11.16)		
Moderately	31	2.785 (0.241–8.838)		
Lymph node metastasis			618.0	0.002**
Yes	65	3.934 (0.982–11.99)		
No	31	0.850 (0.350–4.810)		
H. pylori status			769.5	0.108
Positive	67	3.565 (0.810–11.72)		
Negative	29	1.401 (0.579–6.799)		

Statistical analysis was carried out using Mann–Whitney *U* test. One-way ANOVA was used when multiple sample comparisons were involved, ^a^Adenocarcinoma SignetRing vs. Adenocarcinoma NOS, ^b^Adenocarcinoma Tubular vs. Adenocarcinoma NOS, ^c^Adenocarcinoma Papillary vs. Adenocarcinoma NOS, ^d^Adenocarcinoma Mucinous vs. Adenocarcinoma NOS, ^e^Other vs. Adenocarcinoma NOS, ^f^Intestinal type carcinoma vs. Diffuse gastric carcinoma, ^g^Intestinal type carcinoma vs. Other, ^h^Diffuse gastric carcinoma vs. Other.

*p < 0.05; **p < 0.01; ***p < 0.001. *IQR* interquartile range, *NOS* not otherwise specified.

### Interfering with FAAH suppresses the malignant phenotype of GC

**Fig. 2:**
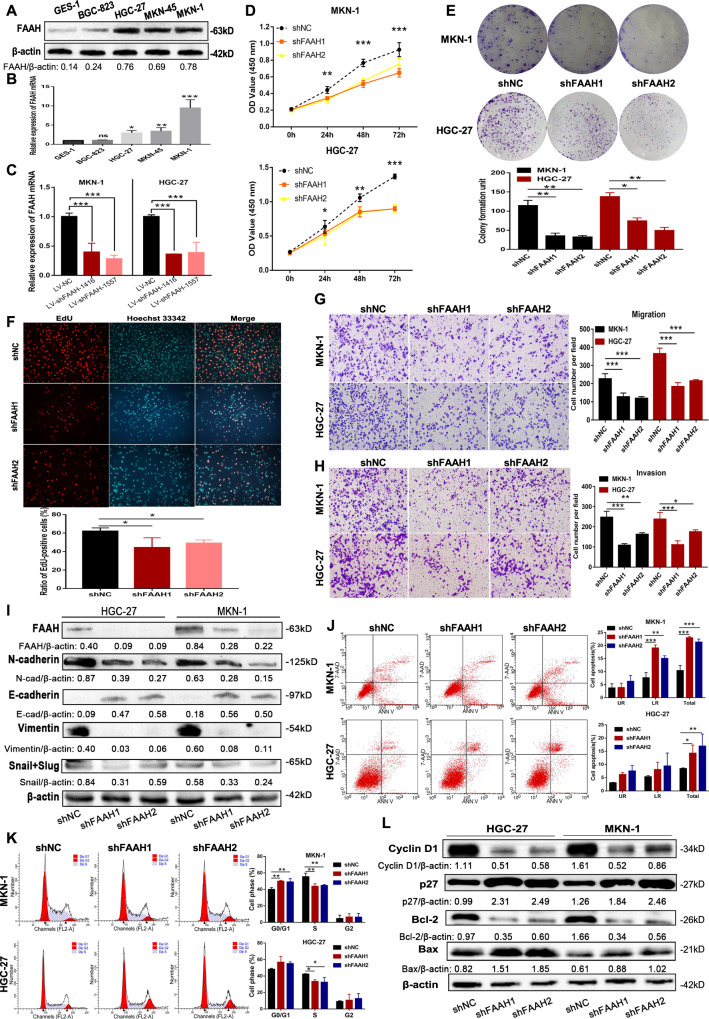
Interfering with FAAH inhibits the malignant phenotype of GC cells in vitro. Detection of FAAH expression in GC cell lines, and normal gastric epithelial cells by (**A**) western blotting and (**B**) qRT-PCR. **C** Knockdown efficiency of FAAH in lentiviral stably transfected cells. **D** CCK-8 was used to detect the proliferation of GC cells after interference with FAAH. **E** Cell colony formation assay. **F** Proportion of cells in the proliferation phase (labelled with red fluorescence) after interference with FAAH was detected by EdU. Transwell assay was used to evaluate the ability of (**G**) migration and (**H**) invasion after interference with FAAH. **I** Changes of EMT-related markers after FAAH knockdown. Flow cytometry was used to detect (**J**) proportion of apoptotic cells and (**K**) cycle distribution after interference with FAAH. **L** Changes of apoptosis and cycle-related proteins after FAAH interference. *p < 0.05, **p < 0.01, ***p < 0.001.

Analysis showed that FAAH was highly expressed in three GC cell lines (except BGC-823) compared to the GES-1 cells (Fig. 2A, B). Only two of the four interfering plasmids we constructed had good interfering functions (Supplementary Fig. S2a). The two plasmids were then packaged into lentiviral vectors to obtain vectors with relatively high knockdown efficiency, namely LV-shFAAH-1416 and LV-shFAAH-1557 (hereinafter referred to as shFAAH1 and shFAAH2, respectively, Fig. 2C, I). Overexpression of FAAH was also carried out in HGC-27 and BGC-823 cells (Supplementary Fig. S2b). Compared with the control group, the proliferation rate of GC cells was reduced after FAAH interference and the colony-forming units were significantly reduced (Fig. 2D, E). In addition, increased proliferation rate and clone-forming units were observed after FAAH overexpression (Supplementary Fig. S2c, d). The EdU assay also showed that the proportion of cells in the proliferation phase decreased after interference with FAAH, but increased after overexpression (Fig. 2F and Supplementary Fig. S2e). Moreover, silencing of FAAH weakened the cell migration and invasion ability, thereby inhibiting the epithelial-mesenchymal transition (EMT), which was manifested by increased level of E-cadherin and reduced level of N-cadherin, Snail (Slug), and Vimentin (Fig. 2G–I). And migration and invasion were strengthened after the overexpression of FAAH (Supplementary Fig. S2f, g). In addition, the apoptotic rate of GC cells increased and the proportion of S-phase cells decreased after FAAH interference (Fig. 2J, K), while the opposite effects were observed after FAAH overexpression (Supplementary Fig. S2h, i). Western blot analysis showed that the expression of Bcl-2 and Cyclin D1 decreased and the expression of Bax and p27 increased after interference with FAAH, indicating that apoptosis was promoted and the cell cycle was blocked (Fig. 2L). In addition, overexpression of FAAH showed the opposite trend (Supplementary Fig. S2j). Therefore, FAAH exhibited a significant cancer-promoting effect, and interference with FAAH in vitro inhibited the malignant phenotype of GC cells.

### FAAH interference inhibits tumour growth in vivo

**Fig. 3:**
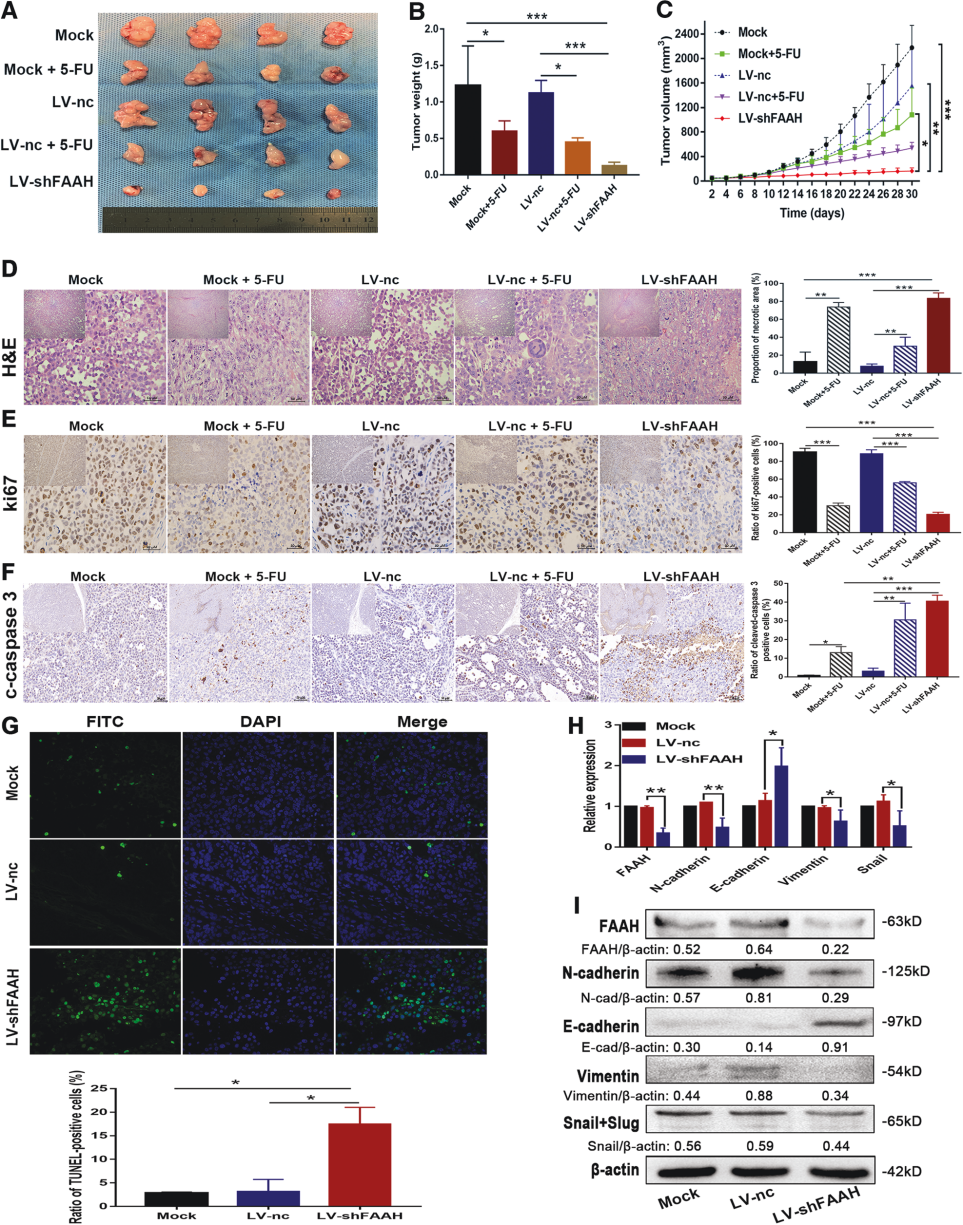
FAAH depression inhibits tumour growth and metastasis in vivo. **A** Tumour specimens photographed with a high-definition digital camera. **B** Weight of exfoliated tumours was measured by a tray balance. **C** Tumour growth curve was plotted by measuring the tumour volume every 2 days. **D** H&E staining of exfoliated tumour tissues (magnification: upper left corner, ×100; whole, ×400); IHC staining of tumour tissues with (**E**) anti-ki67 and (**F**) anti-cleaved caspase 3 antibody (magnification: upper left corner, ×100; whole, ×400). **G** Apoptotic cells of xenografts detected by TUNEL (magnification: ×400). Detection of EMT markers in tumour tissues by (**H**) qRT-PCR and (**I**) western blotting. *p < 0.05, **p < 0.01, ***p < 0.001. c-caspase 3 cleaved caspase 3.

We previously packaged two shRNA fragments into lentiviruses and obtained GC cell lines with stable FAAH knockdown through puromycin screening. Based on the results shown in Fig. 2C, we selected the shFAAH-1557 fragment with better knockdown efficiency for in vivo experiments. Untreated MKN-1 cells (mock), lentiviral negative control (LV-nc), and MKN-1 cells with stable knockdown of FAAH (LV-shFAAH) were injected into nude mice to construct tumour xenograft models, followed by 5-fluorouracil (5-FU) to act as the control group of chemotherapeutic drugs. The results showed that the LV-shFAAH group had small tumour size, light tumour weight, and slow growth rate compared to the control group (Fig. 3A–C). In addition, compared with the control group, the LV-shFAAH group had more necrotic areas, manifested as soma lysis and nuclear condensation (Fig. 3D). IHC staining showed that ki67 staining was attenuated in the LV-shFAAH group, while cleaved caspase-3 staining was enhanced, suggesting that FAAH knockdown reduced tumour proliferation and promoted apoptosis in vivo (Fig. 3E, F). TUNEL immunofluorescence also showed that the apoptosis of tumour cells increased after interference with FAAH (Fig. 3G). In addition, qRT-PCR and western blot analysis of tumour tissues showed that silencing of FAAH inhibited the EMT of GC cells, indicating that tumour metastasis was inhibited (Fig. 3H, I).

### miR-1275 as FAAH sponge in GC cells

**Fig. 4:**
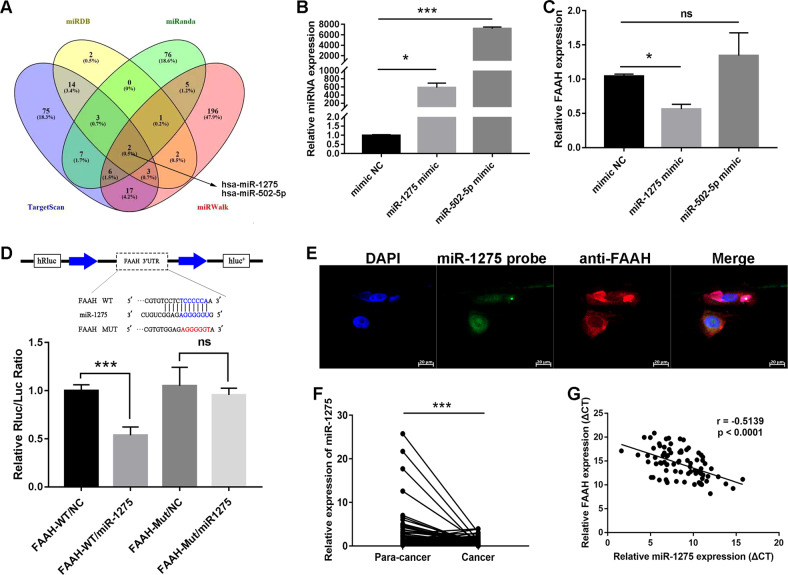
miR-1275 complementarily binds to the 3’-UTR of FAAH. **A** Combination of four public databases to screen miRNAs that could bind to FAAH. **B** Transfection efficiency of miR-1275 and miR-502-5p mimics in GC cells was detected by qRT-PCR. **C** Changes of FAAH expression after overexpression of miR-1275 and miR-502-5p. **D** Wild-type and mutant plasmids of FAAH 3’-UTR were constructed for dual-luciferase reporter gene assay. **E** IF-FISH assay was performed to visualise the subcellular localisation of FAAH (Red) and miR-1275 (Green) in MKN-1 cells. **F** Expression of miR-1275 in GC tissues (*n* = 80 pairs). **G** Spearman’s correlation analysis of miR-1275 and FAAH in GC tissues (*n* = 80 pairs). *p < 0.05, ***p < 0.001. WT wild-type, MUT mutant-type.

We searched four public databases (miRDB, miRanda, TargetScan, and miRWalk) for miRNAs that might have binding sites to the 3’-UTR of FAAH, respectively. Two miRNAs (miR-1275 and miR-502-5p) were obtained by taking the intersection of all possible miRNAs (Fig. 4A). Mimics of miR-1275 and miR-502-5p were then transfected into MKN-1 cells, and we found that only miR-1275 could downregulate the expression level of FAAH (Fig. 4B, C). The dual-luciferase reporter gene assay confirmed that miR-1275 had a binding site with the 3’-UTR of FAAH (Fig. 4D). Moreover, the IF-FISH assay showed that FAAH and miR-1275 could be partially combined in the cytoplasm (Fig. 4E). Next, miR-1275 was initially observed to be expressed at low levels in four GC cell lines and 80 pairs of GC tissues (Fig. 4F and Supplementary Fig. S3a). Spearman correlation analysis disclosed a negative correlation between miR-1275 and FAAH in GC tissues (Fig. 4G).

Our previous results showed that the expression of FAAH was elevated in three GC cell lines relative to the normal controls, with the highest expression level in MKN-1 cells and a relatively low expression level in HGC-27 cells (Fig. 2A, B). Considering that miR-1275 is an upstream regulator of FAAH, to verify whether miR-1275 can reverse the effect of FAAH on cell phenotype, we inhibited miR-1275 in HGC-27 cells with a stable knockdown of FAAH, and a miR-1275 mimic was added to MKN-1 cells overexpressing FAAH (Supplementary Fig. S3b). As shown in Supplementary Fig. S3c, miR-1275 could indeed regulate the expression of FAAH. FAAH expression in MKN-1 cells overexpressing miR-1275 was decreased compared to that of the control group, while that of HGC-27 cells with downregulated miR-1275 was increased. Functional tests showed that overexpression of miR-1275 could slow down the proliferation rate, reduce clonal colonies formation and the proportion of proliferating cells, inhibit cell metastasis, and induce apoptosis and cycle arrest; while miR-1275 inhibitor exerted a promoting effect on the malignant phenotype of GC cells (Supplementary Fig. S3d–j).

### miR-1275 partially reverses the cancer-promoting effects of FAAH

**Fig. 5:**
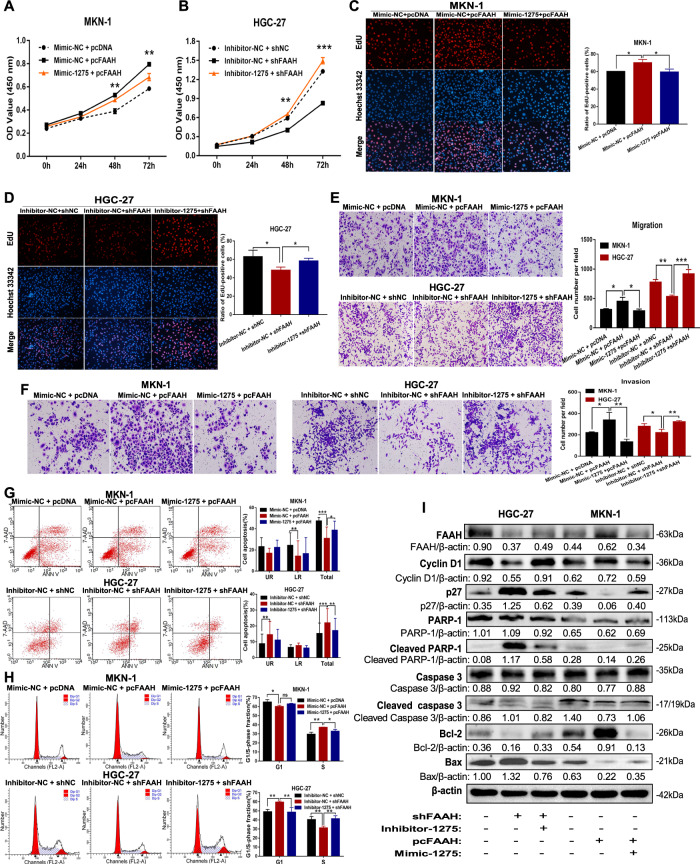
miR-1275 partially reverses the cancer-promoting effects of FAAH. **A**–**D** Proliferation of MKN-1 cells co-transfected with miR-1275 (inhibitor or mimic) and FAAH vectors (pcFAAH or shFAAH) was detected by the CCK-8 and EdU assay. The transwell assay was used to detect (**E**) migration and (**F**) invasion after co-transfection of miR-1275 and FAAH in MKN-1 and HGC-27 cells. Changes of (**G**) cell apoptosis and (**H**) cycle progression after co-treatment with miR-1275 and FAAH plasmids were measured by flow cytometry. **I** Detection of apoptosis and cycle-related proteins by western blotting. *p < 0.05, **p < 0.01, ***p < 0.001.

By co-transfecting the miR-1275 mimic and FAAH overexpression vector (pcFAAH) into MKN-1 cells, we found that the miR-1275 overexpression eliminated the proliferation-promoting effect of pcFAAH (Fig. 5A, C). Similarly, miR-1275 inhibitor reversed the inhibitory effect of the FAAH interference vector (shFAAH) on cell proliferation (Fig. 5B, D). In addition, the promoting effects on cell migration and invasion mediated by pcFAAH were also reversed by miR-1275 mimic, and miR-1275 inhibitor reversed shFAAH effects (Fig. 5E, F). When cells were treated with the miR-1275 mimic and pcFAAH, cell apoptosis decreased and the cell cycle slowed down, while the miR-1275 inhibitor restored shFAAH-mediated apoptosis and cycle arrest (Fig. 5G, H). This phenomenon was further verified by western blotting. MiR-1275 inhibitor could reverse the changes of apoptosis- and cycle-related proteins caused by FAAH interference, upregulate cyclinD1 and Bcl-2 protein levels, and downregulate the levels of p27, cleaved caspase 3, cleaved PARP-1, and Bax; the effects of FAAH overexpression on the above proteins could also be reversed by miR-1275 mimics (Fig. 5I).

### PF-3845 partially reverses the oncogenic effects of FAAH by inhibiting its activity

Considering that the essence of FAAH is a hydrolase, we found that the activity of FAAH was significantly enhanced in three GC cell lines (excluding BGC-823, Supplementary Fig. S4a). PF-3845 is a potent, selective, irreversible, and orally active inhibitor that inhibits FAAH activity by carbamylating FAAH’s serine nucleophile. First, the IC_50_ of MKN-1, HGC-27, and BGC-823 cells were measured by CCK-8, respectively (Supplementary Fig. S4b). We then treated cells with different concentrations of PF-3845 according to the measured IC_50_, and found that PF-3845 inhibited the proliferation of GC cells in a time- and concentration-dependent manner. Specifically, the longer the administration time, the lower the PF-3845 concentration required to achieve 50% proliferation inhibition (Supplementary Fig. S4c). In addition, the effects of PF-3845 on the metastasis, apoptosis, and cell cycle of MKN-1 cells were also concentration-dependent (Supplementary Fig. S4d–f).

To clarify whether this oncogenic effects of FAAH is due to its increased enzyme activity, different concentrations of PF-3845 were used in BGC-823 and HGC-27 cells that stably overexpress FAAH. The results showed that both 1/2 IC_50_ and IC_50_ concentrations of PF-3845 eliminated the proliferation effect of FAAH overexpression (Supplementary Fig. S4g), and the combined use of shFAAH and PF-3845 resulted in a higher inhibition rate (Supplementary Fig. S4h). In addition, PF-3845 reduced the strengthened ability of cell migration and invasion caused by FAAH overexpression (Supplementary Fig. S4i). Flow cytometry also suggested that PF-3845 reversed the effects of pcFAAH on apoptosis and cell cycle progression (Supplementary Fig. S4j, k). These results suggest that FAAH mediates cancer-promoting effects mainly due to its increased activity, and its activity inhibitor can weaken the malignant phenotype of GC.

### miR-1275 targets FAAH to coordinate AEA/LPA signalling in GC

**Fig. 6:**
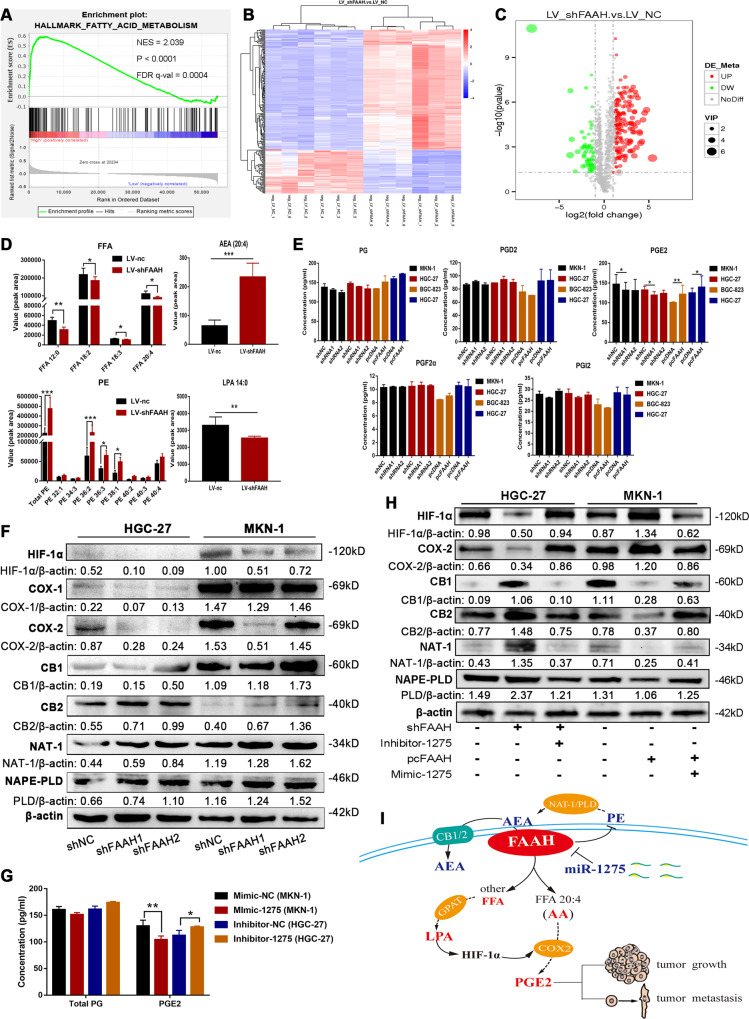
miR-1275 targets FAAH to coordinate AEA/LPA signalling in GC. **A** GSEA analysis of FAAH and fatty acid metabolism (data from the gastric adenocarcinoma database in the TCGA). **B** Heatmap of differential lipid metabolites in negative ion mode after interference with FAAH. **C** The Volcano plots. **D** Bar graph of differential lipid metabolites after interference with FAAH. **E** The content of total PG, PGD2, PGE2, PGF2α, and PGI2 in the cell supernatant was determined by ELISA after FAAH interference and overexpression. **F** Detection of changes in lipid signal involved in AEA/LPA pathway after interference with FAAH. **G** Content of total PG and PGE2 in the cell supernatant after treatment with miR-1275 mimic or inhibitor. **H** Changes of lipid signal in AEA/LPA pathway after co-transfection of miR-1275 and FAAH vectors. **I** Schematic diagram. *p < 0.05, **p < 0.01, ***p < 0.001. NES normalised enrichment score, FDR false discovery rate.

The GSEA analysis showed that FAAH significantly affected fatty acid metabolism (Fig. 6A). We next explored whether a series of lipid mediator changes involved in the process of FAAH-hydrolysing substrates had a certain impact on its biological behaviour. We performed lipidomics detection using MKN-1 cells with stable FAAH knockdown, and found ~255 lipid molecules changed significantly in the negative ion mode after FAAH interference (Fig. 6B, C). The content of LPA and FFA (especially FFA 20:4, also known as AA) decreased, while the content of AEA and phosphatidylethanolamine (PE) components increased (Fig. 6D). Then the content of different types of PGs in the cell supernatant were detected, and we discovered that the content of PGE2 decreased after interference with FAAH, but increased after FAAH overexpression (Fig. 6E). Moreover, after interference with FAAH, the expression of AEA-related enzymes increased, such as NAT-1 and NAPE-PLD, as well as the receptor of AEA (CB1/2). However, the enzymes involved in PG synthesis, COX-2, and LPA-induced hypoxia-inducible factor-1α (HIF-1α) decreased, suggesting that PGE2 synthesis was inhibited (Fig. 6F).

To verify whether miR-1275 was indirectly involved in the regulation of AEA/LPA signals, we treated MKN-1 cells with miR-1275 mimic, and observed that the content of PGE2 in the cell supernatant decreased, while the miR-1275 inhibitor increased PGE2 in HGC-27 cells (Fig. 6G). Further analysis showed that the miR-1275 mimic could reverse the changes in the FAAH-mediated AEA/LPA signal axis, manifested as a decrease in the content of HIF-1α and COX-2, and an increase in the expression of CB1/2, NAT-1, and NAPE-PLD, while the miR-1275 inhibitor showed the opposite trend in regulating the above signals (Fig. 6H).

## Discussion

GC remains prevalent worldwide and is associated with a high fatality rate, which may be significantly influenced by the hidden onset, delayed diagnosis, and biological heterogeneity of the tumour [11]. The occurrence and development of GC is a complex process involving epigenetics, metabolic regulation, and continuous changes in signalling pathways. Exploring the hydrolytic enzymes involved in lipid metabolism and key lipid signalling mediators is important in developing new drugs and implementing personalised treatment plans. In this study, we constructed a differential gene expression profile by whole transcriptome sequencing. We found that the hydrolase FAAH was highly expressed in GC tissues, which was also verified in TCGA and Oncomine databases. Further analysis by expanding the sample size indicated that high FAAH expression was significantly related to tumour size, invasion depth, and lymph node metastasis. Moreover, in analysing whether FAAH was tissue-specific, we noted that FAAH was enriched in GC tissues relative to other common solid tumours, especially tubular adenocarcinoma.

FAAH is a key hydrolase involved in regulating the endocannabinoid system. High expression of FAAH was reported to promote cell invasion and migration by hydrolysing 2-arachidonoylglycerol in prostate cancer [12]. Shubbar et al. [13] found that elevated levels of FAAH were positively correlated with the number of lymph node metastasis in breast cancer. FAAH gene polymorphism was reported to be associated with postoperative pain sensitivity in female patients with breast cancer [14]. In this study, we mainly explored the biological functions of FAAH in GC. Moreover, we discovered that silencing of FAAH led to slowed proliferation, metastasis, cycle progression, and increased cell apoptosis. Overexpression of FAAH showed a strong cancer-promoting effect, characterised by accelerated proliferation and cycle progression, enhanced metastasis, and decreased apoptosis. This phenomenon was also verified in animal models, which indicated that interference with FAAH could effectively inhibit tumour growth in vivo.

TCGA divided GC into four subtypes based on integrated genomics analysis. The lipid profile in tumours with chromosomal instability was significantly different from that in the adjacent tissues, especially the signals involved in the glycerophospholipid pathway [15, 16]. In our study, the expression level of FAAH was significantly higher in tumours with chromosomal instability than in normal controls, suggesting a relationship with FAAH-regulated lipid signalling. Further analysis of lipidomics showed that after stable interference with FAAH, the content of AEA increased while cancer-promoting molecules (AA and LPA) decreased, and the content of PE increased while FFA decreased. We also observed that the levels of inflammatory mediators represented by PGE2 in the cell supernatant decreased after interference with FAAH.

Intracellular LPA is an intermediate product of phospholipids and triacylglycerol, catalysed by peanut glycerol-3-phosphate acyltransferase. When the aerobic glycolysis pathway is inhibited, LPA creates a pseudo-hypoxic reaction and activates HIF-1α [17]. In breast cancer, HIF-1α enhanced the expression of COX-2 and induced PGE2 to activate vascular endothelial cells in a paracrine manner [18]. In addition, HIF-1α was confirmed to regulate the COX/PGE2 signalling axis in the zebrafish model [19]. AEA is a lipid mediator released on-demand by membrane PE through a two-step enzymatic reaction. AEA analogues induced morphological changes, decreased viability, and increased apoptosis of GC cells in a concentration-dependent manner [4]. AA, a type of omega-6 polyunsaturated fatty acid, is a metabolite produced by the hydrolysis of AEA by FAAH. Studies have confirmed that omega-6 fatty acids can be combined with PGE2 to enhance angiogenesis [20]. In our study, FAAH silencing induced increased levels of enzymes associated with AEA synthesis (NAT-1 and NAPE-PLD) and AEA receptor (CB1/2). The level of HIF-1α was reduced, suggesting that PGE2 synthesis was inhibited. Therefore, a cooperative regulation mechanism of the AEA/LPA signal mediated by FAAH emerged. On the one hand, silencing of FAAH inhibited the malignant progression of GC in three ways: blocking tumour suppressor signal AEA degradation, providing PE for the synthesis of AEA, and inhibiting downstream AA/COX-2/PGE2 signal activation. Conversely, after interference with FAAH, decreased FFA led to an insufficient substrate for the synthesis of LPA, which blocked the activation of COX-2/PGE2 signalling by HIF-1α and further inhibited the formation of tumours.

Certain miRNAs can regulate tumour lipid metabolism reprogramming by targeting key enzymes involved, thereby participating in the malignant progression of tumours. In breast cancer, miR-22 inhibits fatty acid synthesis and elongation in tumour cells by targeting ATP citrate lyase and fatty acid elongase 6 [21]. In addition, miR-31-5p could directly target the rate-limiting enzyme in β-oxidation (ester acyl-CoA oxidase) and upregulate the extracellular level of PGE2 to enhance cell metastasis [22]. Deregulated miR-1275 has been reported to be involved in the malignant progression of tumours. Lower expression of miR-1275 was reported in patients with liver metastases from colorectal cancer compared with those without metastasis [23]. In addition, miR-1275 inhibited EMT by activating Wnt/β-catenin signalling and enhanced the radiotherapy sensitivity of oesophageal cancer cells [24]. According to our data, miR-1275 had a complementary site for the 3’-UTR of FAAH. The IF-FISH assay revealed that FAAH and miR-1275 could be combined in the cytoplasm, but part of miR-1275 was expressed in the nucleus, which further explained that miR-1275 lost its control over FAAH in GC. Rescue experiments suggested that miR-1275 reversed the oncogenic effects on GC cells mediated by FAAH. As for lipid metabolism, miR-1275 downregulated HIF-1α and COX-2, and upregulated the levels of CB1/2, NAT-1, and NAPE-PLD, suggesting that miR-1275 could negatively regulate lipid signalling in the AEA/LPA pathway by targeting FAAH.

In summary, highly expressed FAAH promotes the malignant progression of GC, and miR-1275 is an upstream molecule with a negative regulatory effect on FAAH. Low abundance of miR-1275 in GC failed to target FAAH, leading to FAAH accumulation, which could mediate lipid metabolism reprogramming through the coordinated regulation of AEA/LPA signals.

## Materials and methods

### Whole transcriptome sequencing

Three pairs of fresh GC tissues and adjacent tissues were selected for RNA-seq (not included in the 96 pairs of tissues that were subsequently collected). Total RNA was isolated using a HiPure Total RNA Mini Kit (Magen, Germany). Concentration and integrity were measured using a Qubit 3.0 fluorometer (Invitrogen, Carlsbad, USA) and Agilent 2100 Bioanalyzer (Applied Biosystems, Carlsbad, USA), respectively. The RNA-seq library was prepared with ~2 μg of total RNA using the KAPA RNA HyperPrep Kit with RiboErase (HMR) for Illumina® (Kapa Biosystems, Inc., Woburn, MA) according to the manufacturer’s instructions. The Bioanalyzer 2100 system (Agilent Technologies, USA) was used to detect the size range, and qRT-PCR was used to quantify the effective concentration of the library. After the library was qualified, 10 nM was pooled equimolar prior to clustering. paired-end (PE150) sequencing was performed on all samples.

### Lentiviral infection

The two shRNA fragments with high knockdown efficiency (shFAAH-1416 and shFAAH-1557) were packed into pGV112-GFP vector (GeneChem, Shanghai, China) and co-transfected 293T cells with pHelper1.0 and pHelper2.0 plasmids to produce lentivirus. An appropriate amount of lentivirus was added to the cells according to the multiplicity of infection (MOI = 50) until 20% confluence was reached. After 72 h of infection, GFP-tagged gene expression was observed under a fluorescence microscope, followed by selection with 2 mg/ml puromycin (MCE, New Jersey, USA) for 2 weeks to obtain stable FAAH knockdown cells.

### Dual-luciferase reporter assay

The 3’-UTR sequences of FAAH were inserted into a pmiR-RB-ReportTM vector (RiboBio, Guangzhou, China), and then transformed and amplified using DH5α. The extracted plasmid was identified using double-restriction enzyme digestion. After site-directed mutagenesis, wild-type and mutant-type recombinant plasmids were constructed, and either of which carrying mimic-NC or miR-1275-mimic were co-transfected into MKN-1 cells. After 48 h of transfection, firefly luciferase/renilla luciferase were detected using the Dual-Luciferase Reporter Assay System (Promega, Madison, USA).

### Immunofluorescence-fluorescence in situ hybridization (IF-FISH) assay

Cells were seeded on glass coverslips at a density of 3 × 10^5^/mL and cultured for 24 h. Then 2 mL of 4% paraformaldehyde was added to fix the cells for 30 min. After washing with DEPC water twice, 1 mL of permeabilization solution was added and incubated at room temperature for 10 min. After washing three times with PBS, 1% paraformaldehyde was used to fix for 10 min. Then 70, 80, 95, and 100% ethanol were used for gradient dehydration. After 30 min of pre-hybridization, the hybridization system containing the miR-1275 probe (BersinBio, Guangzhou, China) was added, and then the glass coverslips were transferred to 73 °C for co-denaturation, and quickly transferred to 37 °C for overnight hybridization. After washing, cells were blocked with 5% BSA for 30 min and incubated with anti-FAAH (Affinity Biosciences, Cincinnati, USA) at 4 °C overnight. On the second day, after incubating with the fluorescent secondary antibody for 30 min, DAPI was used to stain the nucleus.

### Xenograft model

A total of 20 male BALB/c nude mice (aged 5–6 weeks) were purchased from the Laboratory Animal Center of Nantong University and randomly divided into five groups. The experimental procedures and animal care were approved by the Animal Experimental Ethics Committee of Nantong University. Approximately 2 × 10^7^ MKN-1 cells were resuspended in 200 μL of RPMI-1640 medium and inoculated subcutaneously on the dorsal side of nude mice. Since the day of inoculation, the long (*a*) and short diameters (*b*) of the tumour were measured every 2 days to calculate the volume (*V* = *ab*^2^/2) and draw the growth curve. After 10 days of inoculation, fluorouracil was injected intraperitoneally every 2 days for a total of 10 injections to construct a clinical chemotherapy drug treatment model. The nude mice were killed by cervical dislocation ~30 days after inoculation, and the tumours were completely removed. The size and weight of the tumours were recorded, and qRT-PCR was used to detect the expression of related genes.

### Liquid chromatography with tandem mass spectrometry for lipidomics

About 1 mL of pre-cooled 60% methanol aqueous solution (chromatographic grade) was used to resuspend the cells and centrifuged at 1000 × *g* for 1 min to retain the cell pellet. To extract lipids, 100 μL of water, 200 μL of pre-cooled methanol, 400 μL of dichloromethane, and 120 μL of water were added to the cell pellet (50 mg) in sequence. Vortex mixing was performed for each step. After incubation at 26 °C for 20 min, the suspension was centrifuged at 8000 × *g* at 10 °C for 15 min to remove the lower organic phase and then dried with nitrogen. For mass spectrometry analysis, 100 µL of isopropanol solution was added for reconstitution, and the supernatant was collected after 15 min of centrifugation at 8000 × *g*. The samples were separated using an ACQUITY UPLC® I-Class system (Waters, Milford, USA). Mass spectrometry was performed using electrospray ionisation positive and negative ion modes. After separation by high-performance liquid chromatography, the samples were analysed using Xevo G2-XS QTof (Waters, Milford, USA). Progenesis QI software (Waters, Milford, USA) was used for raw data analysis.

### Statistical analysis

Statistical analyses were performed using SPSS (Version 23.0; IBM, Armonk, USA) and GraphPad Prism software (Version 7; La Jolla, USA). Each experiment was repeated three times, and the data were represented as the mean ± SD. Student’s *t* test or one-way analysis of variance was used for statistical analysis when appropriate. The paired *t*-test was used to compare paired samples. The Mann–Whitney *U* test and one-way ANOVA were applied to determine the correlations between FAAH expression and clinicopathological characteristics of patients with GC. Spearman’s correlation analysis was used to evaluate the correlation between FAAH and miR-1275 expression. The grey value of protein bands and the proportion of positive cells in experiments such as EdU, immunohistochemistry, and immunofluorescence assays were quantified using ImageJ software (NIH, Bethesda, USA). Statistical significance was set at p < 0.05.

## Supplementary information

Supplementary Figure S1

Supplementary Figure S2

Supplementary Figure S3

Supplementary Figure S4

Supplementary figure legends

Supplementary tables

Supplementary materials and methods

Supplementary material for original western blots

aj-checklist

## Data Availability

The datasets used and/or analysed during the current study are available from the corresponding author on reasonable request.
