# Supplementary figures and images for: MicroRNA miR-1275 coordinately regulates AEA/LPA signals via targeting FAAH in lipid metabolism reprogramming of gastric cancer
Source: Cell Death Dis. 2023 Jan 26;14(1):62. doi: 10.1038/s41419-023-05584-8 (PMC9879949; doi:10.1038/s41419-023-05584-8)

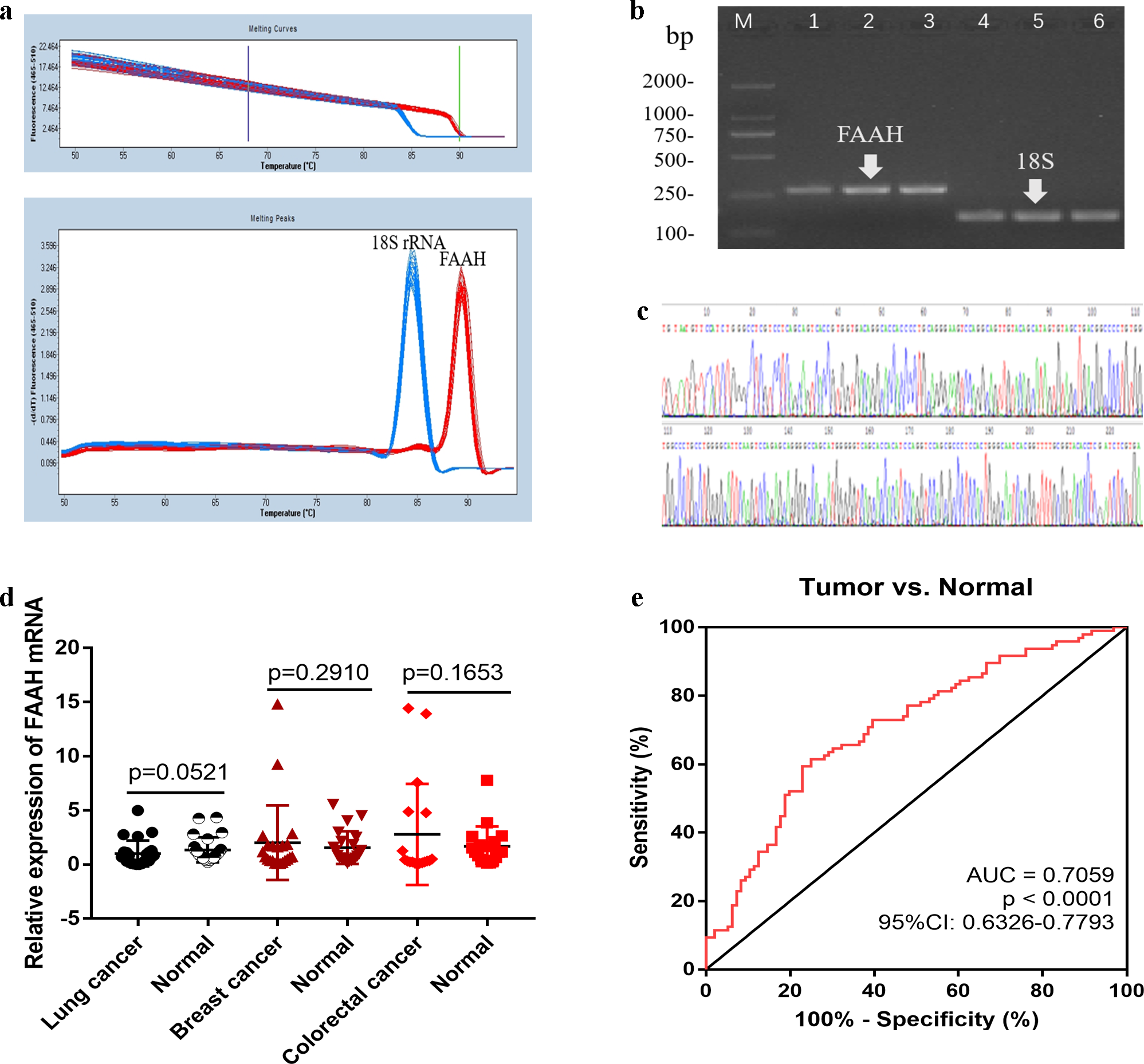

Supplement: Supplementary file 1 — Supplementary Figure S1 [file 41419_2023_5584_MOESM1_ESM.tif]

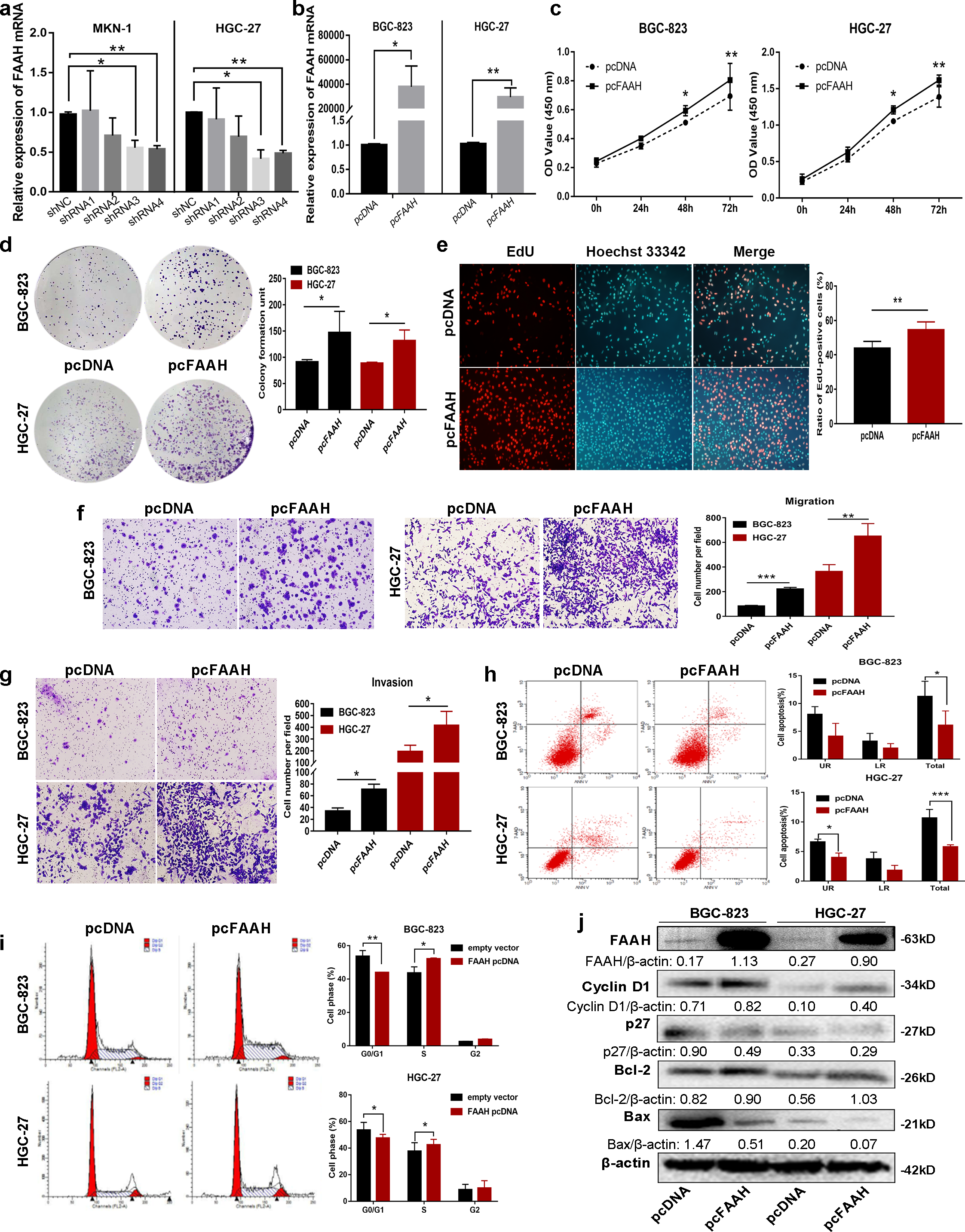

Supplement: Supplementary file 2 — Supplementary Figure S2 [file 41419_2023_5584_MOESM2_ESM.tif]

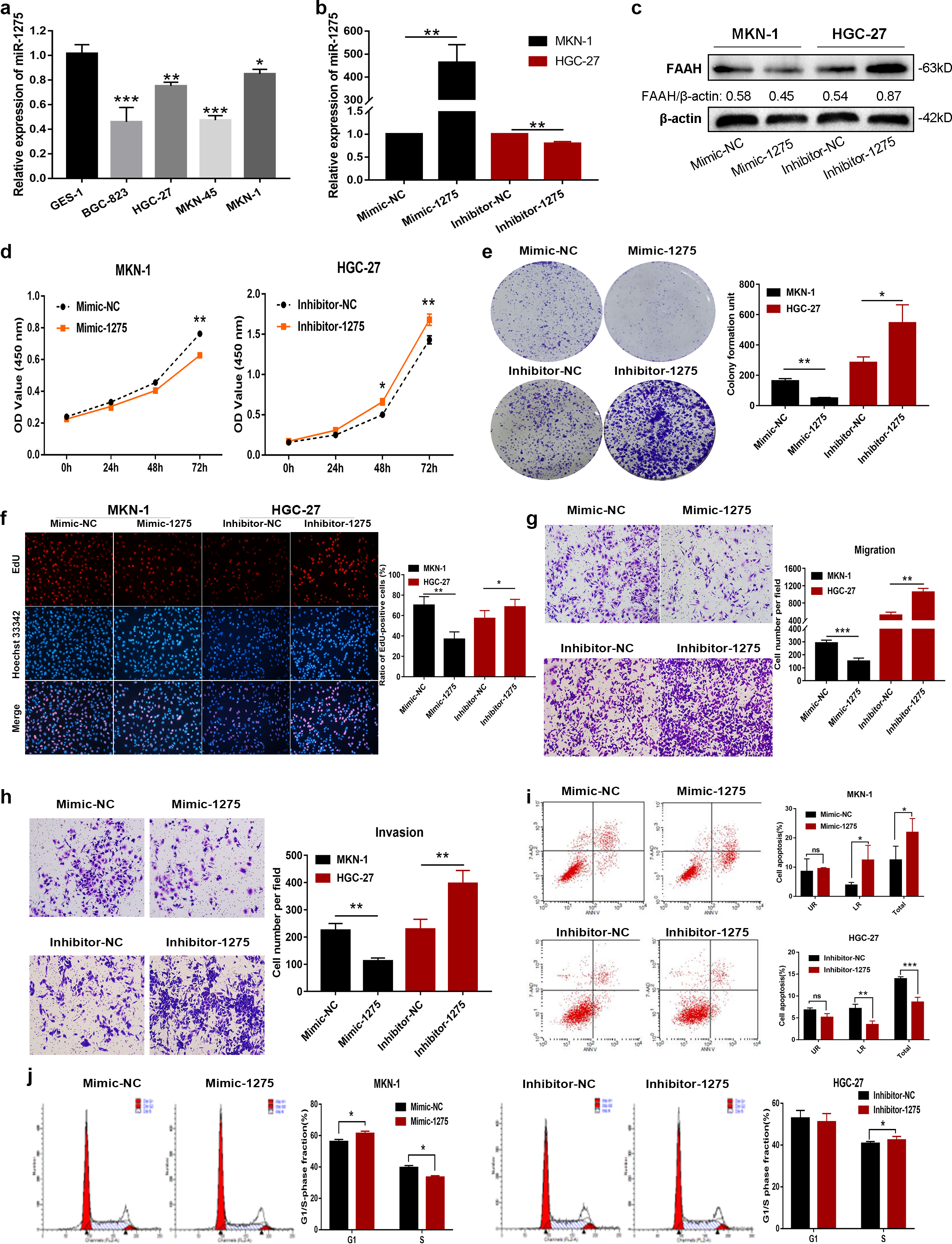

Supplement: Supplementary file 3 — Supplementary Figure S3 [file 41419_2023_5584_MOESM3_ESM.tif]

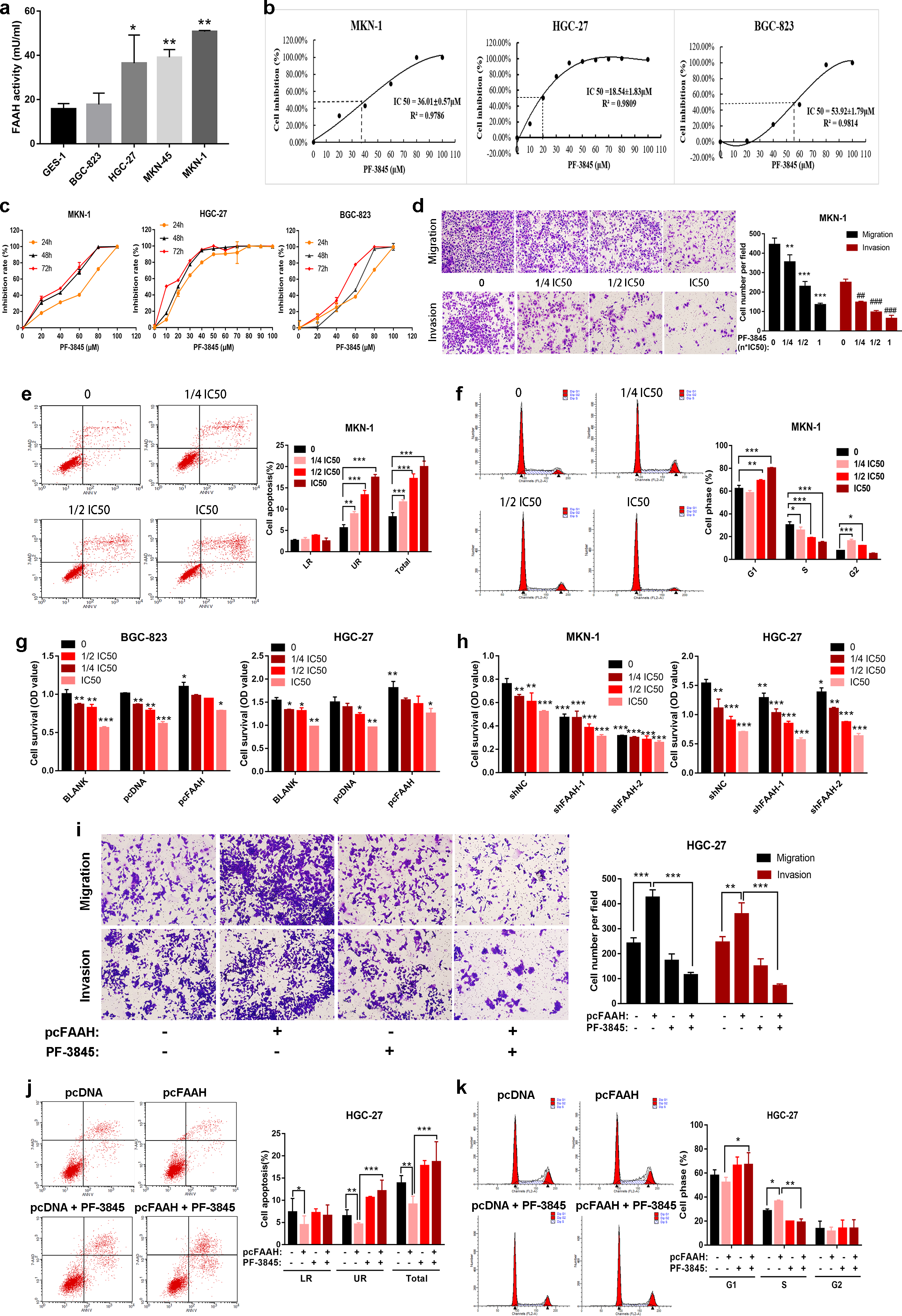

Supplement: Supplementary file 4 — Supplementary Figure S4 [file 41419_2023_5584_MOESM4_ESM.tif]
