# Supplementary material for: MicroRNA miR-1275 coordinately regulates AEA/LPA signals via targeting FAAH in lipid metabolism reprogramming of gastric cancer
Source: Cell Death Dis. 2023 Jan 26;14(1):62. doi: 10.1038/s41419-023-05584-8 (PMC9879949; doi:10.1038/s41419-023-05584-8)
Supplement: Supplementary file 5 — Supplementary figure legends [file 41419_2023_5584_MOESM5_ESM.docx]

**Supplementary Figure legends**

**Figure S1 Feasibility analysis of detecting FAAH.** (**a**) The single-peak melting curve confirmed that the molecule was specifically amplified without non-specific amplification due to primer-dimerization. (**b**) The size of the amplified product was detected by agarose gel electrophoresis (FAAH：262bp；18s rRNA: 151bp). (**c**) The sequence of the amplified product was verified by Sanger sequencing. (**d**) Detection of FAAH expression in lung cancer (n=24 pairs), breast cancer (n=22 pairs) and colorectal cancer (n=18 pairs) tissues by qRT-PCR. (**e**) The diagnostic efficacy of FAAH in GC was evaluated using ROC curve.

**Figure S2 Highly expressed FAAH promotes the malignant phenotype of GC cells in vitro.** The transfection efficiency of (**a**) four interfering vectors and (**b**) the overexpression vector in GC cell lines was detected by qRT-PCR. (**c**) Cell proliferation was detected by CCK-8 after overexpression of FAAH. (**d**) Cell colony formation assay. (**e**) The proportion of proliferating cells (labeled with red fluorescence) was detected by EdU after overexpression of FAAH. Transwell assay was used to evaluate the ability of (**f**) migration and (**g**) invasion after overexpression of FAAH. The proportion of (**h**)apoptotic cells and (**i**) cycle distribution after overexpression of FAAH were detected Using flow cytometry. (**j**) Western blot was used to detect changes of apoptosis- and cycle-related proteins after overpressing FAAH. *p<0.05, **p<0.01, ***p<0.001.

**Figure S3 miR-1275 exerts a tumour suppressor effect in GC cells.** (**a**) The expression of miR-1275 in four GC cell lines. (**b**) Transfection efficiency of miR-1275 mimic and inhibitor in GC cells. (**c**) Changes of FAAH expression in MKN-1 and HGC-27 cells after transfection with miR-1275 mimic and inhibitor. After transfection of miR-1275 mimic and inhibitor, the ability of cell proliferation was detected by (**d**) CCK-8, (**e**) colony formation assay and (**f**) EdU. The effect of miR-1275 on (**g**) migration and (**h**) invasion was detected. The flow cytometry was used to detect the effect of miR-1275 on (**i**) apoptosis and (**j**) cycle distribution. *p<0.05, **p<0.01, ***p<0.001.

**Figure S4 PF-3845 reverses the oncogenic effects of FAAH by inhibiting its activity.** (**a**) Detection of FAAH activity in four GC cells. (**b**) IC_50_ determination of PF-3845 in three GC cells. (**c**) The cell inhibition rate was detected by CCK-8 after treatment with different concentrations of PF-3845 for 24h, 48h and 72h, respectively. (**d**) Detection of cell migration and invasion after treatment with different concentrations of PF-3845; Flow cytometry was used to detect the effects of different concentrations of PF-3845 on (**e**) apoptosis and (**f**) cell cycle. The changes of cell proliferation were detected by CCK-8 after co-treatment with different concentrations of PF-3845 and (**g**) pcFAAH or (**h**) shFAAH. (**i**) Cell migration and invasion were detected after co-treatment with pcFAAH and PF-3845 (concentration: IC_50_). The changes of (**j**) apoptosis and (**k**) cycle distribution were detected after co-treatment with pcFAAH and PF-3845 (concentration: IC_50_). *p<0.05, **p<0.01, ***p<0.001, ^##^p<0.01, ^###^p<0.001.
