# Supplementary material for: MicroRNA miR-1275 coordinately regulates AEA/LPA signals via targeting FAAH in lipid metabolism reprogramming of gastric cancer
Source: Cell Death Dis. 2023 Jan 26;14(1):62. doi: 10.1038/s41419-023-05584-8 (PMC9879949; doi:10.1038/s41419-023-05584-8)
Supplement: Supplementary file 6 — Supplementary tables [file 41419_2023_5584_MOESM6_ESM.docx]

| **Supplementary Tables**  **Table S1 The sequences of primers used in this study.** | | |
| --- | --- | --- |
| **Gene** | **Forward primer (5’ - 3’)** | **Reverse primer (5’ - 3’)** |
| FAAH | CACGAGATCGAGGTGTACCG | ATCCCCTTCTGCAGCATCTT |
| 18S rRNA | GTAACCCGTTGAACCCCATT | CCATCCAATCGGTAGTAGCG |
| N-cadherin | CGATAAGGATCAACCCCATACA | TTCAAAGTCGATTGGTTTGACC |
| E-cadherin | AGTCACTGACACCAACGATAAT | ATCGTTGTTCACTGGATTTGTG |
| Vimentin | CCTTCGTGAATACCAAGACCTGCTC | AATCCTGCTCTCCTCGCCTTCC |
| Snail | CCTCGCTGCCAATGCTCATCTG | AGCCTTTCCCACTGTCCTCATCTG |

| **Table S2 The sequences of shRNAs for FAAH interference.** | | |
| --- | --- | --- |
| **Gene** | **Alias** | **Sequence (5’ - 3’)** |
| shRNA1 | shFAAH-978 | GCGAGGACATGTTCCGCTTGG |
| shRNA2 | shFAAH-1123 | GGAGACCAAACAGAGCCTTGA |
| shRNA3 | shFAAH-1416 | GGGAACTGCAGCACGAGATCG |
| shRNA4 | shFAAH-1557 | GCTACACTATGCTGTACAACT |

**Table S3 List of abbreviations used in the manuscript.**

| **Abbreviation** | **Full name** |
| --- | --- |
| AA | arachidonic acid |
| AEA | arachidonoyl ethanolamide |
| CB1/2 | cannabinoid receptor 1/2 |
| c-caspase 3 | Cleaved caspase 3 |
| CIN | chromosomal instability |
| COX-1/2 | cyclooxygenase-1/2 |
| EBV | Epstein-Barr virus |
| EMT | epithelial-mesenchymal transition |
| FAAH | fatty acid amide hydrolase |
| FDR | false discovery rate |
| FFA | free fatty acid |
| GC | gastric cancer |
| GPAT | glycerol-3-phosphate acyltransferase |
| GS | genetically stable |
| HIF-1α | hypoxia-inducible factor 1α |
| IF-FISH | immunofluorescence-fluorescence in situ hybridization |
| IHC | Immunohistochemistry |
| IQR | interquartile range |
| LPA | lysophosphatidic acid |
| MSI | microsatellite unstable |
| NAPE-PLD | N-acylphosphatidylethanolamine-phospholipases D |
| NAT-1 | N-acetyltransferase 1 |
| NES | normalized enrichment score |
| PE | Phosphatidylethanolamine |
| PG | prostaglandin |
| PGE2 | prostaglandin E2 |
| qRT-PCR | quantitative real-time polymerase chain reaction |
| TUNEL | Terminal-deoxynucleotidyl transferase mediated nick end labeling |
