# Supplementary material for: MicroRNA miR-1275 coordinately regulates AEA/LPA signals via targeting FAAH in lipid metabolism reprogramming of gastric cancer
Source: Cell Death Dis. 2023 Jan 26;14(1):62. doi: 10.1038/s41419-023-05584-8 (PMC9879949; doi:10.1038/s41419-023-05584-8)
Supplement: Supplementary file 7 — Supplementary materials and methods [file 41419_2023_5584_MOESM7_ESM.docx]

**Supplementary Materials and Methods**

**Human specimens and cell lines**

99 pairs of GC tissues from newly diagnosed GC patients who underwent tumour resection in the Affiliated Hospital of Nantong University from December 2017 to December 2019 were involved in this study, of which 3 pairs of tissues were used for high-throughput sequencing and 96 pairs of tissues were used for PCR validation. This study was approved by the Ethics Committee of the Affiliated Hospital of Nantong University, and all participants provided informed consent.

GC cell lines (BGC-823, HGC-27, and MKN-45) and normal human gastric epithelial cell lines were purchased from ATCC. MKN-1 cells were purchased from Cobioer Biosciences Co., Ltd. (Nanjing, China). All cells were cultured in DMEM/RPMI-1640 (Invitrogen, Carlsbad, USA) containing 10% fetal bovine serum (Gibco, Invitrogen, USA) and 1% penicillin-streptomycin solution (Sangon Biotech, Shanghai, China) at 37°C in 5% CO2.

**RNA extraction and quantitative real-time PCR (qRT-PCR)**

Total RNA of GC tissues (40 mg) or cells was extracted by TRIzol reagent (Invitrogen, Carlsbad, USA). After measuring the concentration, 1000ng of total RNA was taken for reverse transcription reaction using RevertAid First Strand cDNA Synthesis Kit (ThermoFisher Scientific, Waltham, USA). The qRT-PCR was performed on the LightCycler® 480 SYBR GreenⅠMaster (Roche, Basel, Switzerland), and the reaction conditions were pre-amplification at 95°C for 10 min, followed by 40 cycles of amplification (15s at 95°C, 30s at 60°C, and 30s at 72°C). Sequences of the primers were listed in **Supplementary Table S1**. The 18S rRNA was used as the internal reference for mRNA because of its stability. Primers for miRNAs (including one RT primer and a pair of qRT-PCR primers for each miRNA) were designed by RiboBio (Guangzhou, China). The U6 snRNA was used to normalize miRNA levels.

**Total protein extraction and western blot**

Tissues or cells were lysed in the lysate mixture, where the ratio of RIPA and PMSF was 100:1 (NCM Biotech, Suzhou, China). The concentration of protein was detected by BCA Protein Assay Kit (Beyotime, Shanghai, China). During electrophoresis, 40 μg of protein was added into each well, and then separated by electrophoresis on 10% SDS-PAGE gels (NCM Biotech, Suzhou, China). After transferring onto a PVDF membrane (Millipore, Schwalbach, Germany), separated protein bands were blocked on a shaker with 5% skimmed milk for 2 h. The primary antibodies were respectively added and incubated at 4°C overnight. Then the membrane was immunoblotted with a corresponding HRP-labeled secondary antibody for 1 h at room temperature. Finally, the blots were detected by NcmECL High kit (NCM Biotech, Suzhou, China) using ChemiDoc XRS system (Hercules, CA, USA).

**Plasmid construction and transfection**

The interference vector (shRNA1, 2, 3 and 4) and overexpression vector (pcFAAH) with their respective controls (shNC and pcDNA) were designed by GenePharma (Suzhou, China). The sequences of shRNAs were shown in **Supplementary Table S2**. As for miRNAs, both mimic and inhibitor with their respective negative controls were designed by RiboBio (Guangzhou, China). Lipofectamine 3000 (Invitrogen, Carlsbad, USA) was used for cell transfection when 60–80% confluence was obtained.

**Cell Counting Kit-8 (CCK-8) and colony formation assay**

GC cells were seeded in a 96-well plate at a density of 3000 cells per well. About 12 h after plating, 10 µL of CCK-8 (Dojindo, Kumamoto, Japan) was added into each well at 0, 24, 48, and 72 h after adhesion, respectively. After incubation at 37 °C for 2 h, the optical density (OD) value was measured at 450 nm.

For colony-forming assay, cells were seeded in a 6-well plate at a concentration of 1000 per well and cultured at 37°C for 15 days. The colonies were then fixed with methanol and stained with 0.1% crystal violet (Beyotime, Shanghai, China). Visible colonies were counted manually.

**EdU assay**

The BeyoClick™ EdU Cell Proliferation Kit with Alexa Fluor 555 (Beyotime, Shanghai, China) was used according to the manufacturer's instructions. GC cells were seeded on circular slides in 24-well plates at a density of 1×10^4^ cells per well overnight. EdU solution (20 μM) was added to each well and incubated for 2 h. The cells were then fixed with 4% formaldehyde for 15 min and subsequently permeabilized with 0.5% Triton X-100 for 10 min. Then 125 µL of click reaction solution was added into each well and incubated for 30 min in the dark. Finally, 125 μL of Hoechst 33342 solution was added to stain the nuclei. Images were captured to calculate the percentage of EdU-positive cells.

**Transwell assay**

After 48 h of transfection, cells were resuspended in RPMI-1640 medium and the cell density was adjusted to 5×10^5^/ml. For the invasion assay, the upper chamber was pre-coated with diluted Matrigel (BD Pharmingen, San Diego, USA) at a ratio of 1:6 (50μL Matrigel mixed with 300 μL culture medium). After coagulation of 5 h, 100 μL of cell suspension was added into upper chamber (8 μm, Corning, NY, USA). Then 500 μL of 20% FBS-containing medium was added into the bottom chamber to attract cells to migrate. After 24 h of incubation, cells migrating through the membrane were ﬁxed by 4% formaldehyde, stained with 0.1% crystal violet (Beyotime, Shanghai, China). The average of stained cells was calculated by selecting five random fields using an optical microscope (Olympus, Tokyo, Japan).

**Apoptosis and cell cycle assay**

After 48 h of transfection, cells were harvested by trypsin and washed three times with pre-chilled PBS. The apoptosis assay was performed using the Annexin V-PE/7-AAD Apoptosis Detection Kit (BD Pharmingen, San Diego, USA) according to the manufacturer's protocol. For the cell cycle assay, the cells were fixed with 70% alcohol overnight at 4 °C, and washed three times with pre-chilled PBS the next day to remove ethanol. The DNA Content Quantitation Assay (Cell Cycle) kit (Solarbio, Beijing, China) was used according to the manufacturer's protocol. Both experiments were analyzed by flow cytometry (BD Pharmingen, San Diego, USA).

**Immunohistochemistry (IHC)**

IHC was performed on paraffin sections of animal tumours using IHC kit (Servicebio, Wuhan, China) according to manufacturer’s protocol. Primary antibodies against ki67 (1:200, ab16667, Abcam) and Cleaved caspase-3 (1:400, #9661, Cell Signalling Technology) were used. The pictures of positive cells were captured using an optical microscope (Olympus, Tokyo, Japan).

**Terminal-deoxynucleotidyl transferase mediated nick end labeling (TUNEL)**

TUNEL was performed on paraffin sections of animal tumours using the Fluorescein (FITC) Tunel Cell Apoptosis Detection Kit (Servicebio, Wuhan, China) according to manufacturer’s protocol. The fluorescence microscope (Olympus, Tokyo, Japan) with a magnification of 400 times was used to take pictures.

**Determination of FAAH activity in GC cells**

The activity of FAAH was determined by the FAAH Activity Assay Kit (Fluorometric, BioVision, Milpitas, USA) according to manufacturer’s instructions. Cellular FAAH hydrolyzed a non-fluorescent substrate releasing 7-amino-4-methylcoumarin (AMC), which could be measured at Ex/Em= 360/465 nm. The stable fluorescence signal was detected using FluoroskanTM Plate Reader (Thermo Fisher Scientific, Waltham, USA). The activities of FAAH in samples were then calculated using the following formula: B/(∆t × V) × D (where B was AMC amount from standard curve, ∆t was reaction time, V was sample volume, D was dilution factor).

**IC_50_ determination of PF-3845**

GC cells (5×10^4^/ml) were seeded into 96-well plates in a volume of 100 μL per well.

Different concentrations (0, 20, 40, 60, 80 and 100μM) of PF-3845 (MedChem Express, Monmouth Junction, USA) were added and co-incubated with cells for 24, 48 and 72 h. At the reaction time, 10 μL of CCK-8 kit was added to each well and incubated at 37 °C for 2 h. The OD value was measured on a microplate reader at a wavelength of 450 nm.

**Detection of prostaglandins (PGs) in the cell supernatant**

The content of various PGs (including total PG, PGE2, PGD2, PGI2 and PGF2α) in the cell supernatant were measured by ELISA (J&L Biological, Shanghai, China) following the manufacturer’s protocols. The cell supernatant was collected and centrifuged at 1000g for 20 min to discard the precipitate, and 50 μL of cell supernatant or standard with different concentration were added into the respective wells. Then 100 μL of HRP-labeled antibody was added to each well and incubated at 37°C for 60 min in a hybridization chamber. After washing three times, 50μL of Substrate A and Substrate B were added into each well in turn. After 15 min of incubation at 37°C, the reaction was stopped and the OD values were recorded at a wavelength of 450 nm. Finally, the sample concentration was calculated from the standard curve.
