# Supplementary material for: MicroRNA miR-1275 coordinately regulates AEA/LPA signals via targeting FAAH in lipid metabolism reprogramming of gastric cancer
Source: Cell Death Dis. 2023 Jan 26;14(1):62. doi: 10.1038/s41419-023-05584-8 (PMC9879949; doi:10.1038/s41419-023-05584-8)

Supplementary material for original western blots

NcmColor Color Prestained Protein Ladders (New Cell & Molecular Biotech Co. Ltd, Suzhou, China) were selected as protein ladders for Western blot experiments.

Figure 1F

Tissue No. 1-8 **FAAH (63kD)**

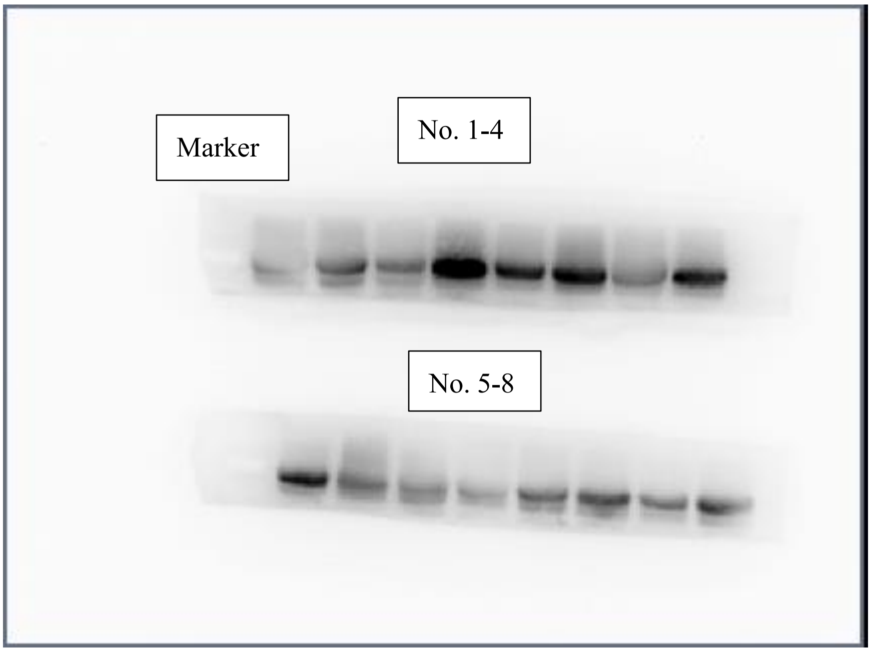

Tissue No. 1-8 **β-actin (42 kD)**

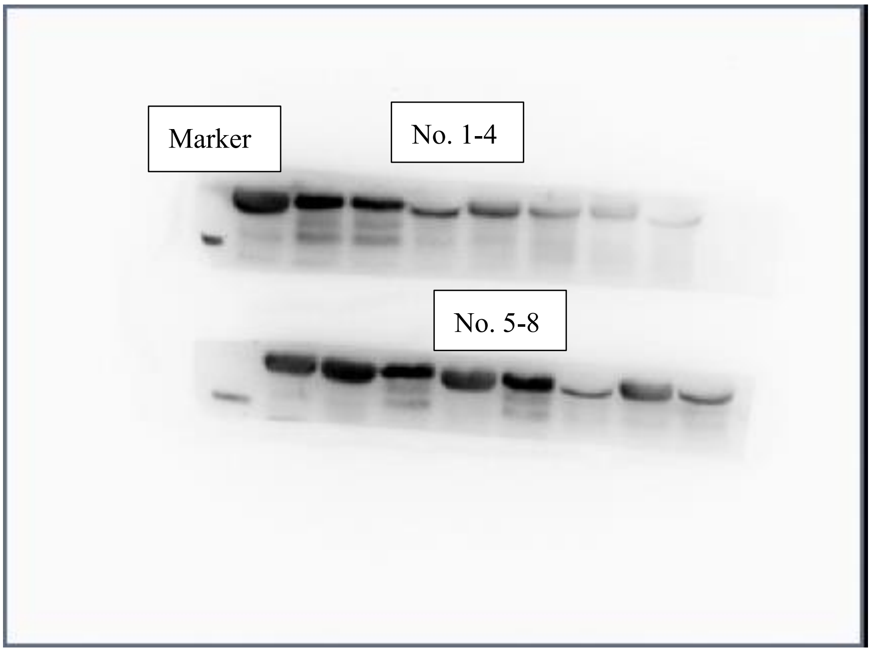

Tissue No. 9-16 **FAAH (63kD)**

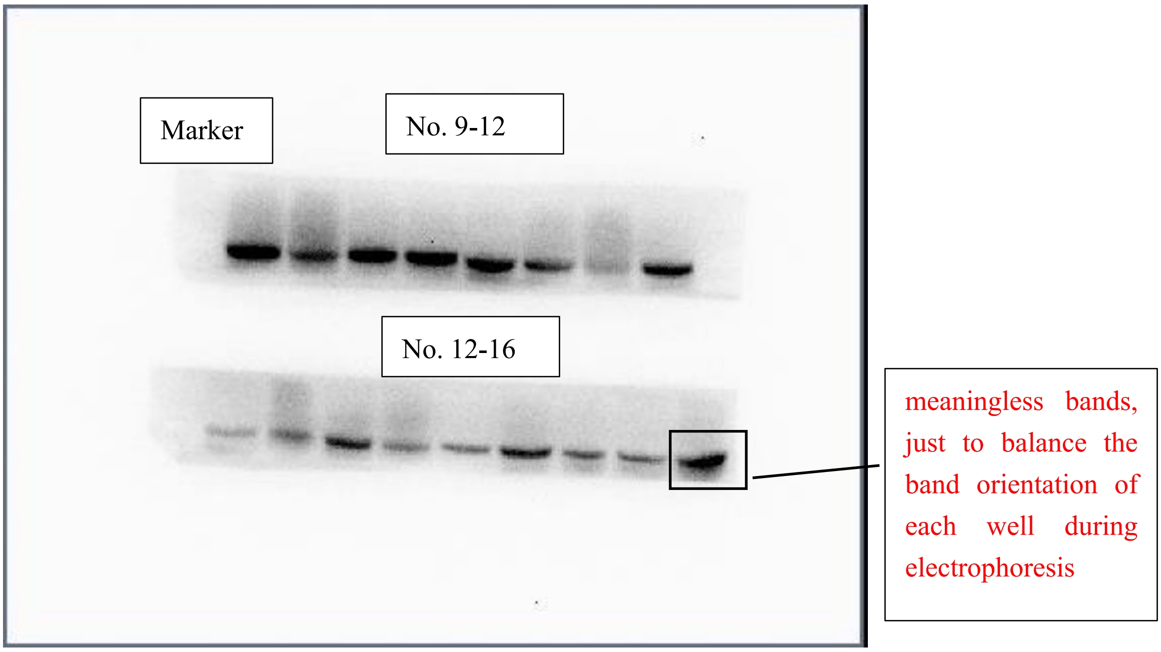

Tissue No. 9-16 **β-actin (42 kD)**

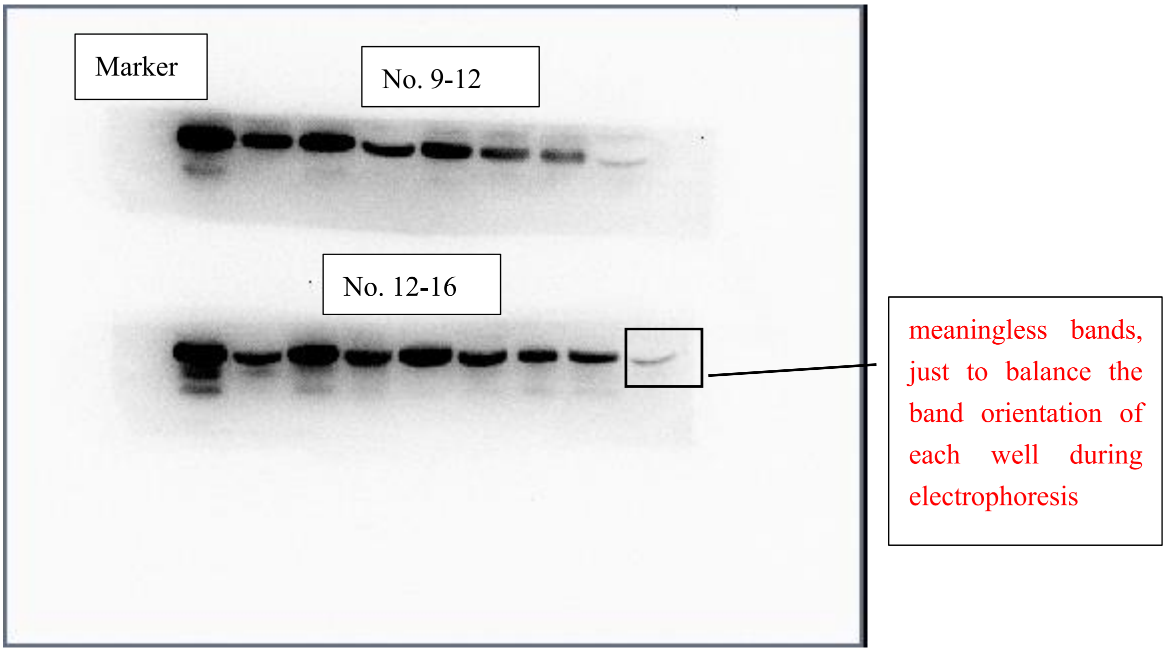

Tissue No. 17-20
Up: **β-actin (42 kD)**
Down: **FAAH (63kD)**

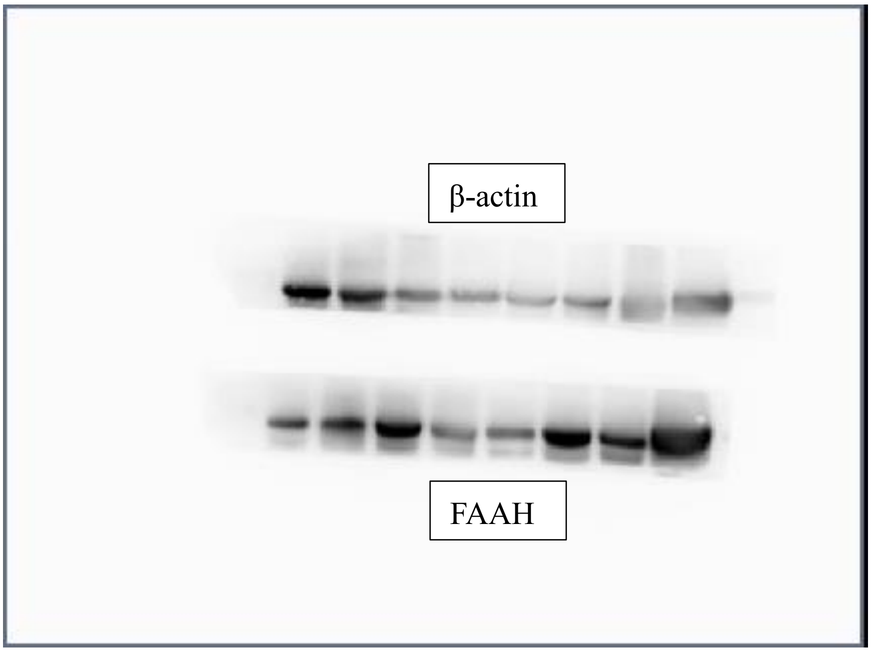

Figure 2A

FAAH (63kD)

Repeat test 1

Left to right: GES-1, BGC-823, HGC-27, MKN-45, MKN-1)

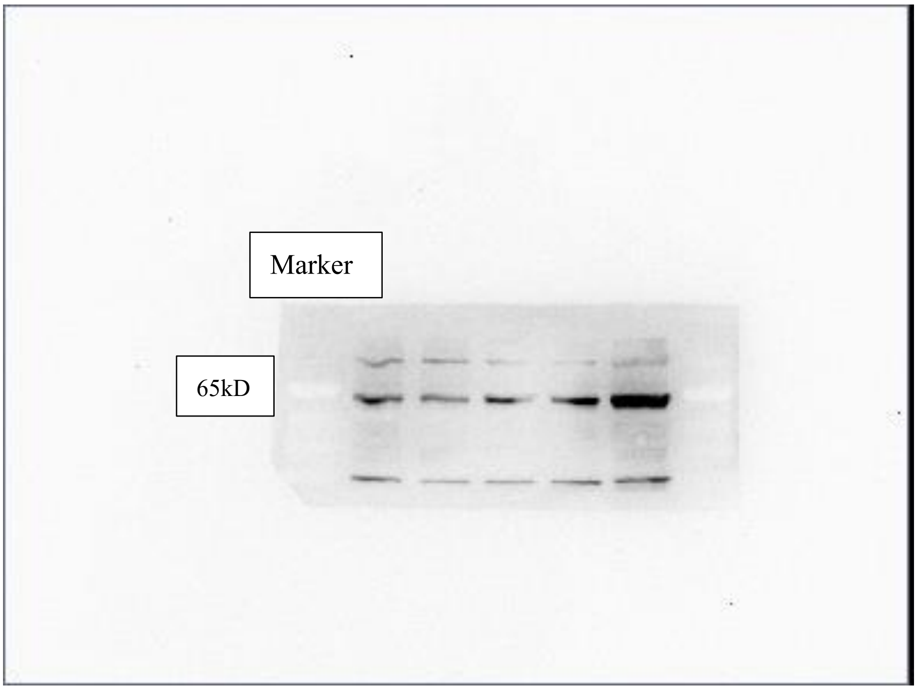

Repeat test 2

Left to right: GES-1, BGC-823, HGC-27, MKN-45, MKN-1)

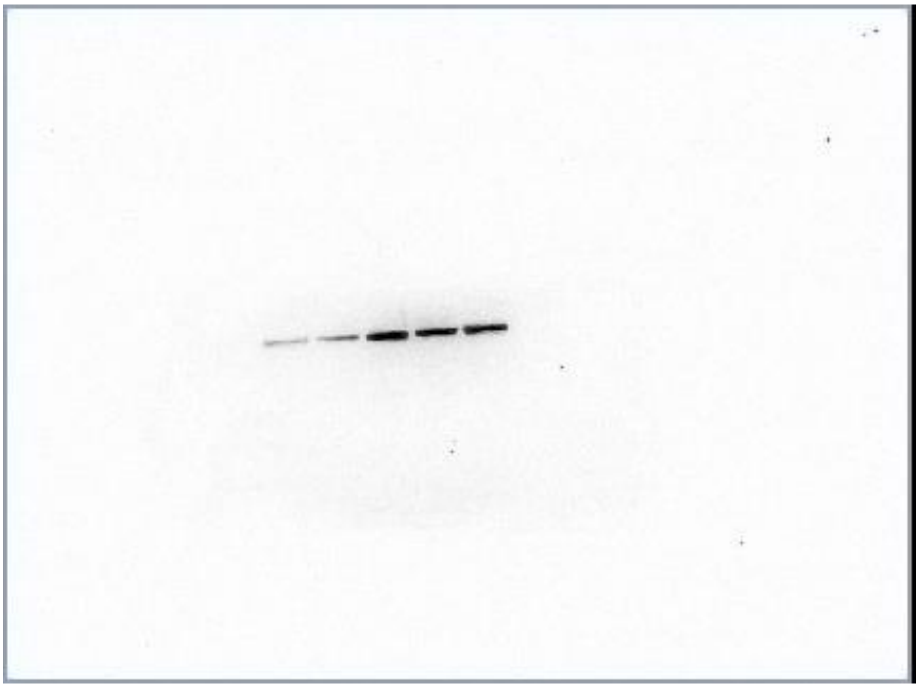

Repeat test 3

Left to right: GES-1, BGC-823, HGC-27, MKN-45, MKN-1)

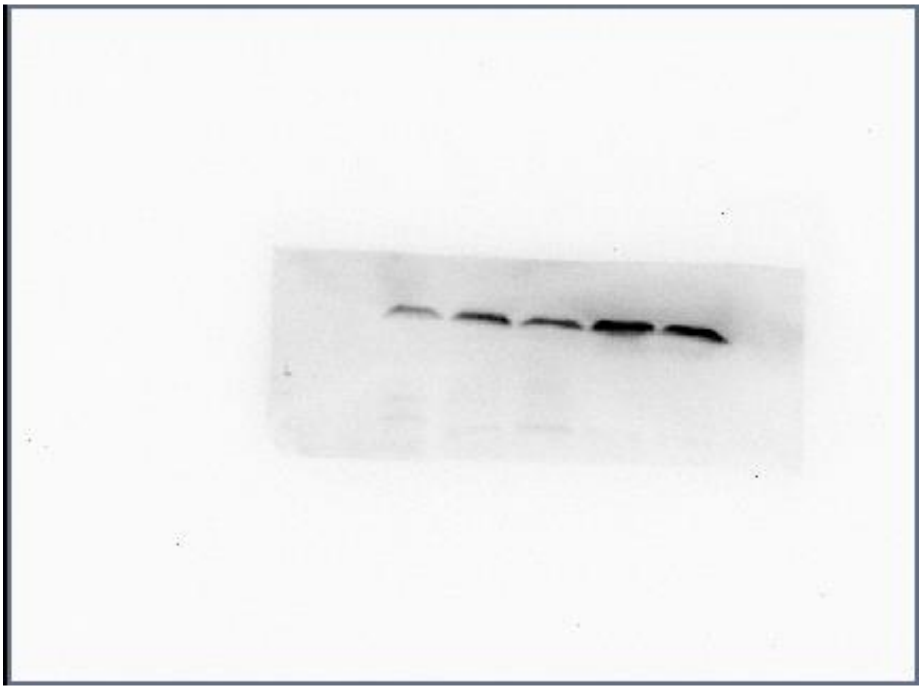

β-actin (42 kD)

Repeat test 1

Left to right: GES-1, BGC-823, HGC-27, MKN-45, MKN-1)

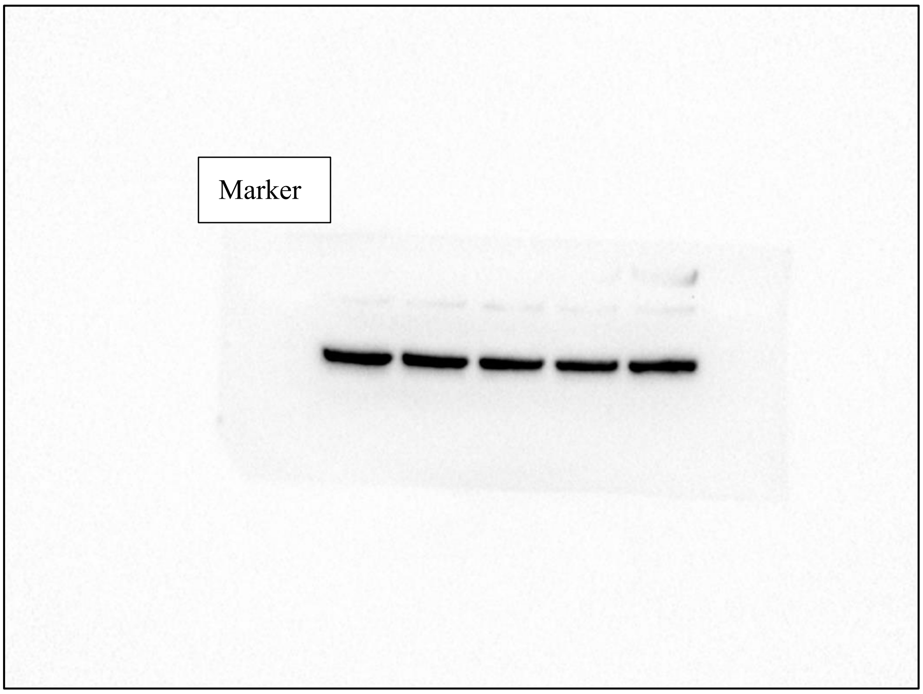

Repeat test 2

Left to right: GES-1, BGC-823, HGC-27, MKN-45, MKN-1)

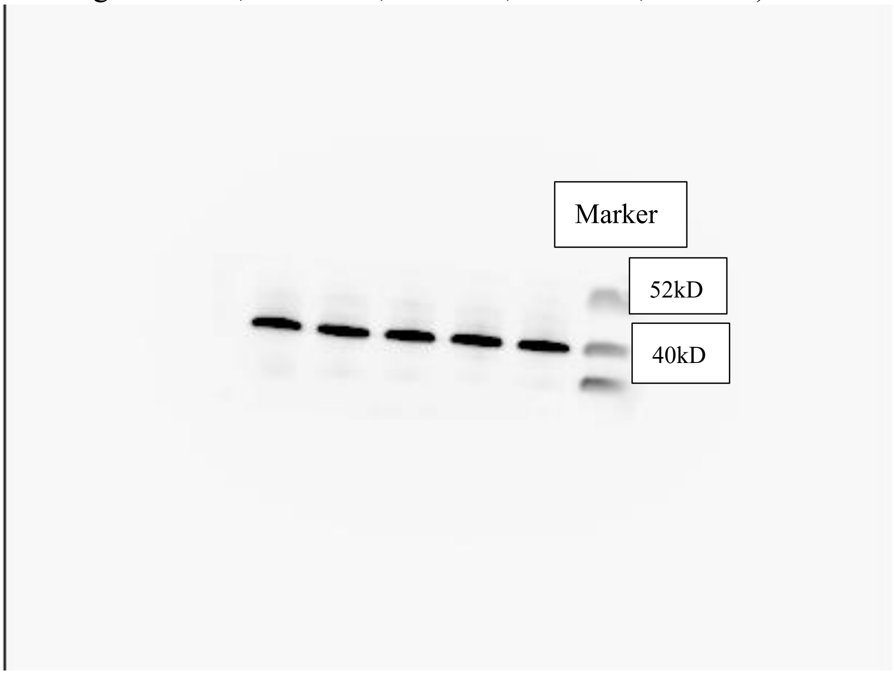

Repeat test 3

Left to right: GES-1, BGC-823, HGC-27, MKN-45, MKN-1)

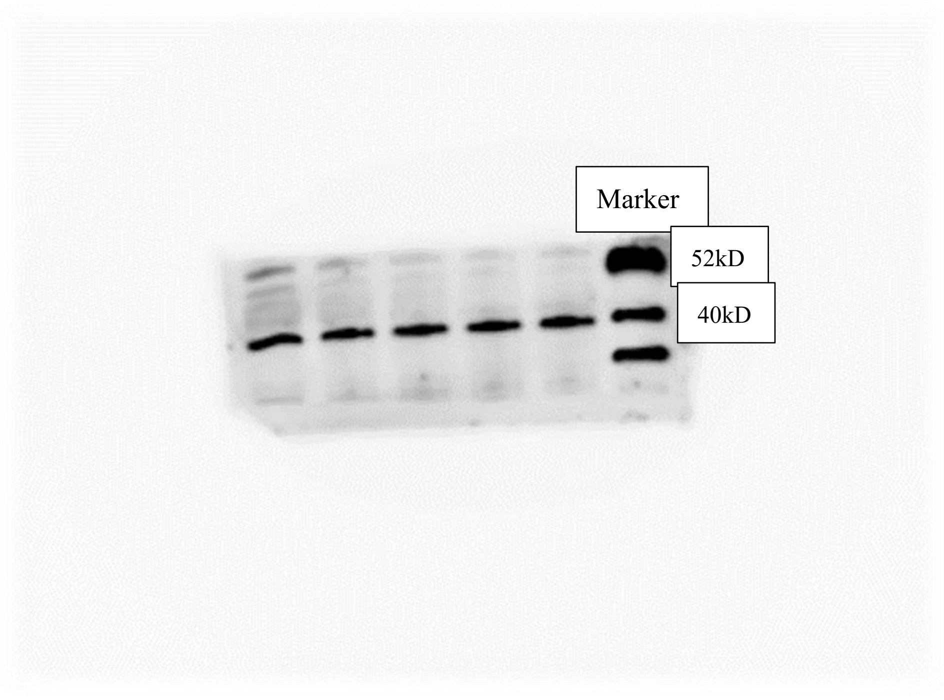

Figure 2I
FAAH (63kD)
Repeat test 1
Left to right: HGC-27 (shNC, shFAAH1, shFAAH2); MKN-1 (shNC, shFAAH1, shFAAH2)

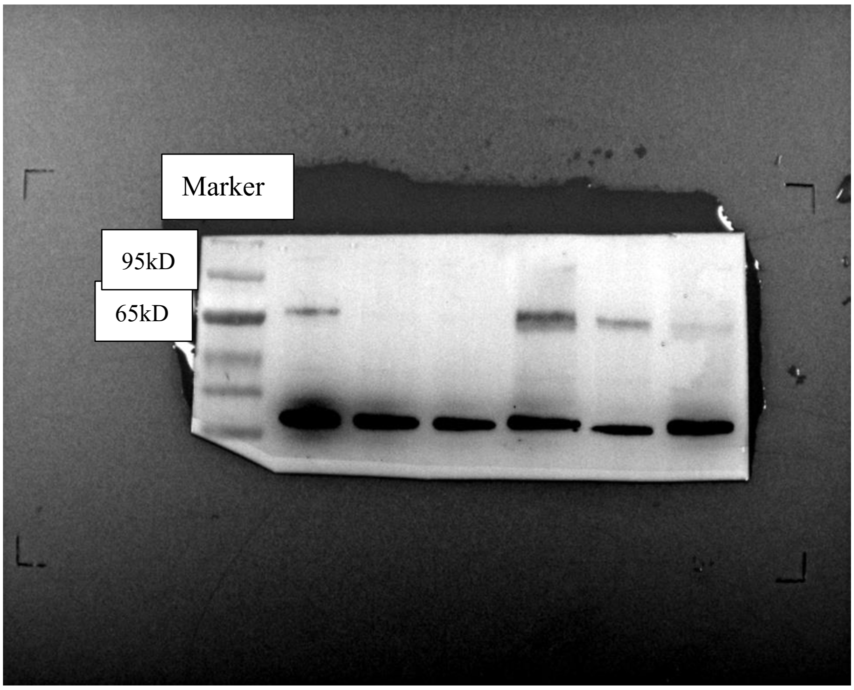

Repeat test 2
Left to right: HGC-27 (shNC, shFAAH1, shFAAH2); MKN-1 (shNC, shFAAH1, shFAAH2)

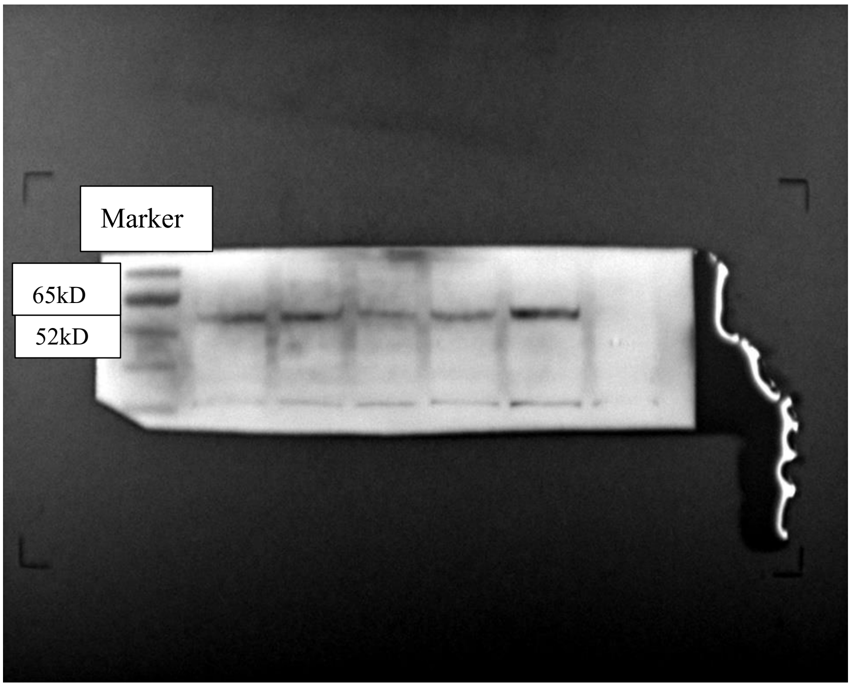

Repeat test 3

Left to right: HGC-27 (shNC, shFAAH1, shFAAH2); MKN-1 (shNC, shFAAH1, shFAAH2)

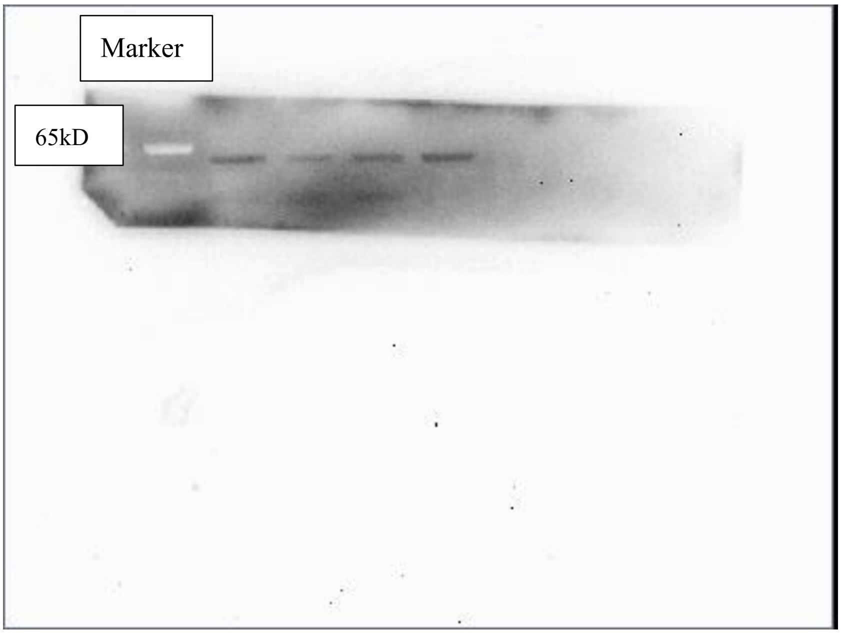

N-cadherin (125kD)

Repeat test 1

Left to right: HGC-27 (shNC, shFAAH1, shFAAH2); MKN-1 (shNC, shFAAH1, shFAAH2)

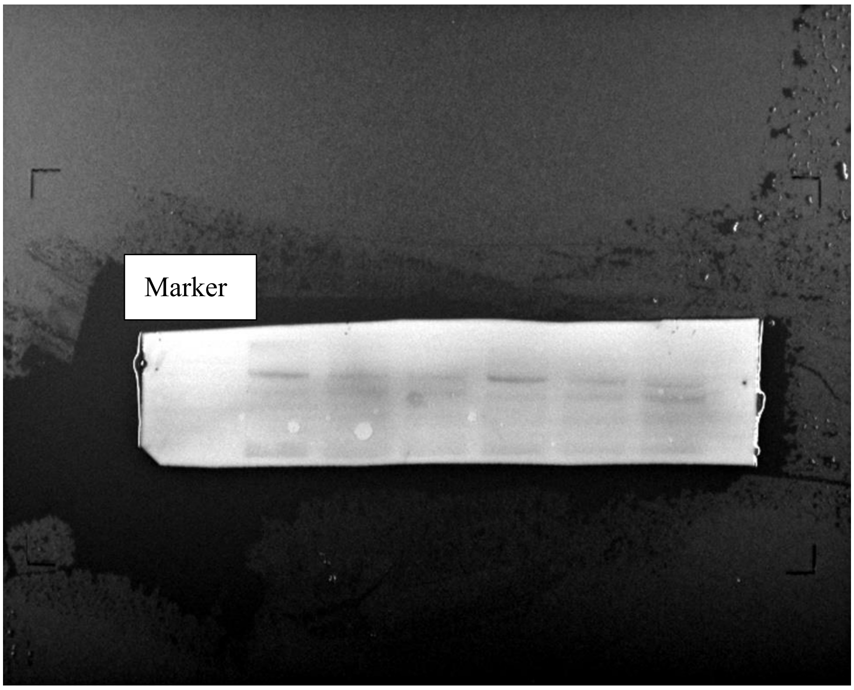

Repeat test 2

Left to right: HGC-27 (shNC, shFAAH1, shFAAH2); MKN-1 (shNC, shFAAH1, shFAAH2)

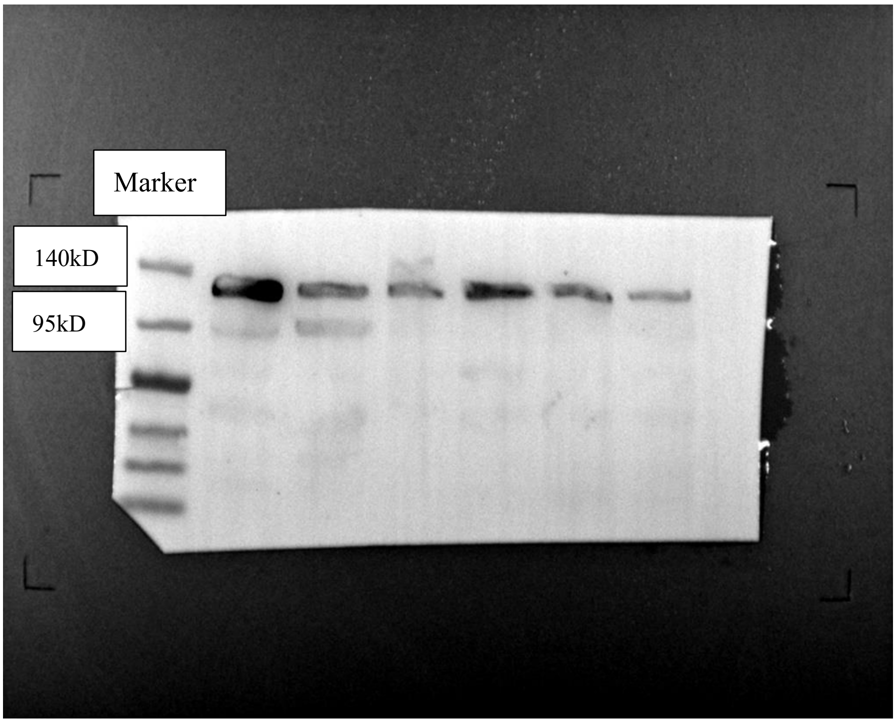

Repeat test 3

Left to right: HGC-27 (shNC, shFAAH1, shFAAH2); MKN-1 (shNC, shFAAH1, shFAAH2)

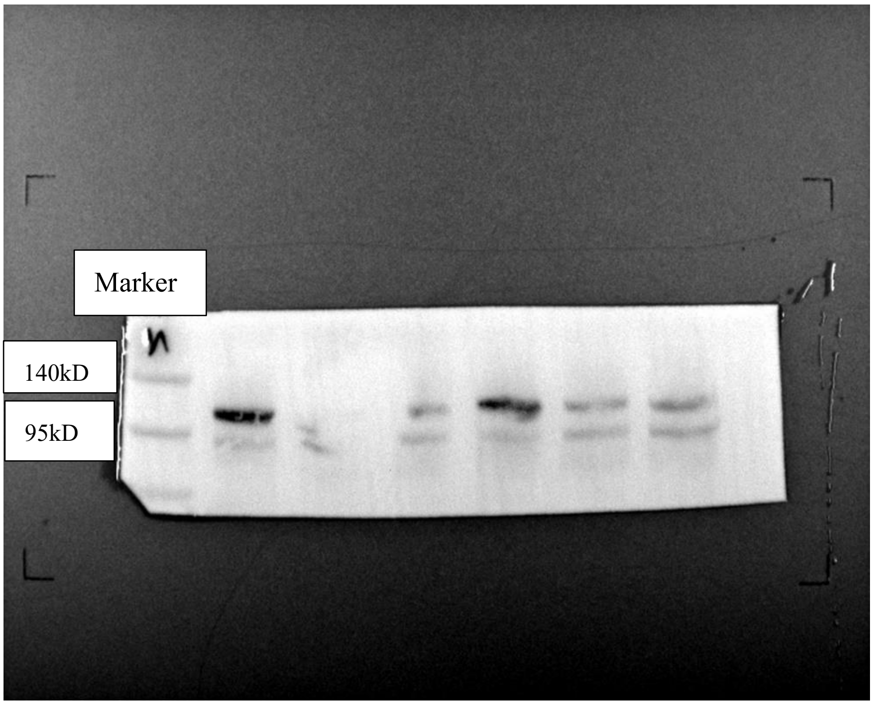

E-cadherin (97kD)

Repeat test 1

Left to right: HGC-27 (shNC, shFAAH1, shFAAH2); MKN-1 (shNC, shFAAH1, shFAAH2)

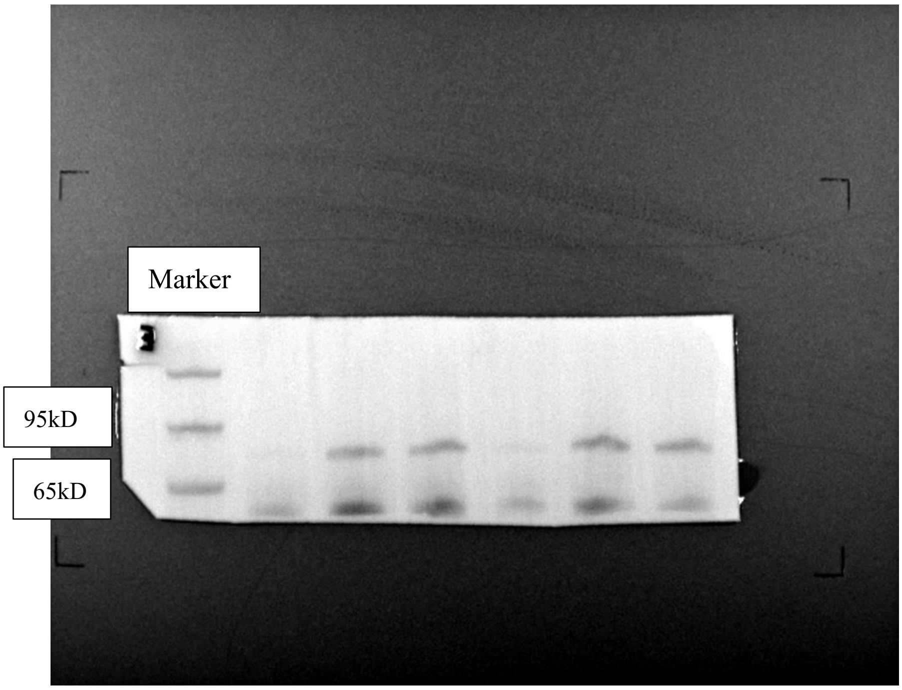

Repeat test 2

Left to right: HGC-27 (shNC, shFAAH1, shFAAH2); MKN-1 (shNC, shFAAH1, shFAAH2)

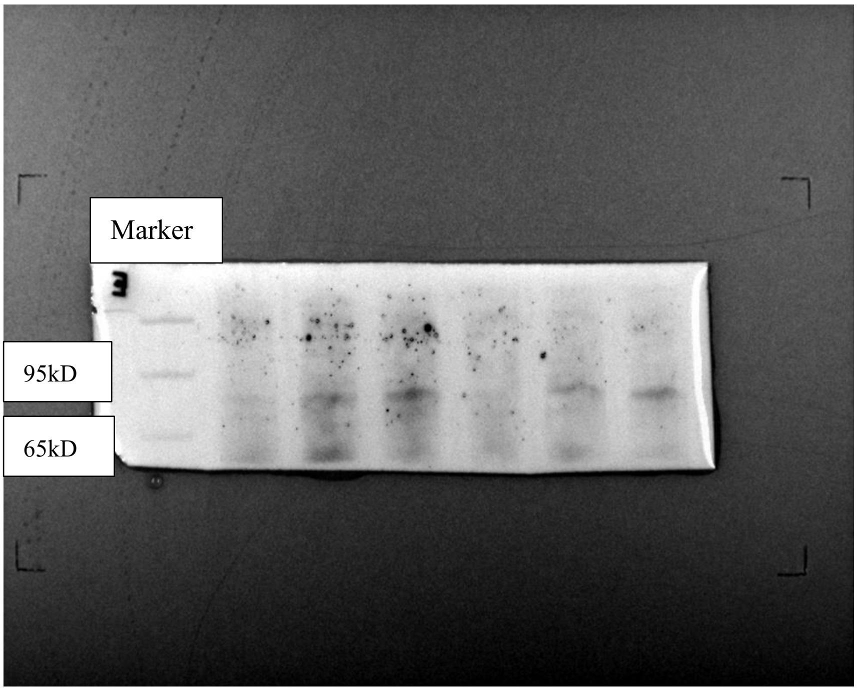

Repeat test 3

Left to right: HGC-27 (shNC, shFAAH1, shFAAH2); MKN-1 (shNC, shFAAH1, shFAAH2)

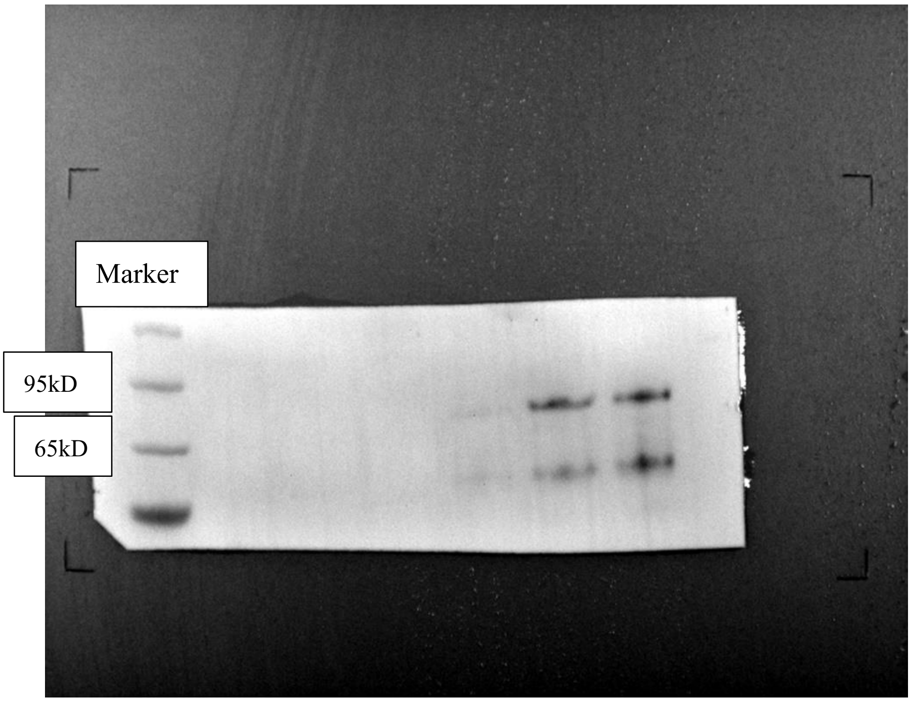

Vimenin (54kD)

Repeat test 1

Left to right: HGC-27 (shNC, shFAAH1, shFAAH2); MKN-1 (shNC, shFAAH1, shFAAH2)

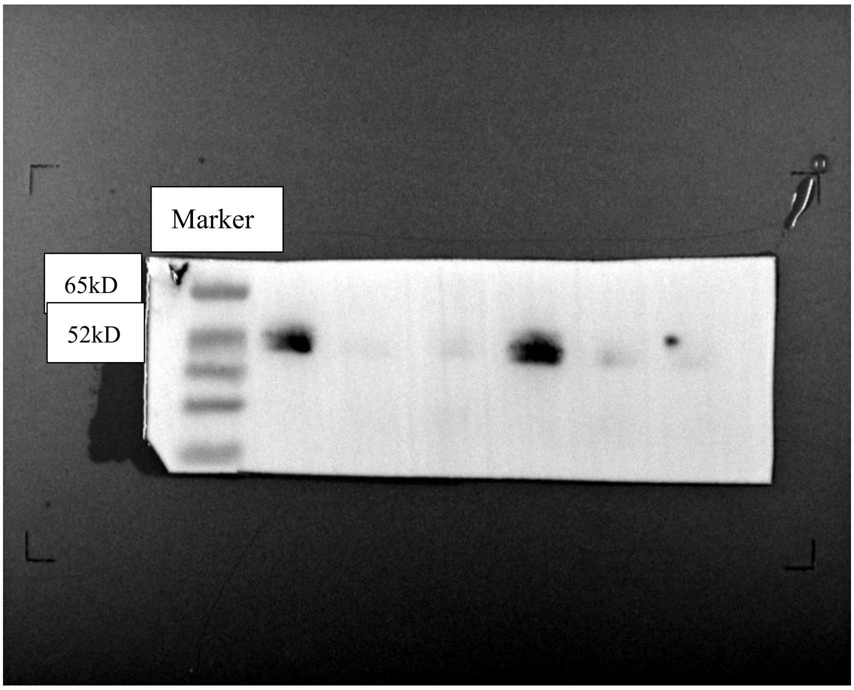

Repeat test 2
Left to right: HGC-27 (shFAAH1, shFAAH2, shNC; Carelessly mistaken the order of loading); MKN-1 (shNC, shFAAH1, shFAAH2)

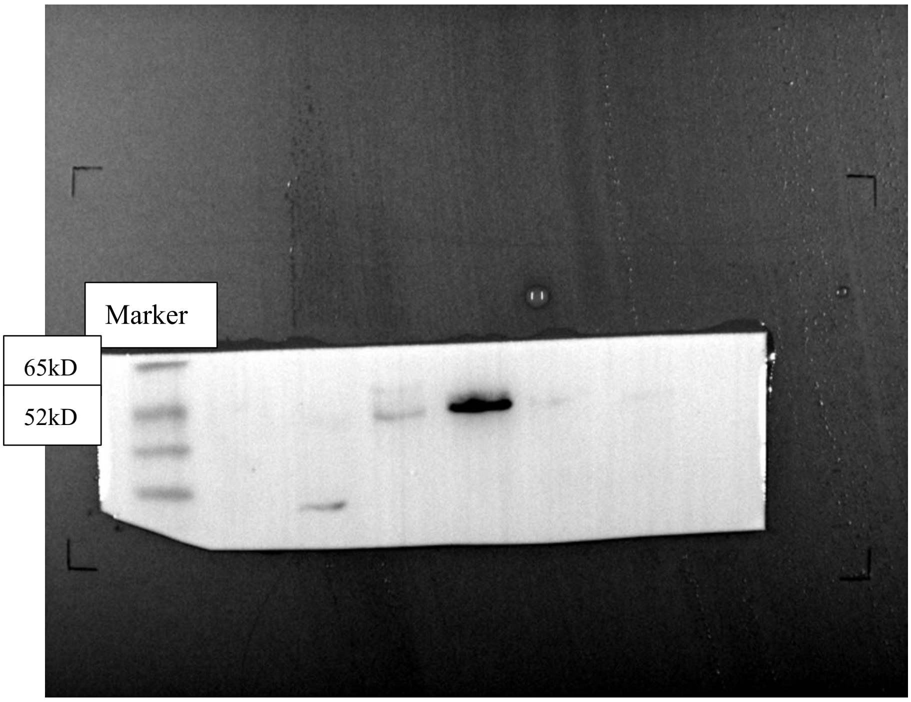

Repeat test 3
Left to right: HGC-27 (shNC, shFAAH1, shFAAH2); MKN-1 (shNC, shFAAH1, shFAAH2)

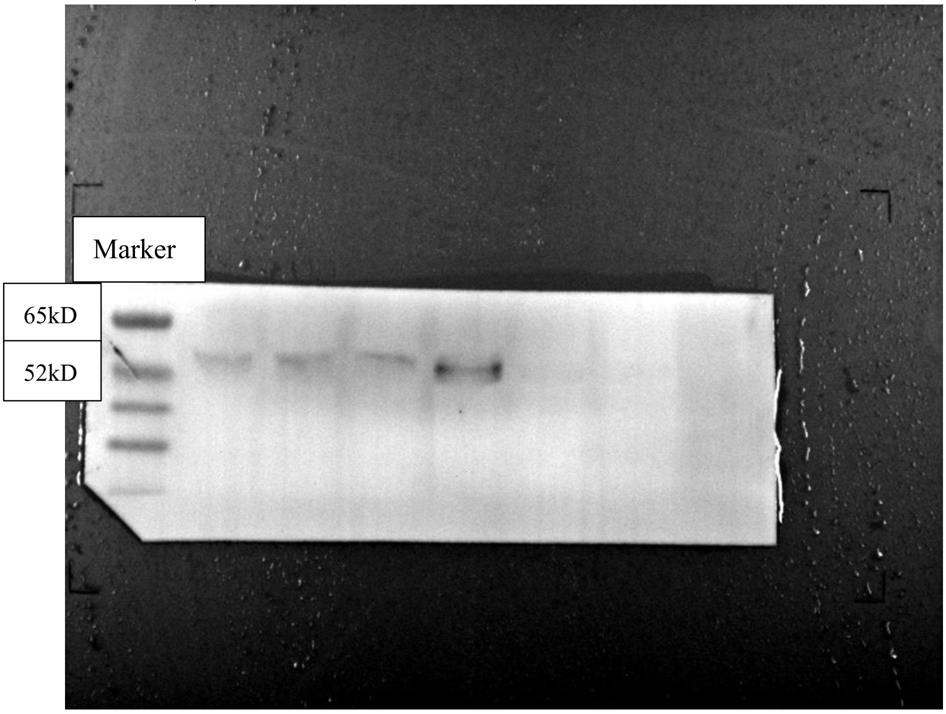

Snail/Slug (65kD)

Repeat test 1

Left to right: HGC-27 (shNC, shFAAH1, shFAAH2); MKN-1 (shNC, shFAAH1, shFAAH2)

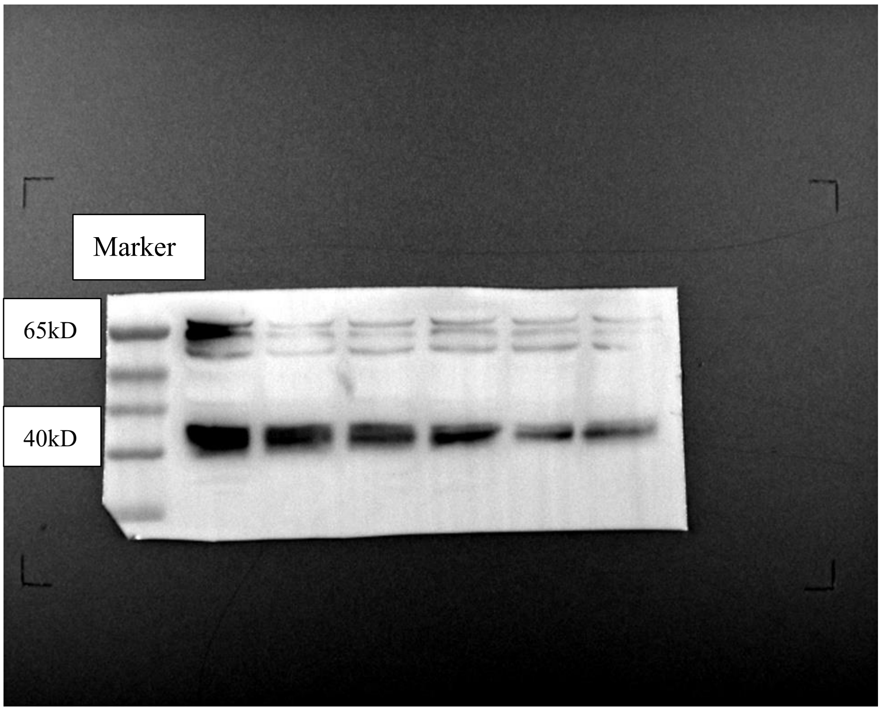

Repeat test 2

Left to right: HGC-27 (shNC, shFAAH1, shFAAH2); MKN-1 (shNC, shFAAH1, shFAAH2)

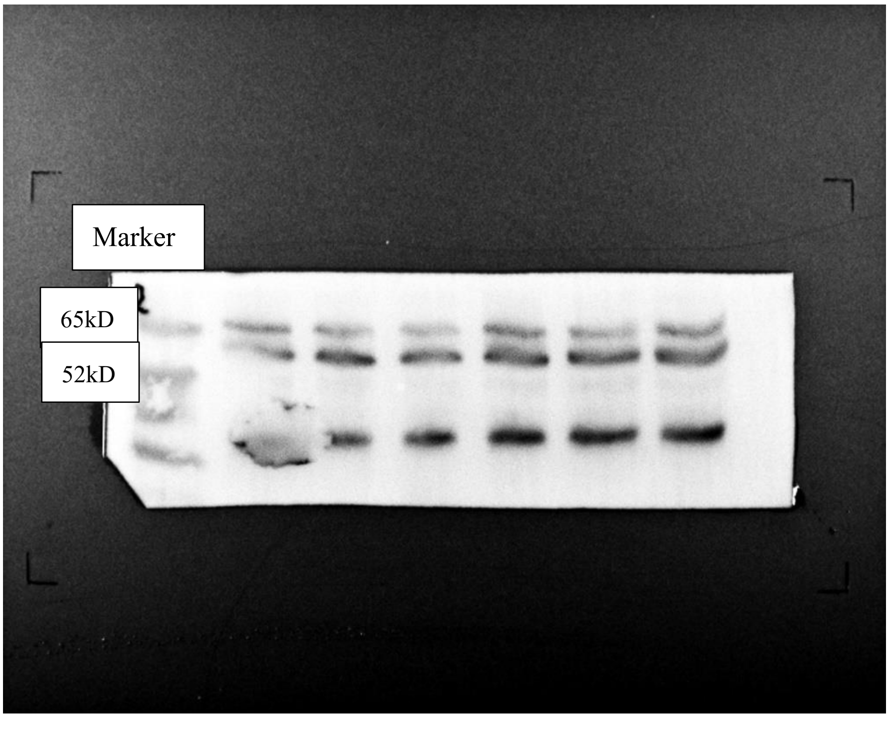

Repeat test 3

Left to right: HGC-27 (shNC, shFAAH1, shFAAH2); MKN-1 (shNC, shFAAH1, shFAAH2)

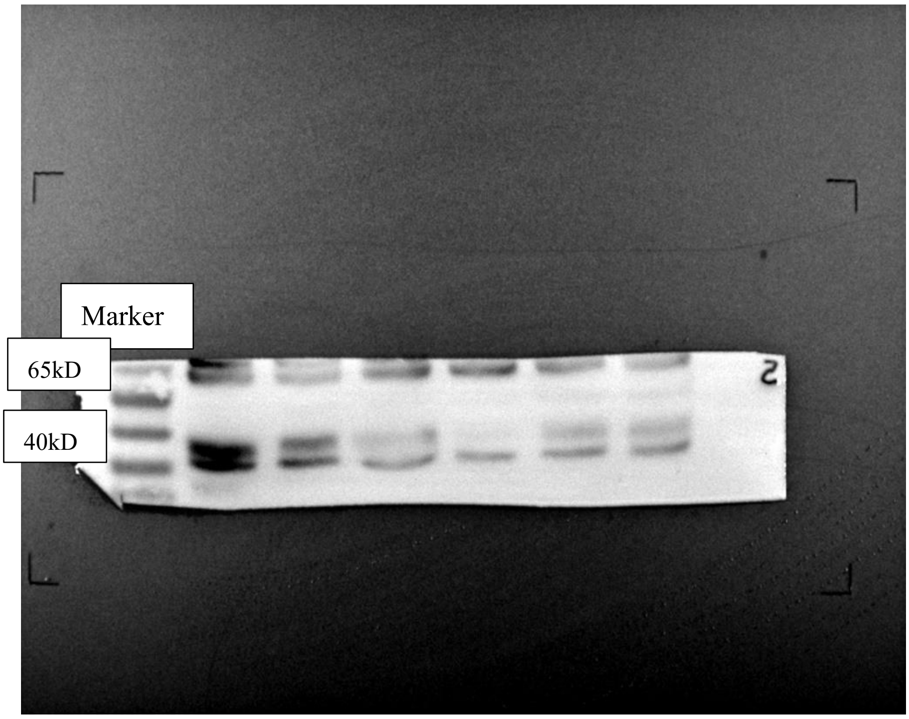

β-actin (42 kD)

Repeat test 1

Left to right: HGC-27 (shNC, shFAAH1, shFAAH2); MKN-1 (shNC, shFAAH1, shFAAH2)

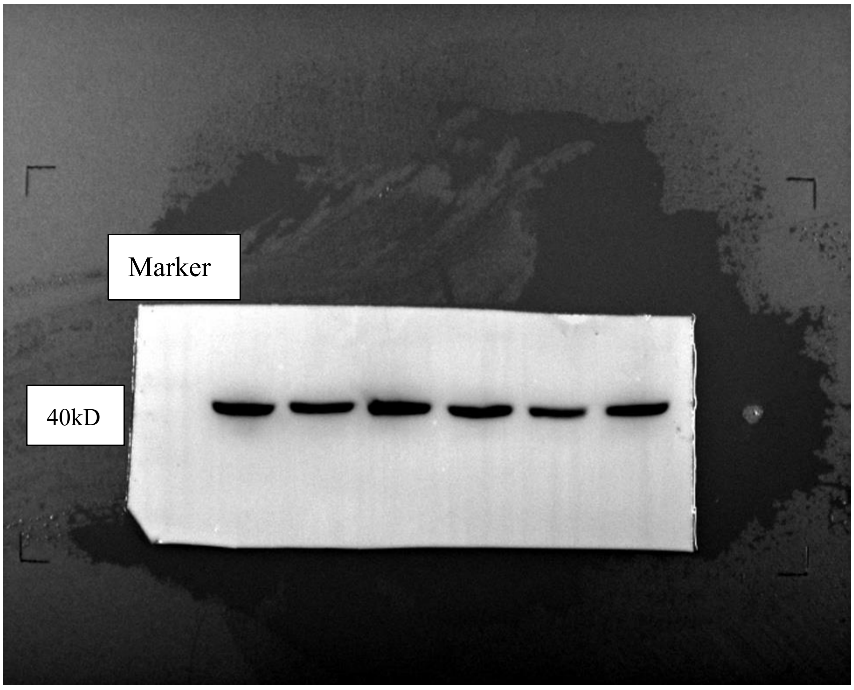

Repeat test 2

Left to right: HGC-27 (shNC, shFAAH1, shFAAH2); MKN-1 (shNC, shFAAH1, shFAAH2)

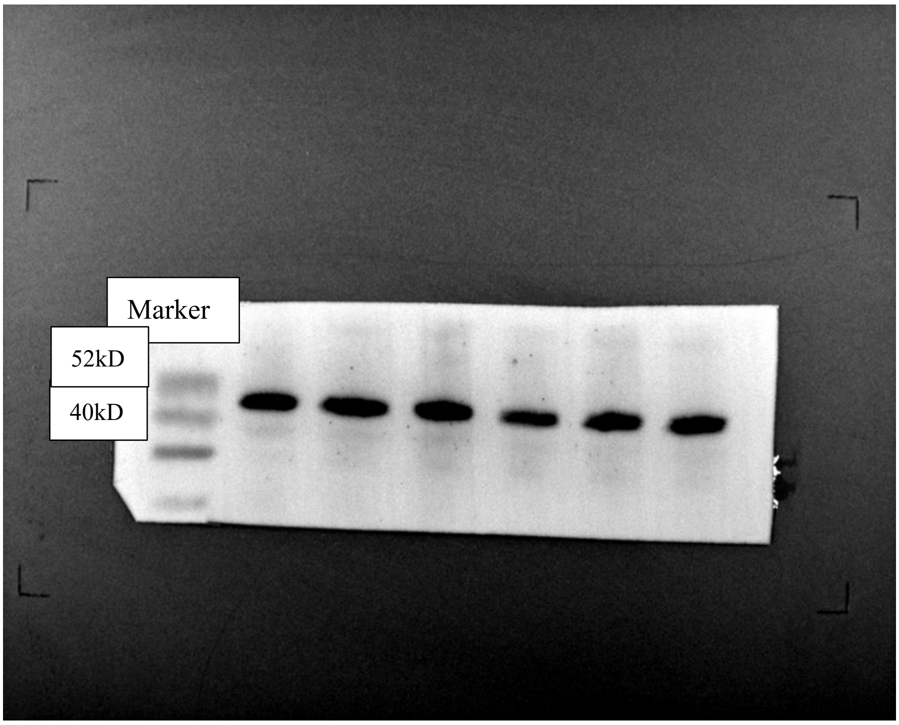

Repeat test 3

Left to right: HGC-27 (shNC, shFAAH1, shFAAH2); MKN-1 (shNC, shFAAH1, shFAAH2)

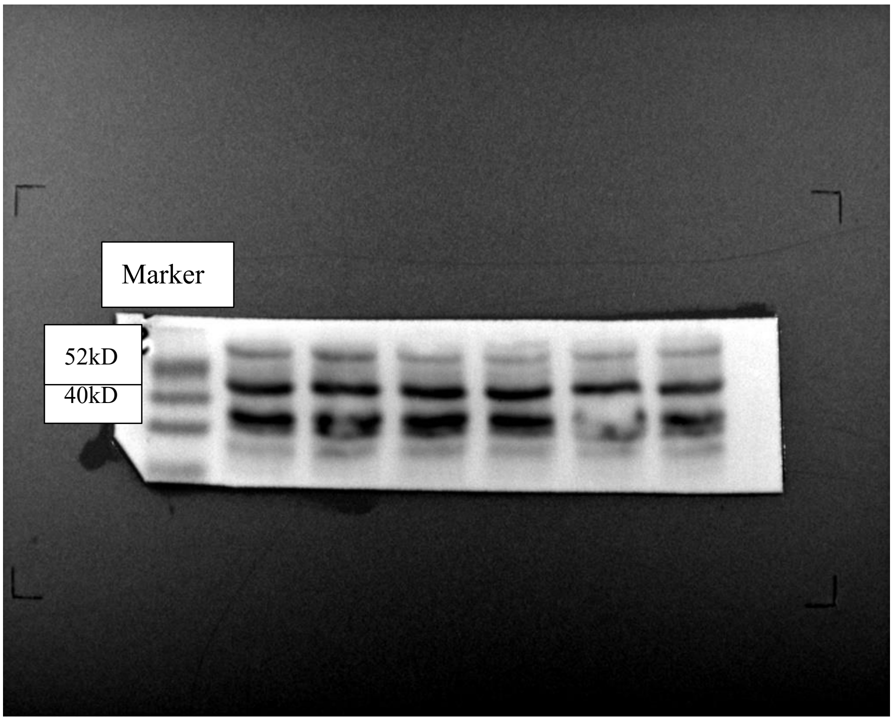

Figure 2L
Cyclin D1 (34kD)
Repeat test 1
Left to right: HGC-27 (shNC, shFAAH1, shFAAH2); MKN-1 (shNC, shFAAH1, shFAAH2)

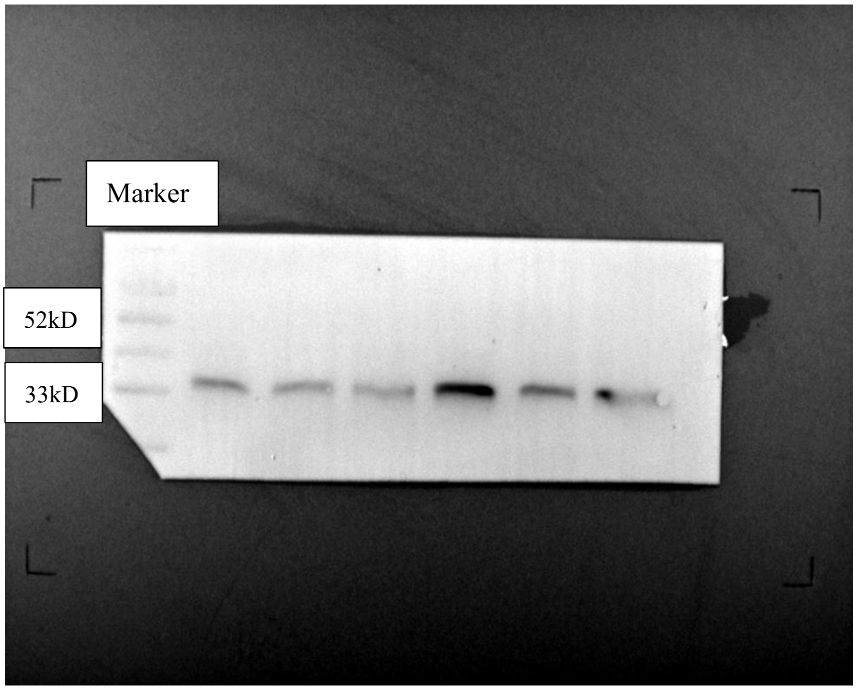

Repeat test 2
Left to right: HGC-27 (shNC, shFAAH1, shFAAH2); MKN-1 (shNC, shFAAH1, shFAAH2)

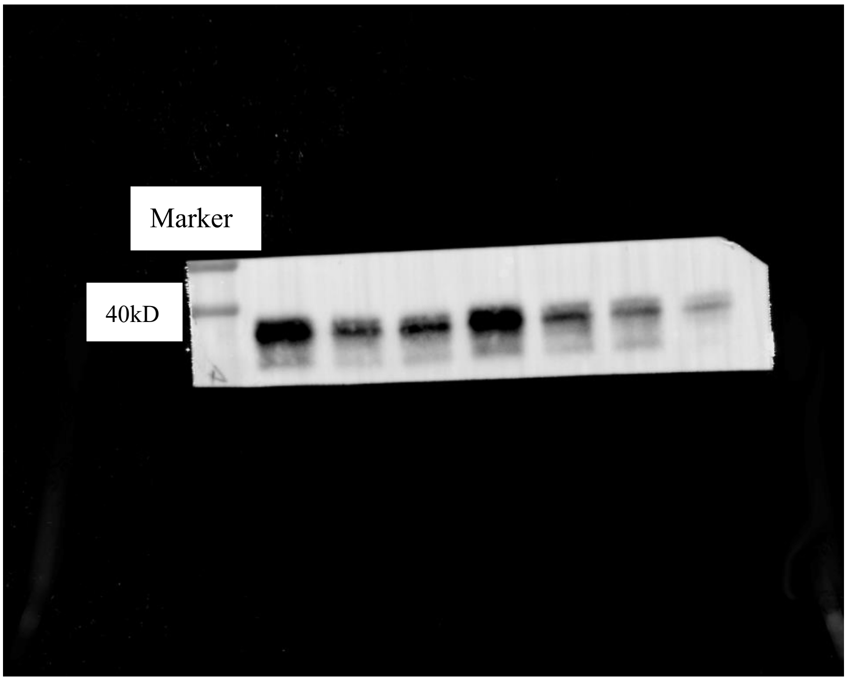

Repeat test 3

Left to right: HGC-27 (shNC, shFAAH1, shFAAH2); MKN-1 (shNC, shFAAH1, shFAAH2)

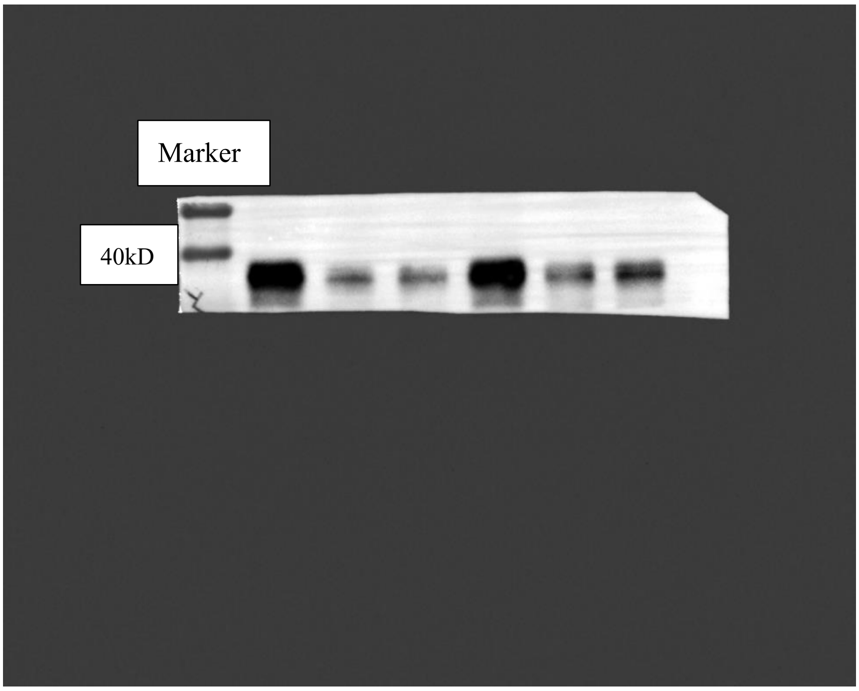

P27 (27kD)

Repeat test 1

Left to right: HGC-27 (shNC, shFAAH1, shFAAH2); MKN-1 (shNC, shFAAH1, shFAAH2)

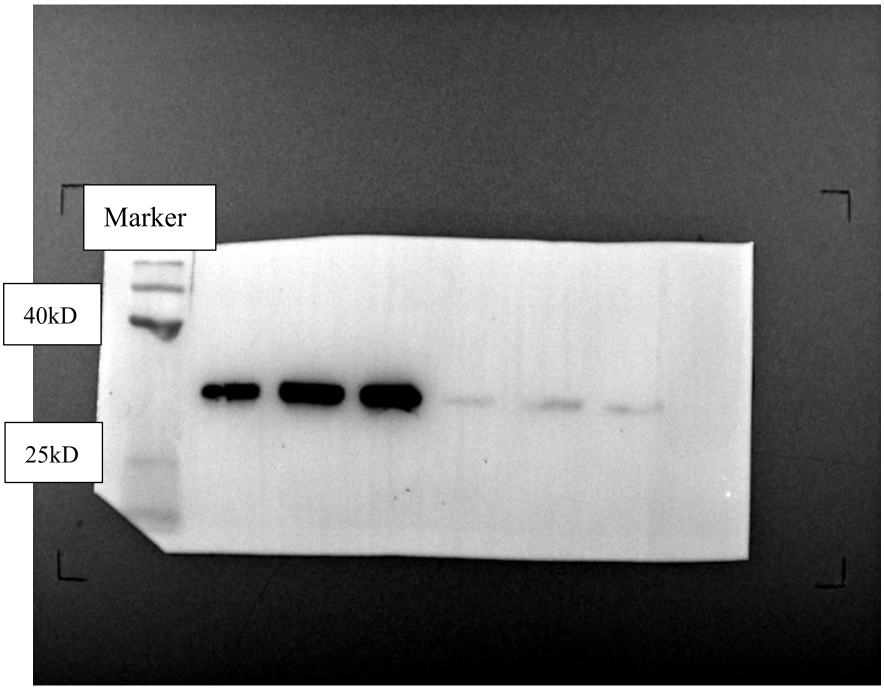

Repeat test 2

Left to right: HGC-27 (shNC, shFAAH1, shFAAH2); MKN-1 (shNC, shFAAH1, shFAAH2)

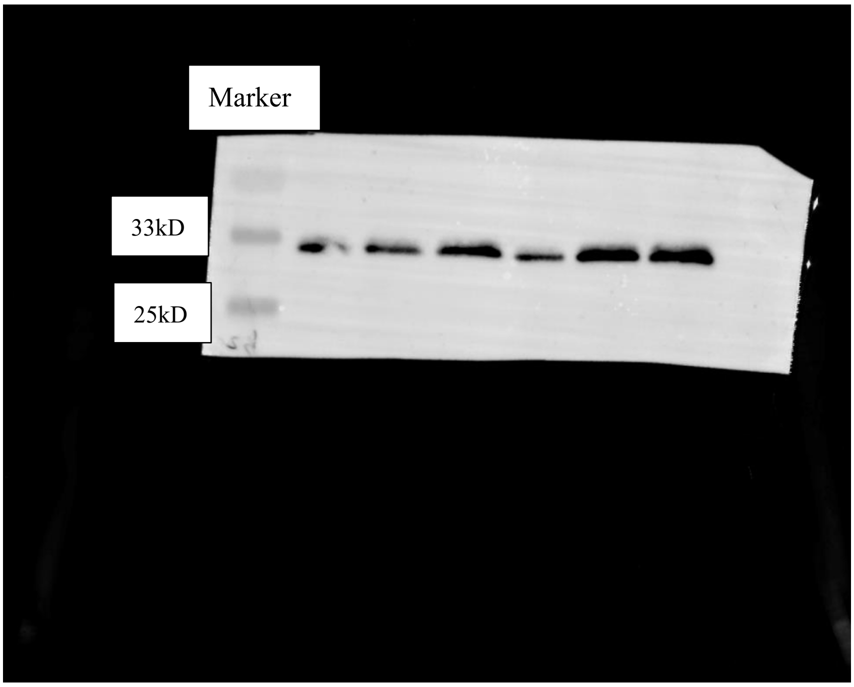

Repeat test 3

Left to right: HGC-27 (shNC, shFAAH1, shFAAH2); MKN-1 (shNC, shFAAH1, shFAAH2)

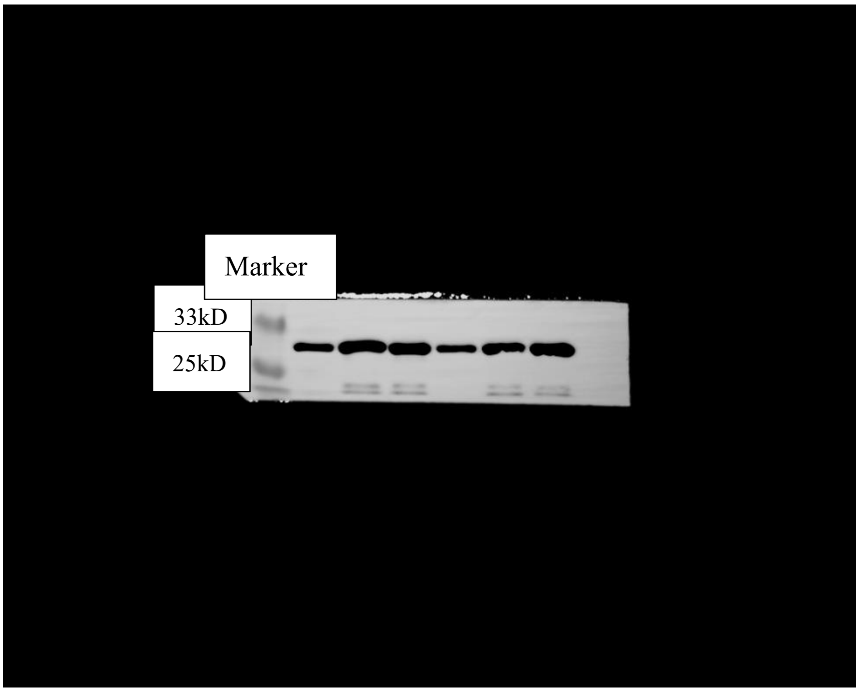

Bcl2 (26kD)

Repeat test 1

Left to right: HGC-27 (shNC, shFAAH1, shFAAH2); MKN-1 (shNC, shFAAH1, shFAAH2)

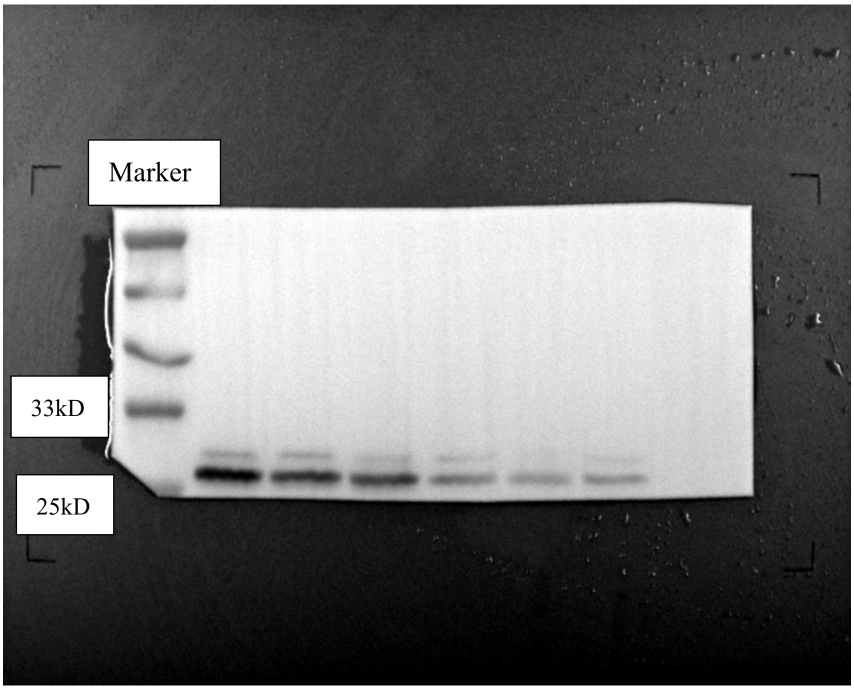

Repeat test 2

Left to right: HGC-27 (shNC, shFAAH1, shFAAH2); MKN-1 (shNC, shFAAH1, shFAAH2)

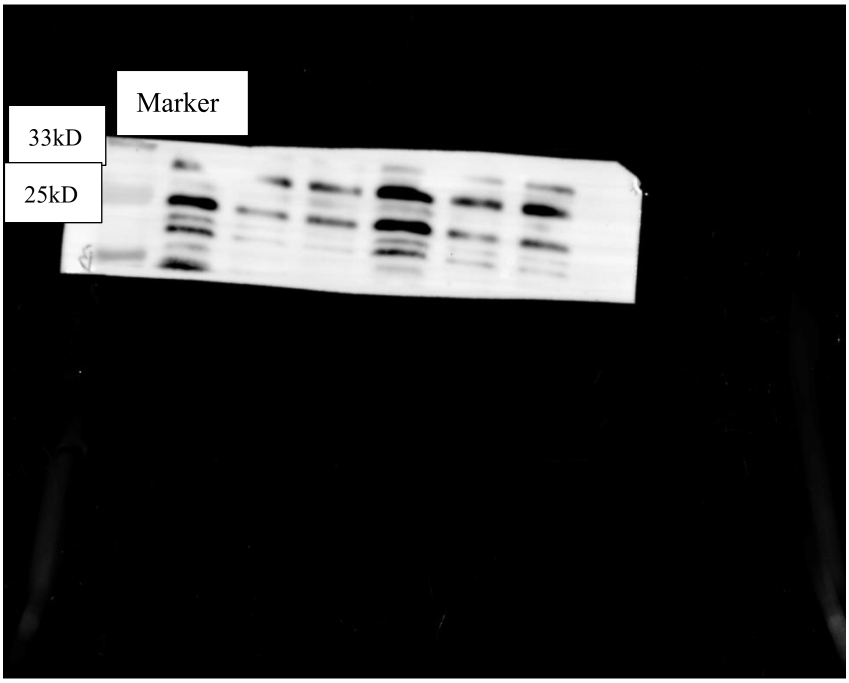

Repeat test 3

Left to right: HGC-27 (shNC, shFAAH1, shFAAH2); MKN-1 (shNC, shFAAH1, shFAAH2)

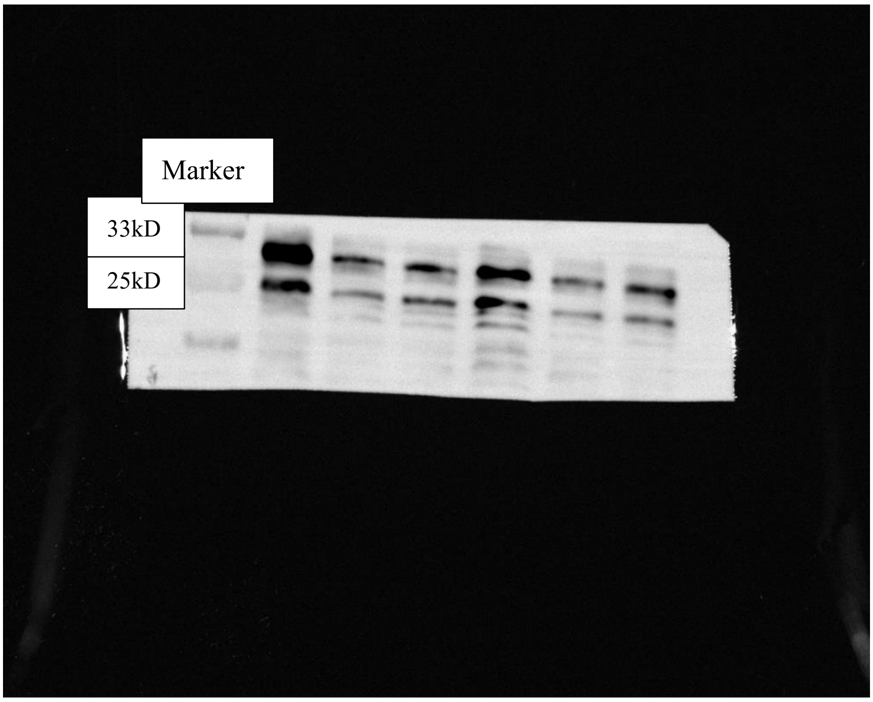

Bax (21kD)

Repeat test 1

Left to right: HGC-27 (shNC, shFAAH1, shFAAH2); MKN-1 (shNC, shFAAH1, shFAAH2)

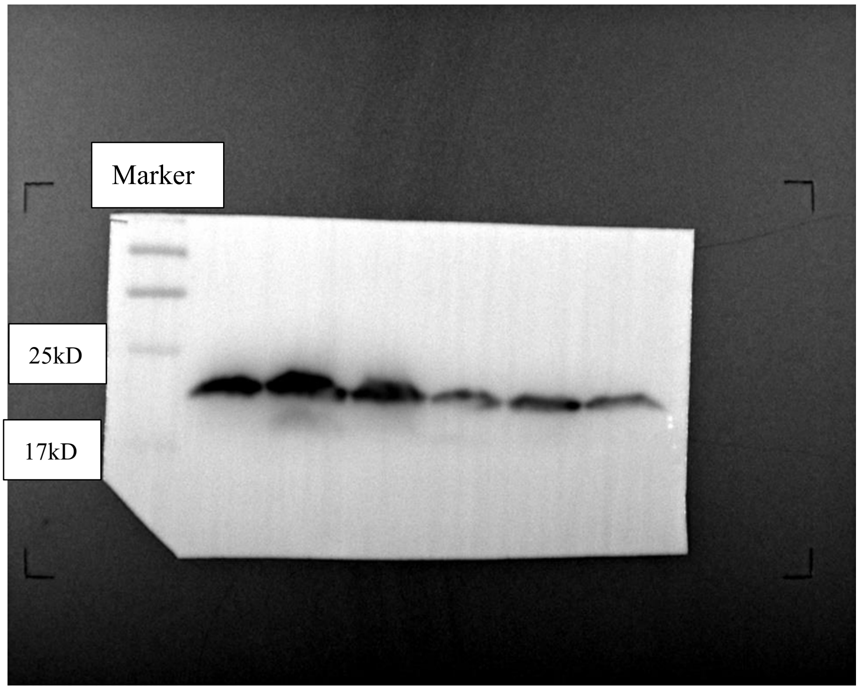

Repeat test 2

Left to right: HGC-27 (shNC, shFAAH1, shFAAH2); MKN-1 (shNC, shFAAH1, shFAAH2)

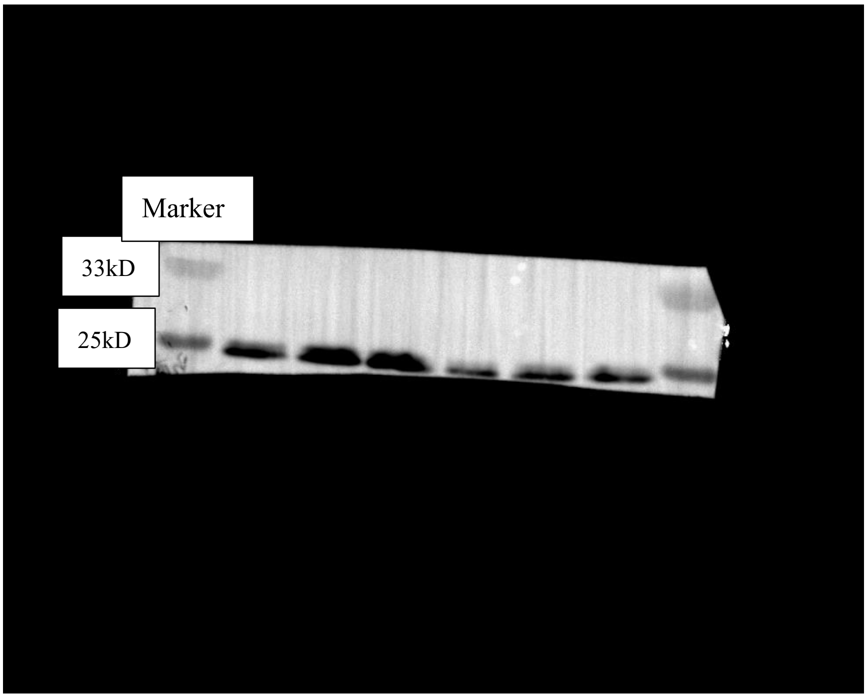

Repeat test 3

Left to right: HGC-27 (shNC, shFAAH1, shFAAH2); MKN-1 (shNC, shFAAH1, shFAAH2)

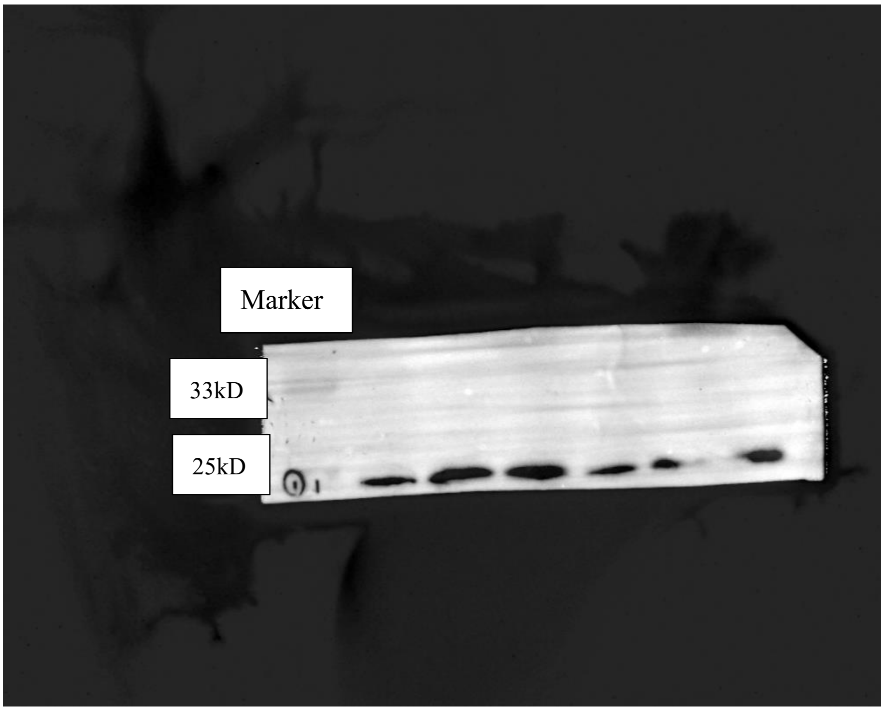

β-actin (42kD)

Repeat test 1

Left to right: HGC-27 (shNC, shFAAH1, shFAAH2); MKN-1 (shNC, shFAAH1, shFAAH2)

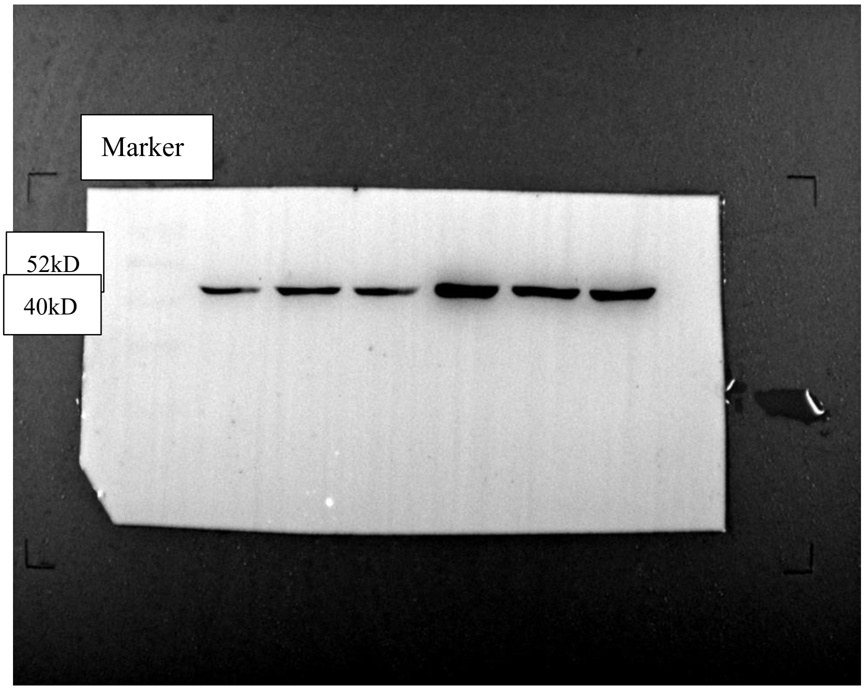

Repeat test 2

Left to right: HGC-27 (shNC, shFAAH1, shFAAH2); MKN-1 (shNC, shFAAH1, shFAAH2)

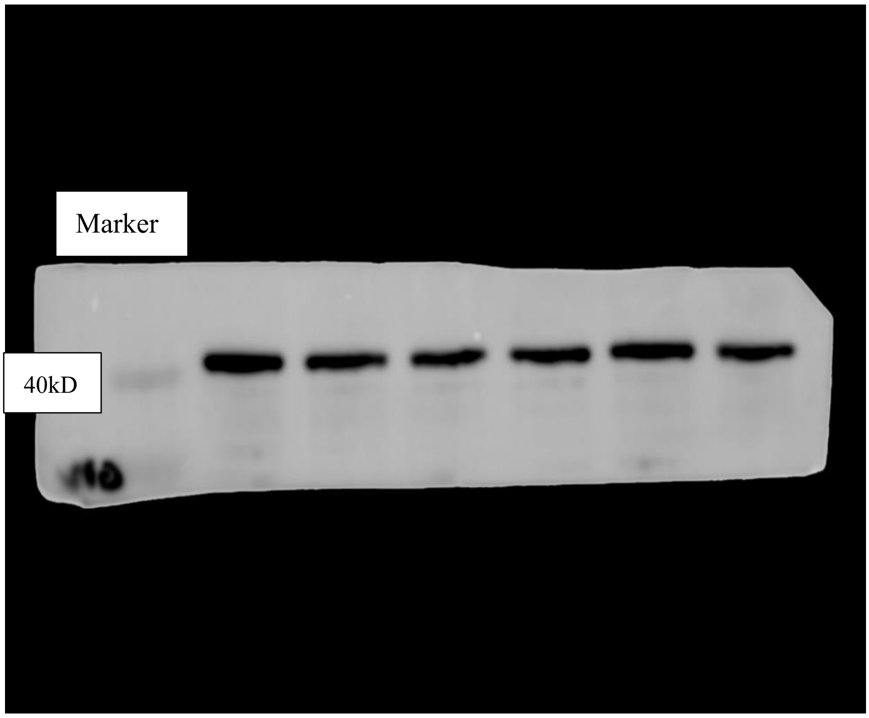

Repeat test 3

Left to right: HGC-27 (shNC, shFAAH1, shFAAH2); MKN-1 (shNC, shFAAH1, shFAAH2)

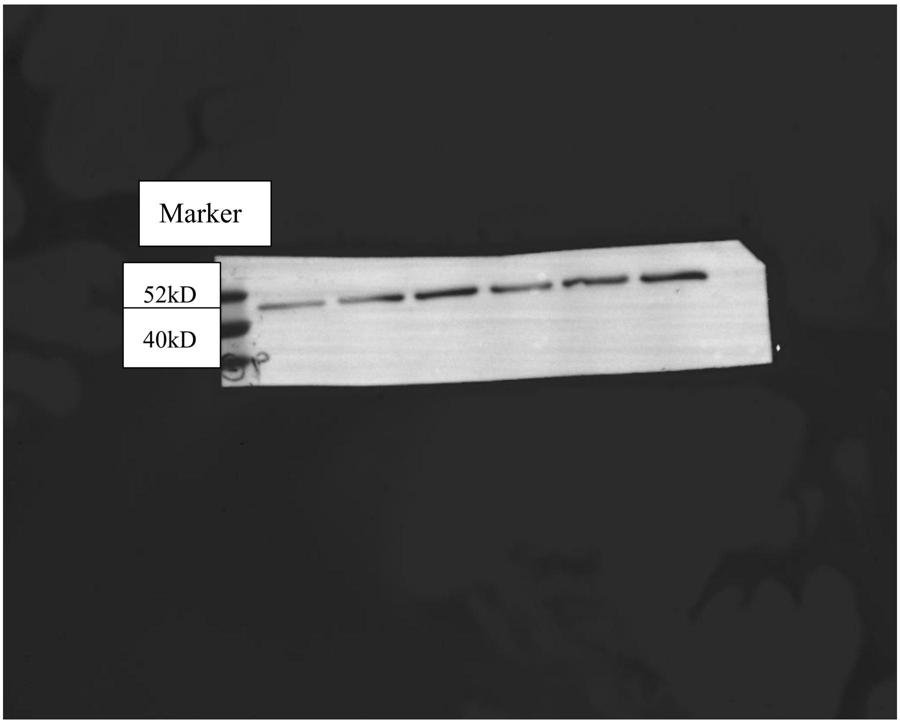

Figure 3I

FAAH (63kD)

Left to right: Experimental Group (Mock, LV-nc, LV-shFAAH); Repetition Group (Mock, LV-nc, LV-shFAAH)

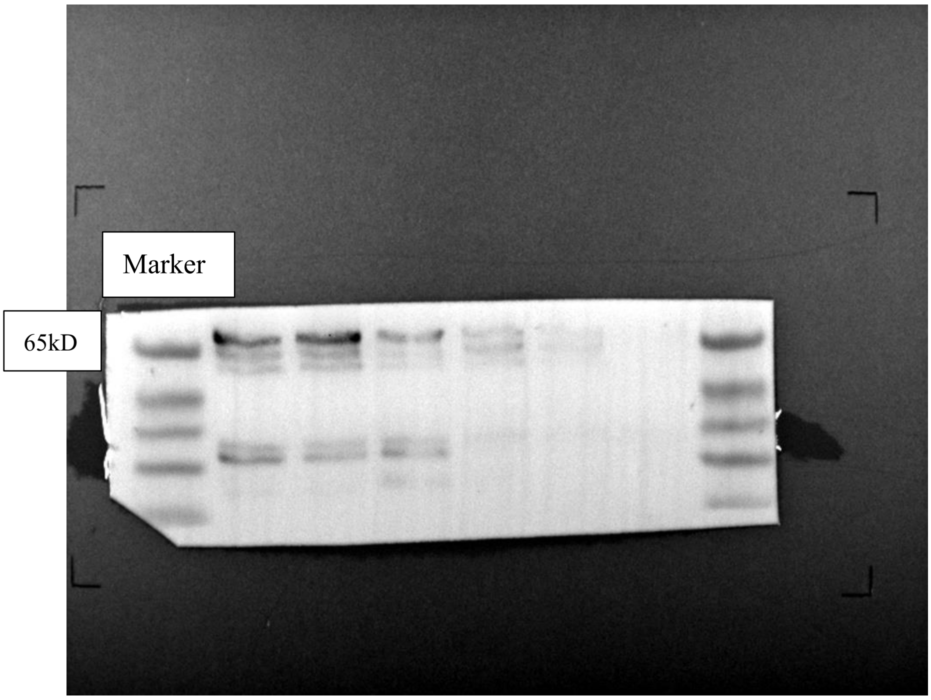

N-cadherin (125kD)

Left to right: Experimental Group (Mock, LV-nc, LV-shFAAH); Repetition Group (Mock, LV-nc, LV-shFAAH)

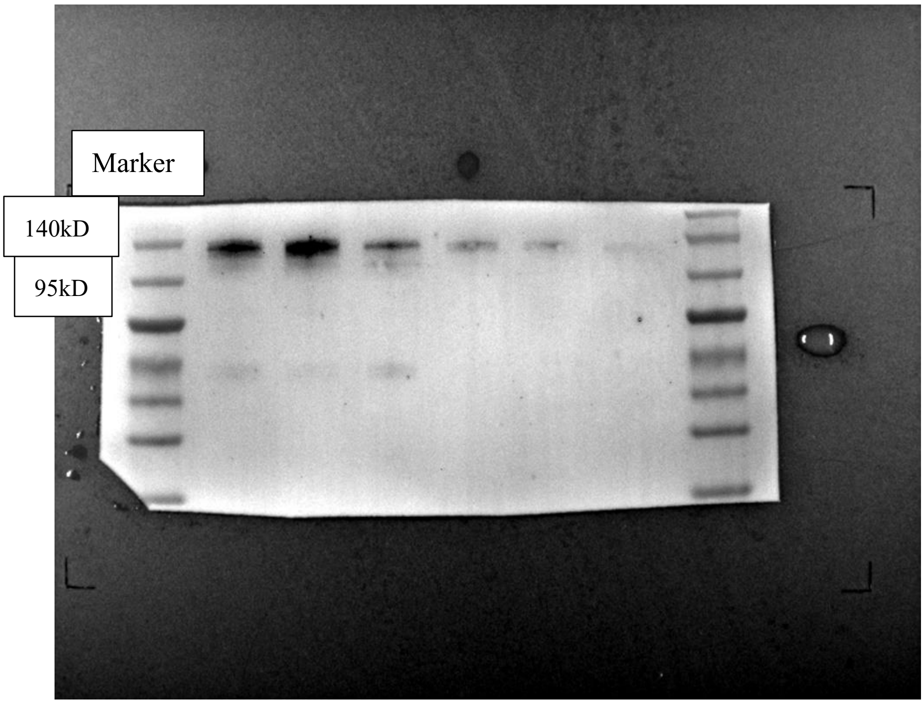

E-cadherin (97kD)

Left to right: Experimental Group (Mock, LV-nc, LV-shFAAH); Repetition Group (Mock, LV-nc, LV-shFAAH)

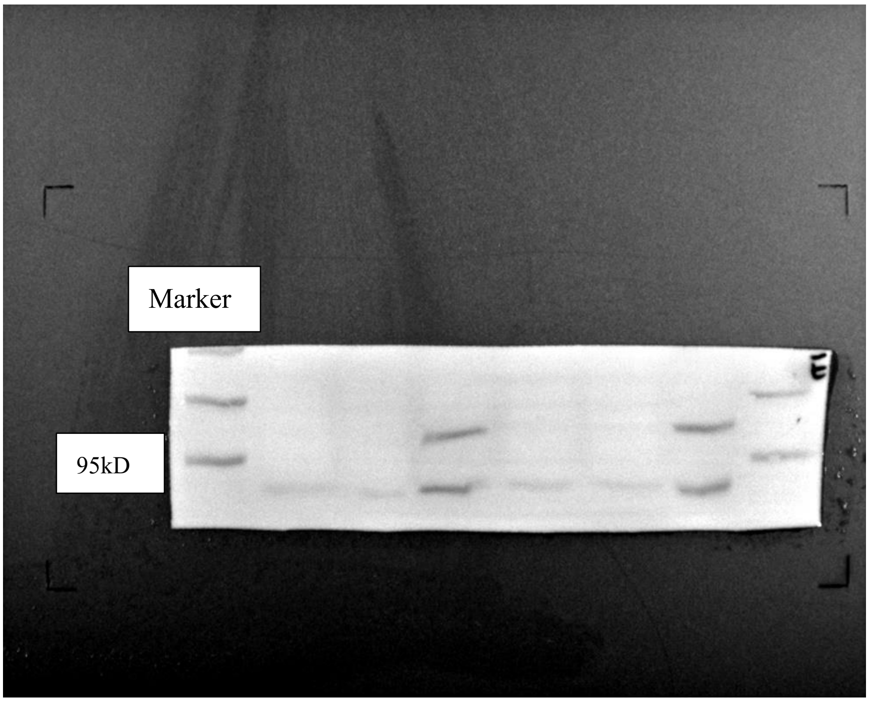

Vimenin (54kD)

Left to right: Experimental Group (Mock, LV-nc, LV-shFAAH); Repetition Group (Mock, LV-nc, LV-shFAAH)

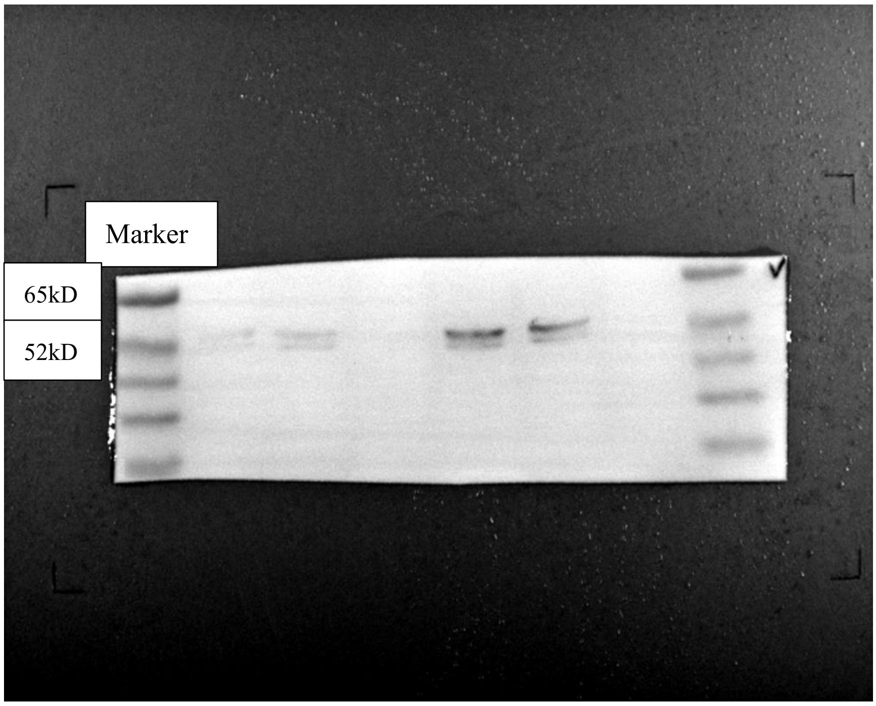

Snail/Slug (65kD)

Left to right: Experimental Group (Mock, LV-nc, LV-shFAAH); Repetition Group (Mock, LV-nc, LV-shFAAH)

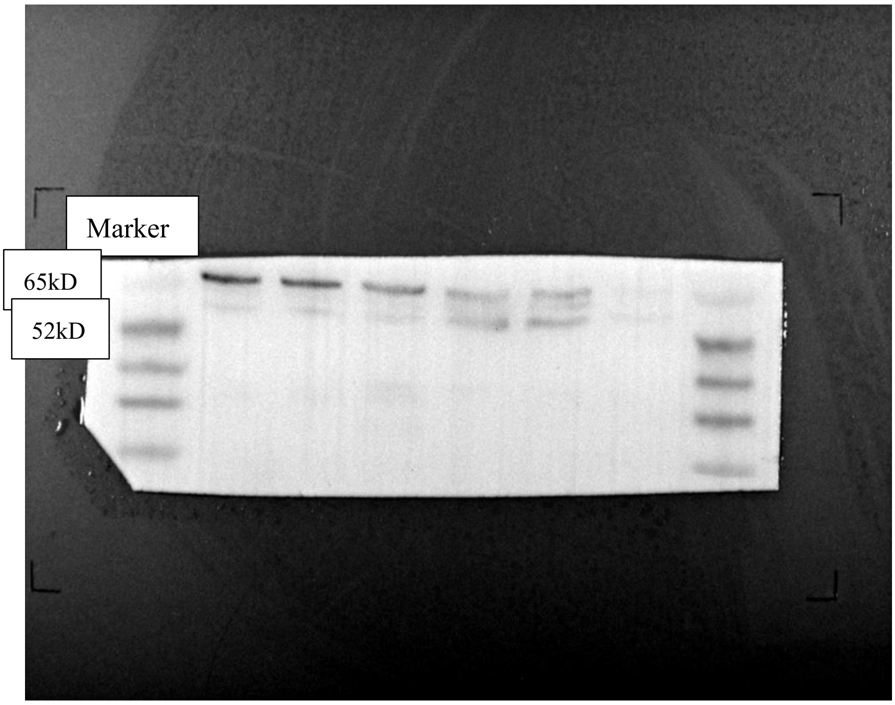

β-actin (42 kD)

Left to right: Experimental Group (Mock, LV-nc, LV-shFAAH); Repetition Group (Mock, LV-nc, LV-shFAAH)

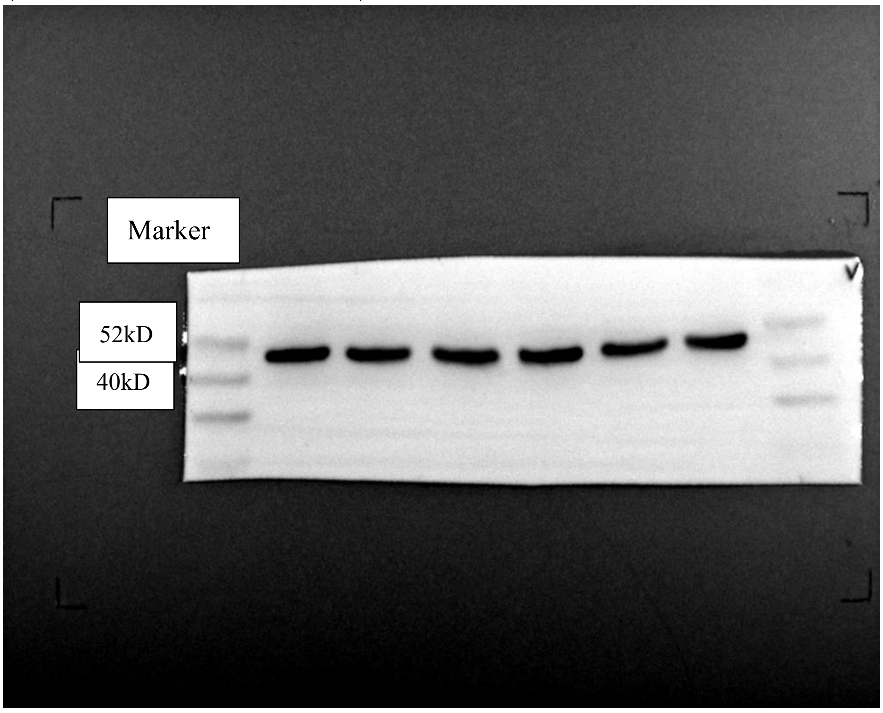

Figure 5I

FAAH (63kD)

Repeat test 1

Left to right: HGC-27 (shNC + inhibitor-NC, shFAAH + inhibitor-NC, shFAAH + inhibitor-1275), MKN-1 (pcDNA + mimic-NC, pcFAAH + mimic-NC, pcFAAH + mimic-1275)

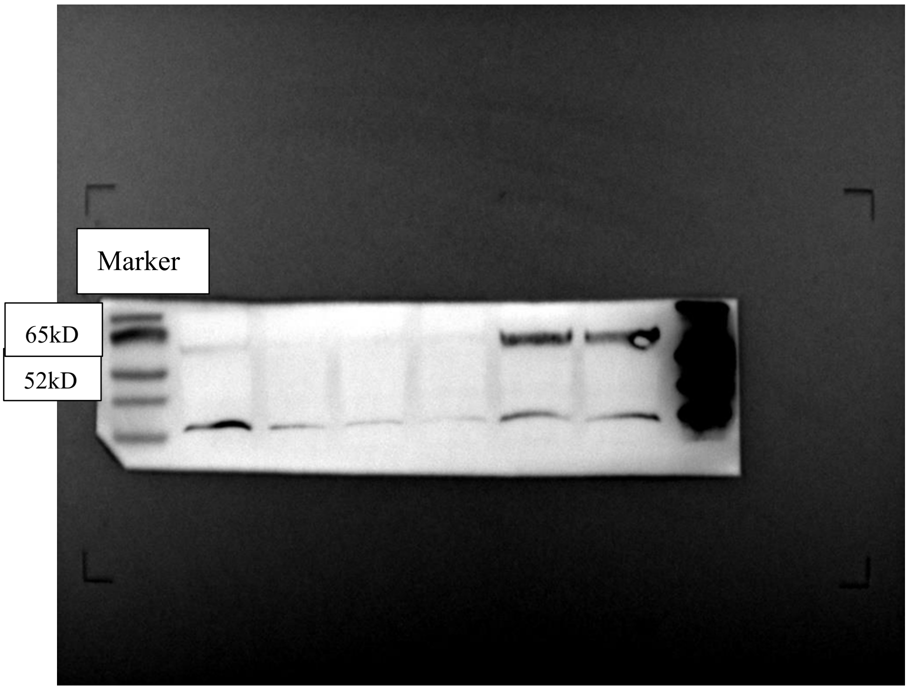

Repeat test 2

Left to right: HGC-27 (shNC + inhibitor-NC, shFAAH + inhibitor-NC, shFAAH + inhibitor-1275), MKN-1 (pcDNA + mimic-NC, pcFAAH + mimic-NC, pcFAAH + mimic-1275)

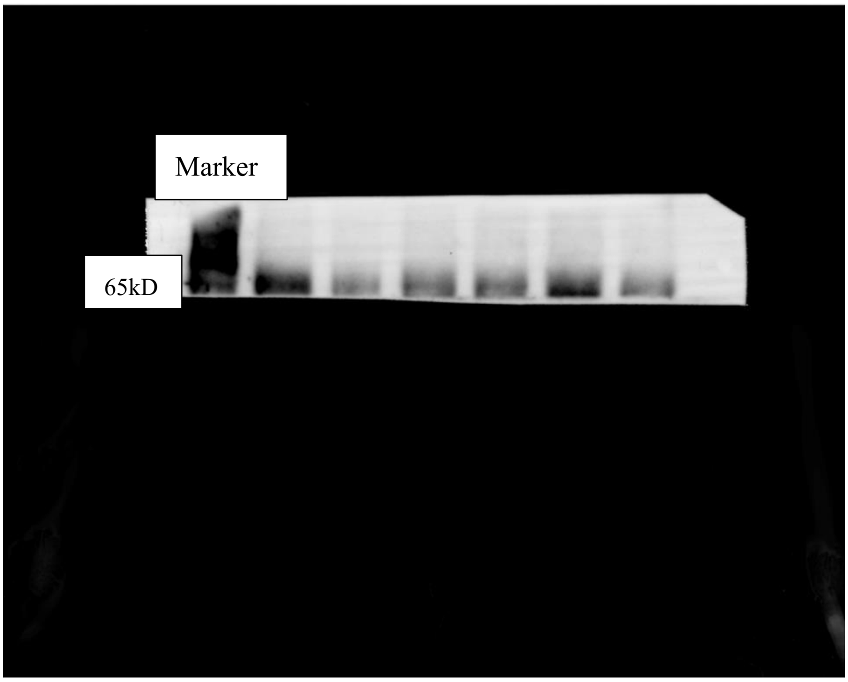

Repeat test 3

Left to right: HGC-27 (shNC + inhibitor-NC, shFAAH + inhibitor-NC, shFAAH + inhibitor-1275), MKN-1 (pcDNA + mimic-NC, pcFAAH + mimic-NC, pcFAAH + mimic-1275)

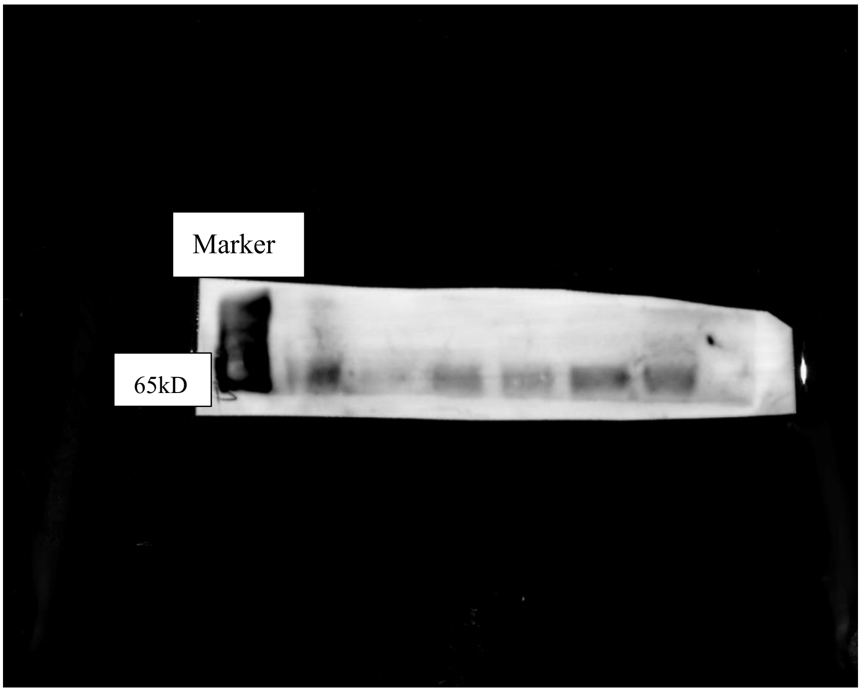

Cyclin D1 (36kD)

Repeat test 1

Left to right: HGC-27 (shNC + inhibitor-NC, shFAAH + inhibitor-NC, shFAAH + inhibitor-1275), MKN-1 (pcDNA + mimic-NC, pcFAAH + mimic-NC, pcFAAH + mimic-1275)

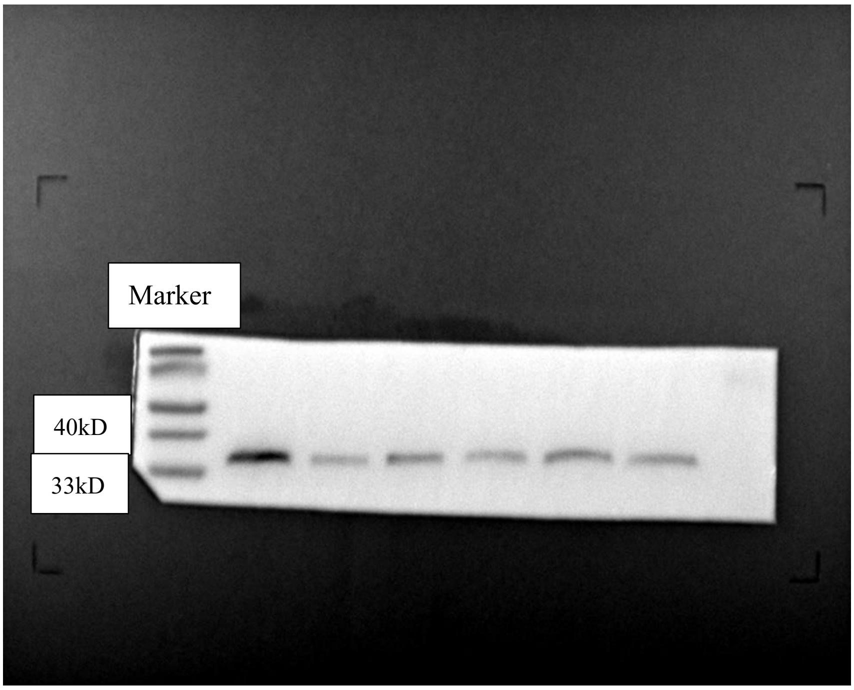

Repeat test 2

Left to right: HGC-27 (shNC + inhibitor-NC, shFAAH + inhibitor-NC, shFAAH + inhibitor-1275), MKN-1 (pcDNA + mimic-NC, pcFAAH + mimic-NC, pcFAAH + mimic-1275)

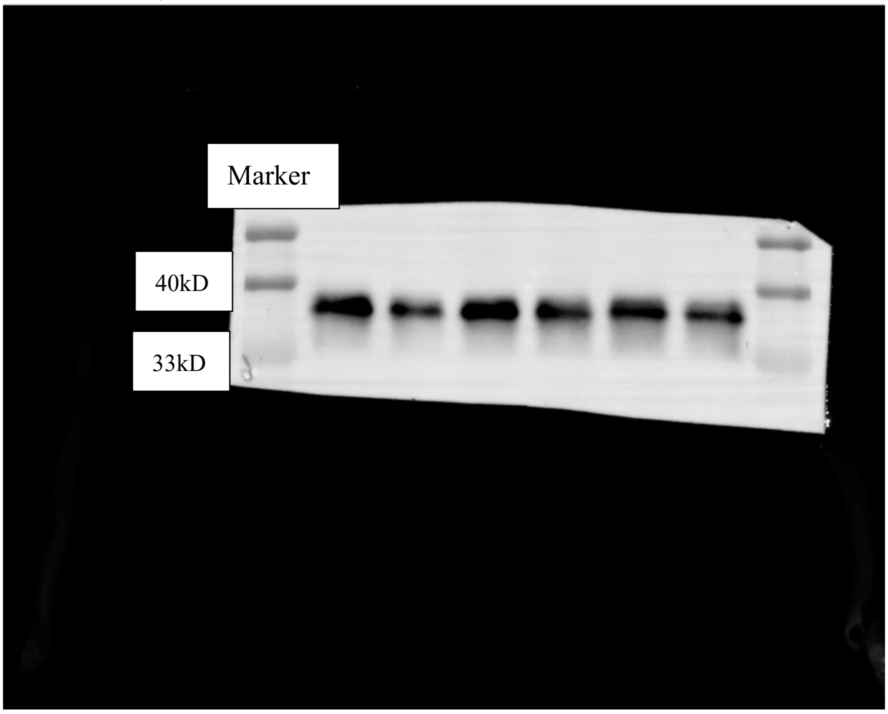

Repeat test 3

Left to right: HGC-27 (shNC + inhibitor-NC, shFAAH + inhibitor-NC, shFAAH + inhibitor-1275), MKN-1 (pcDNA + mimic-NC, pcFAAH + mimic-NC, pcFAAH + mimic-1275)

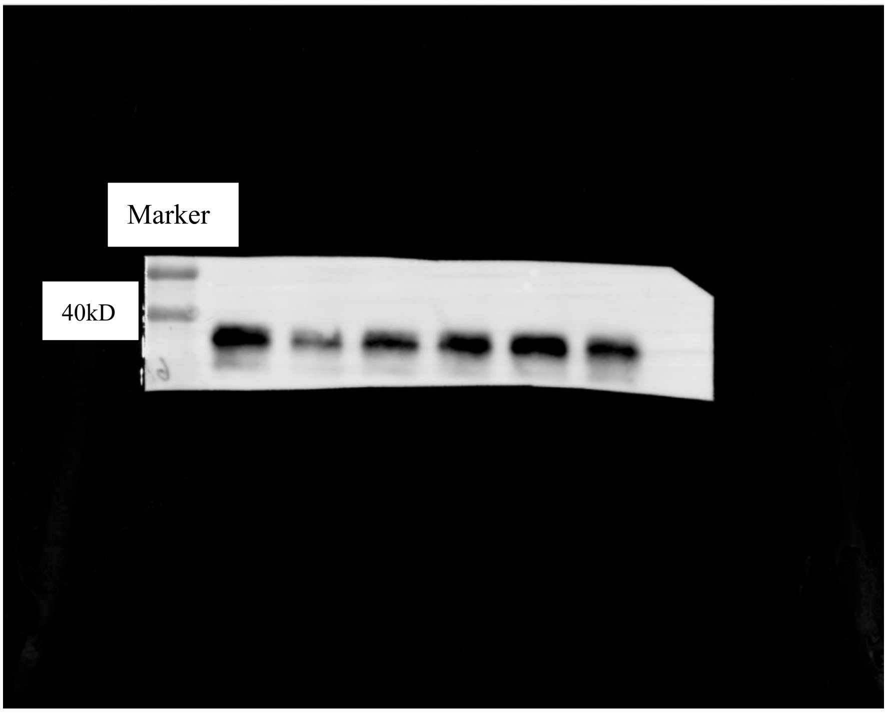

P27 (27kD)

Repeat test 1

Left to right: HGC-27 (shNC + inhibitor-NC, shFAAH + inhibitor-NC, shFAAH + inhibitor-1275), MKN-1 (pcDNA + mimic-NC, pcFAAH + mimic-NC, pcFAAH + mimic-1275)

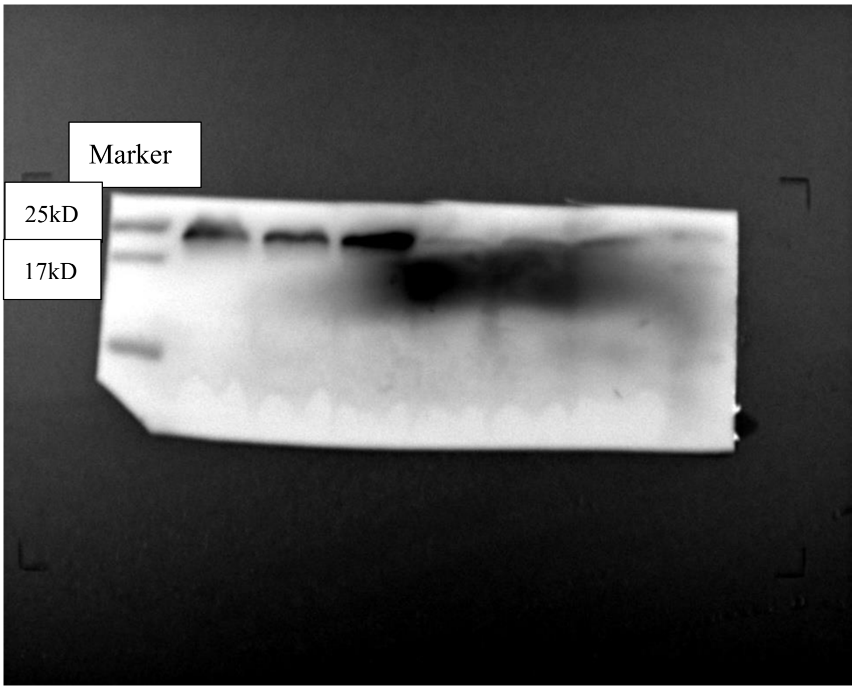

Repeat test 2

Left to right: HGC-27 (shNC + inhibitor-NC, shFAAH + inhibitor-NC, shFAAH + inhibitor-1275), MKN-1 (pcDNA + mimic-NC, pcFAAH + mimic-NC, pcFAAH + mimic-1275)

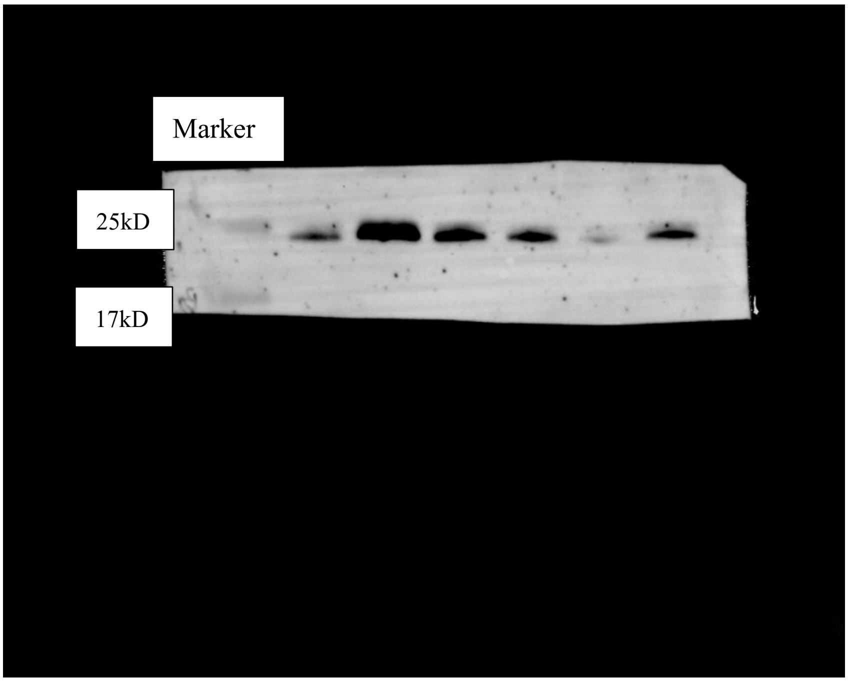

Repeat test 3

Left to right: HGC-27 (shNC + inhibitor-NC, shFAAH + inhibitor-NC, shFAAH + inhibitor-1275), MKN-1 (pcDNA + mimic-NC, pcFAAH + mimic-NC, pcFAAH + mimic-1275)

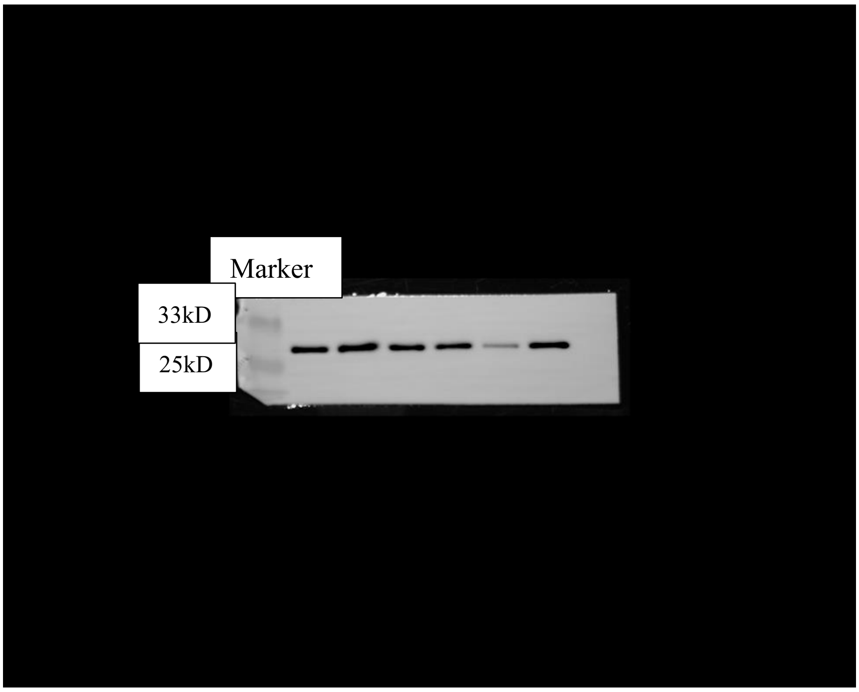

PARP-1 (113kD)

Repeat test 1

Left to right: HGC-27 (shNC + inhibitor-NC, shFAAH + inhibitor-NC, shFAAH + inhibitor-1275), MKN-1 (pcDNA + mimic-NC, pcFAAH + mimic-NC, pcFAAH + mimic-1275)

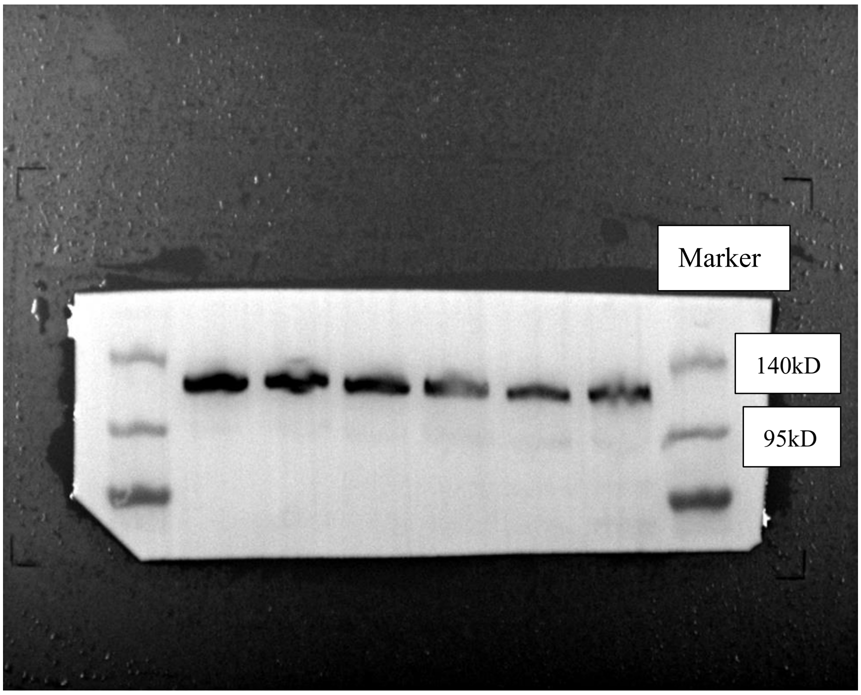

Repeat test 2

Left to right: HGC-27 (shNC + inhibitor-NC, shFAAH + inhibitor-NC, shFAAH + inhibitor-1275), MKN-1 (pcDNA + mimic-NC, pcFAAH + mimic-NC, pcFAAH + mimic-1275)

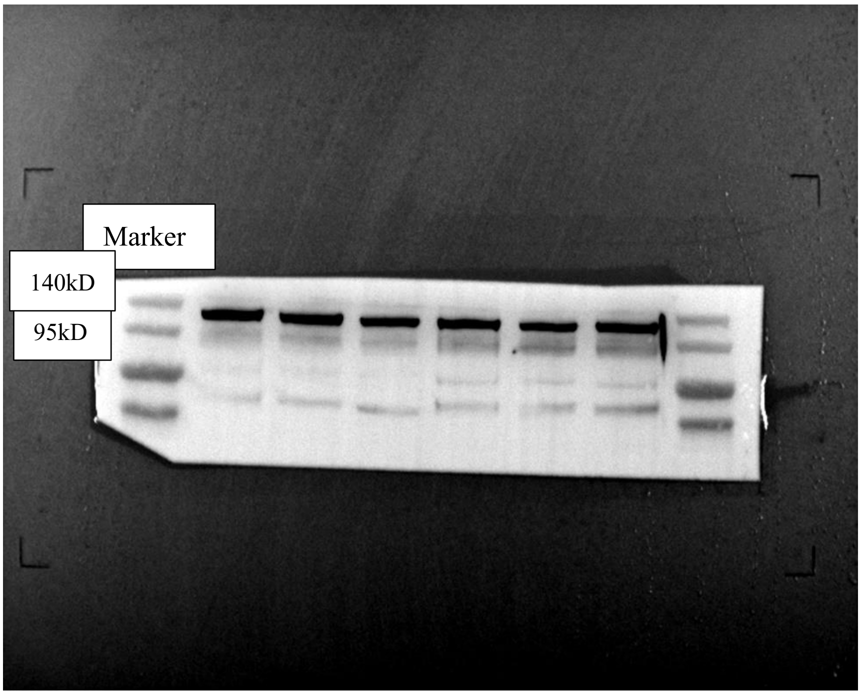

Repeat test 3

Left to right: HGC-27 (shNC + inhibitor-NC, shFAAH + inhibitor-NC, shFAAH + inhibitor-1275), MKN-1 (pcDNA + mimic-NC, pcFAAH + mimic-NC, pcFAAH + mimic-1275)

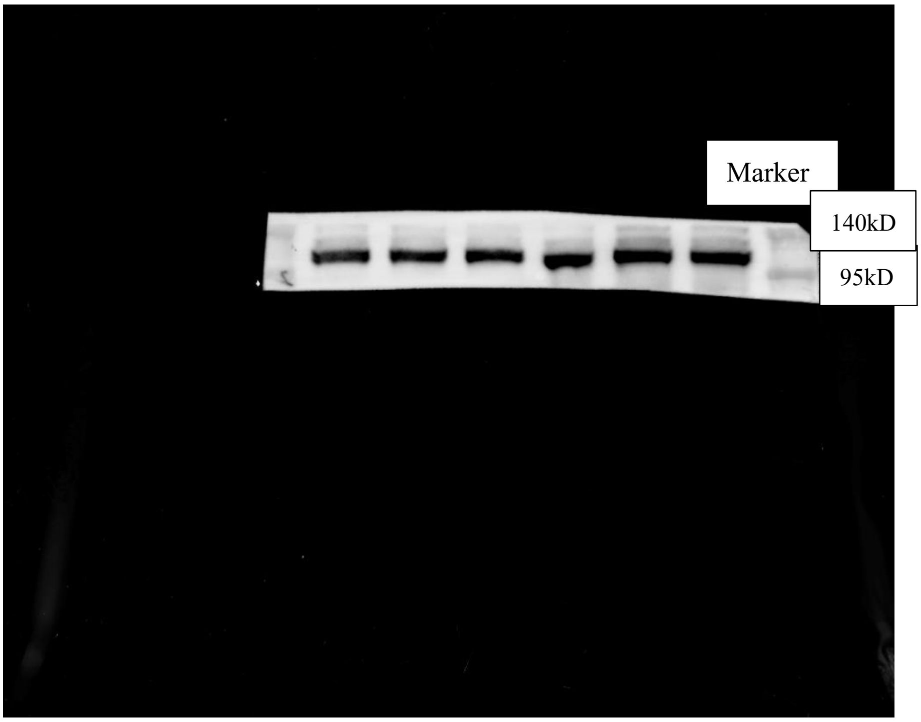

Cleaved PARP-1 (25kD)

Repeat test 1

Left to right: HGC-27 (shNC + inhibitor-NC, shFAAH + inhibitor-NC, shFAAH + inhibitor-1275), MKN-1 (pcDNA + mimic-NC, pcFAAH + mimic-NC, pcFAAH + mimic-1275)

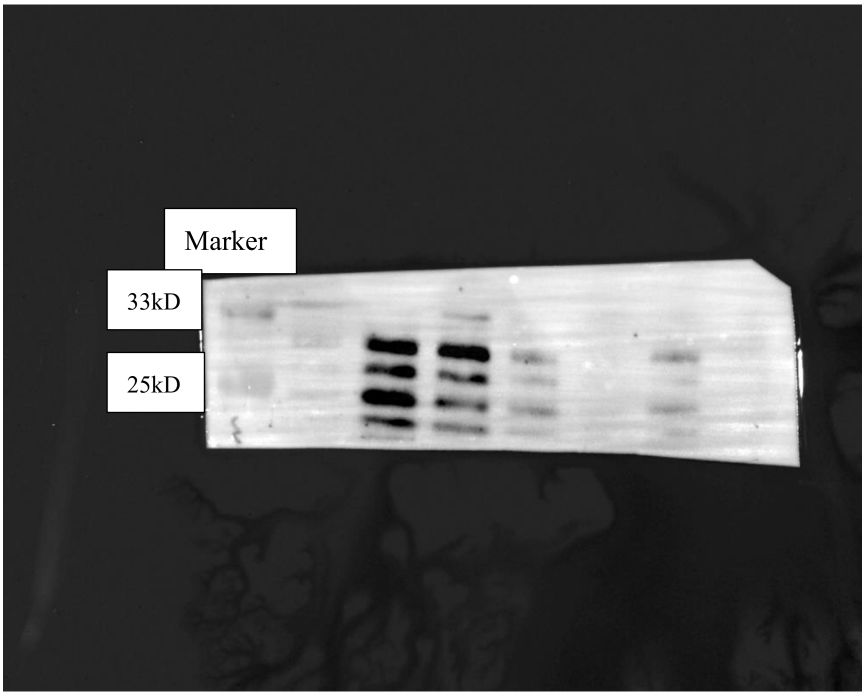

Repeat test 2

Left to right: HGC-27 (shNC + inhibitor-NC, shFAAH + inhibitor-NC, shFAAH + inhibitor-1275), MKN-1 (pcDNA + mimic-NC, pcFAAH + mimic-NC, pcFAAH + mimic-1275)

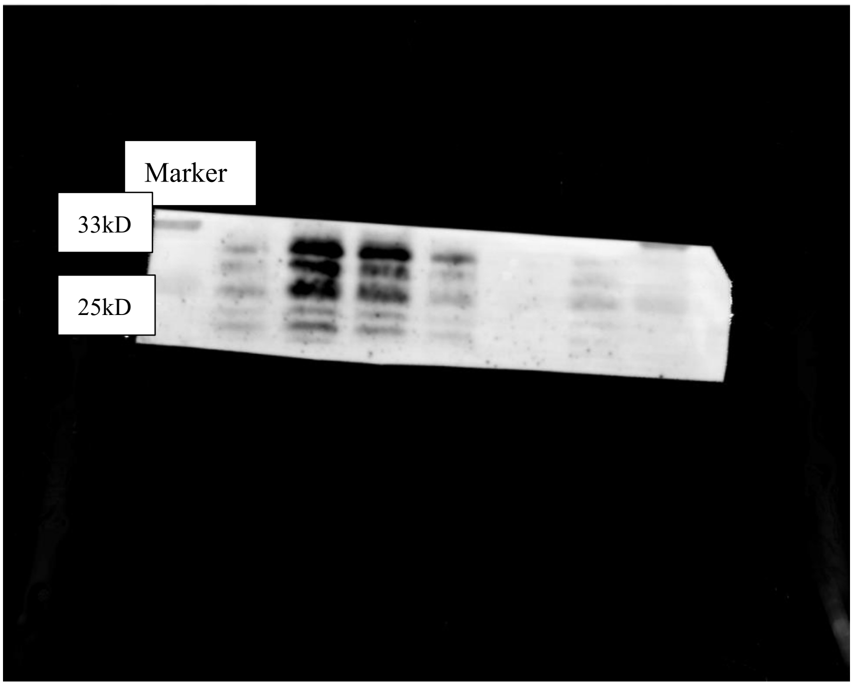

Repeat test 3

Left to right: HGC-27 (shNC + inhibitor-NC, shFAAH + inhibitor-NC, shFAAH + inhibitor-1275), MKN-1 (pcDNA + mimic-NC, pcFAAH + mimic-NC, pcFAAH + mimic-1275)

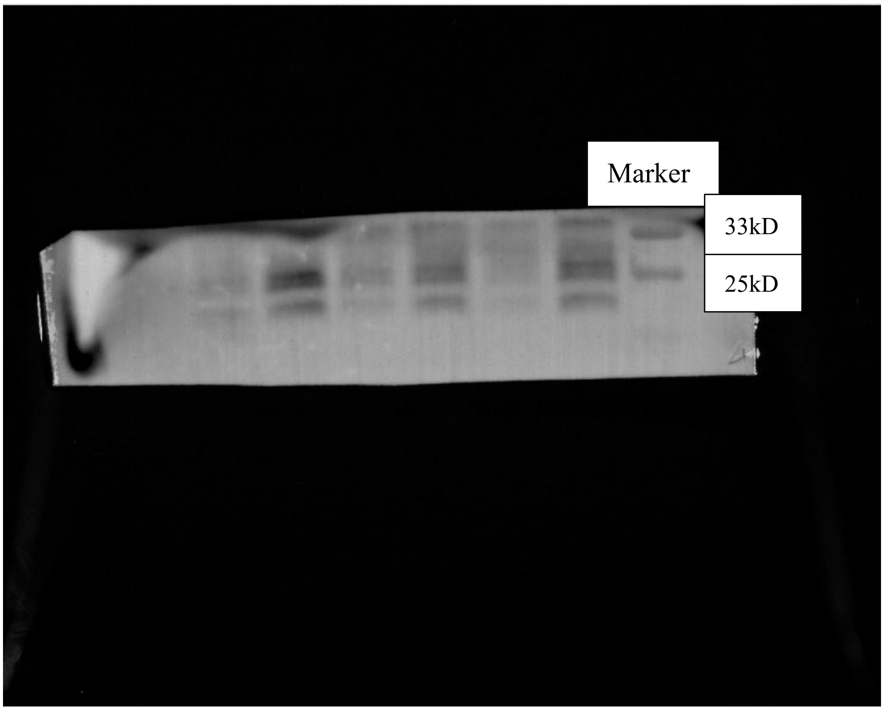

Caspase 3 (35kD)

Repeat test 1

Left to right: HGC-27 (shNC + inhibitor-NC, shFAAH + inhibitor-NC, shFAAH + inhibitor-1275), MKN-1 (pcDNA + mimic-NC, pcFAAH + mimic-NC, pcFAAH + mimic-1275)

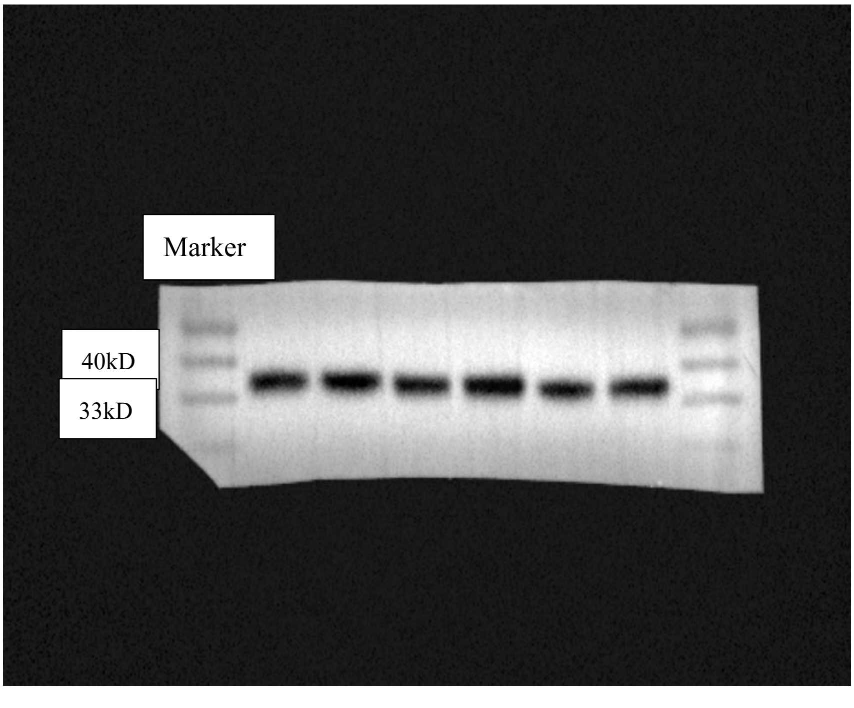

Repeat test 2

Left to right: HGC-27 (shNC + inhibitor-NC, shFAAH + inhibitor-NC, shFAAH + inhibitor-1275), MKN-1 (pcDNA + mimic-NC, pcFAAH + mimic-NC, pcFAAH + mimic-1275)

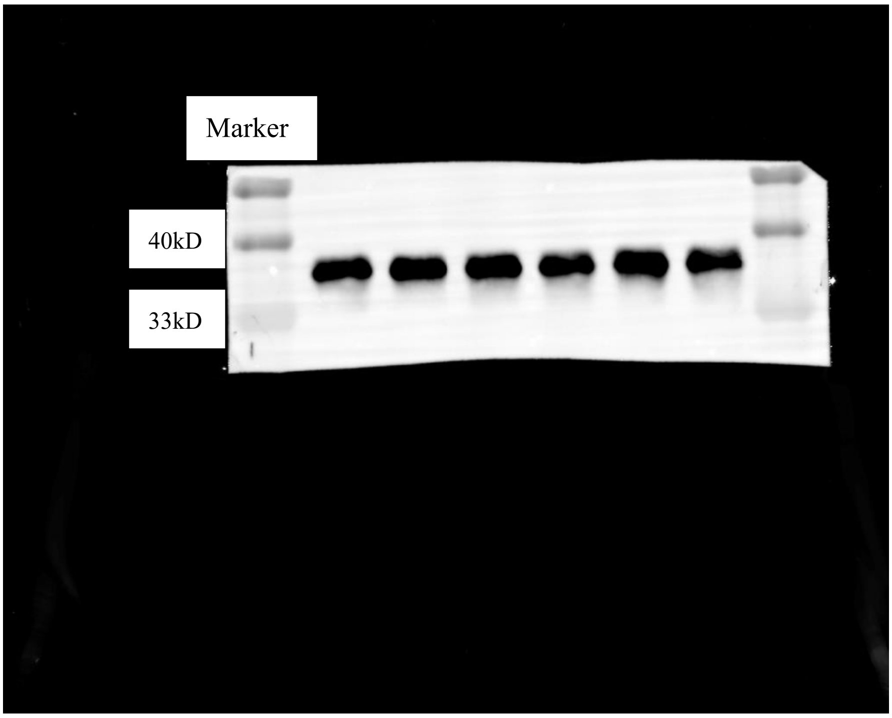

Repeat test 3

Left to right: HGC-27 (shNC + inhibitor-NC, shFAAH + inhibitor-NC, shFAAH + inhibitor-1275), MKN-1 (pcDNA + mimic-NC, pcFAAH + mimic-NC, pcFAAH + mimic-1275)

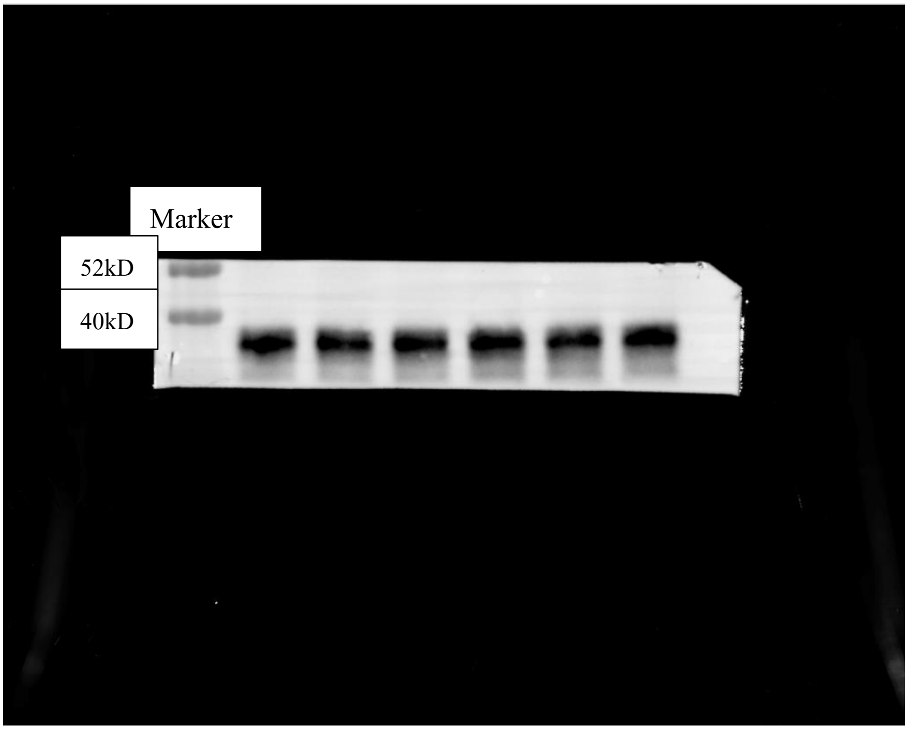

Cleaved caspase-3 (17/19kD)

Repeat test 1

Left to right: HGC-27 (shNC + inhibitor-NC, shFAAH + inhibitor-NC, shFAAH + inhibitor-1275), MKN-1 (pcDNA + mimic-NC, pcFAAH + mimic-NC, pcFAAH + mimic-1275)

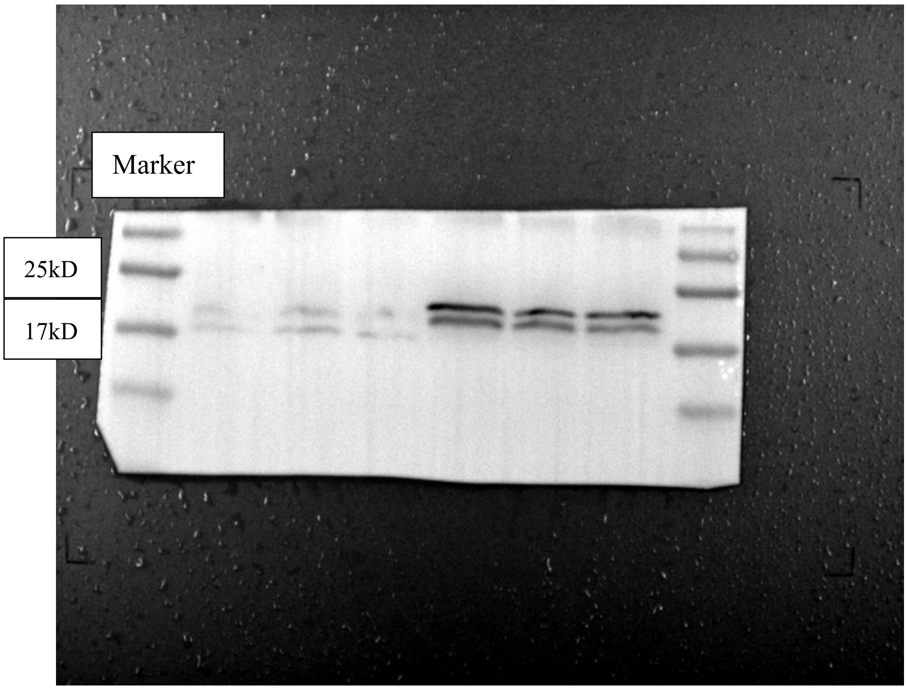

Repeat test 2

Left to right: HGC-27 (shNC + inhibitor-NC, shFAAH + inhibitor-NC, shFAAH + inhibitor-1275), MKN-1 (pcDNA + mimic-NC, pcFAAH + mimic-NC, pcFAAH + mimic-1275)

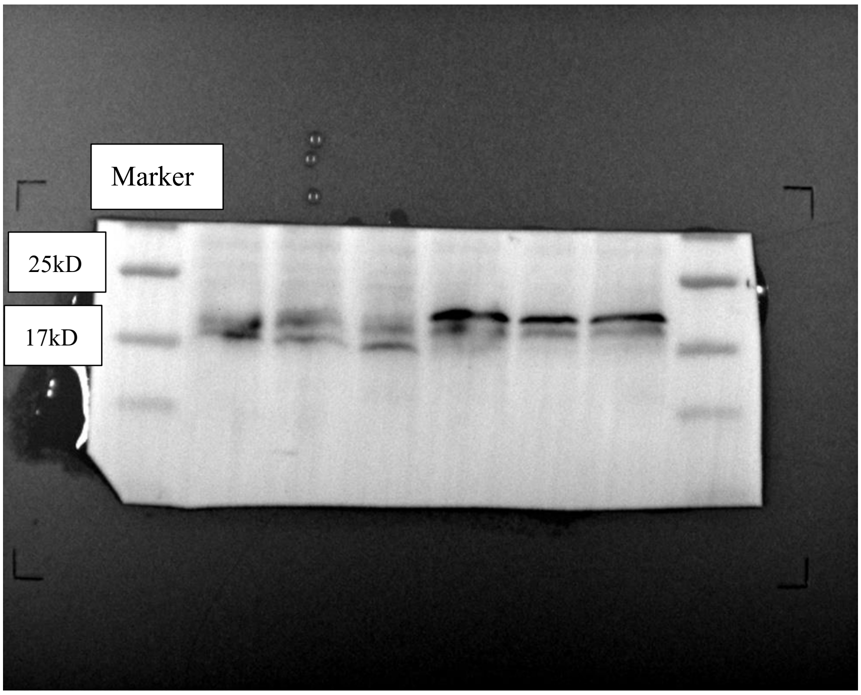

Repeat test 3

Left to right: HGC-27 (shNC + inhibitor-NC, shFAAH + inhibitor-NC, shFAAH + inhibitor-1275), MKN-1 (pcDNA + mimic-NC, pcFAAH + mimic-NC, pcFAAH + mimic-1275)

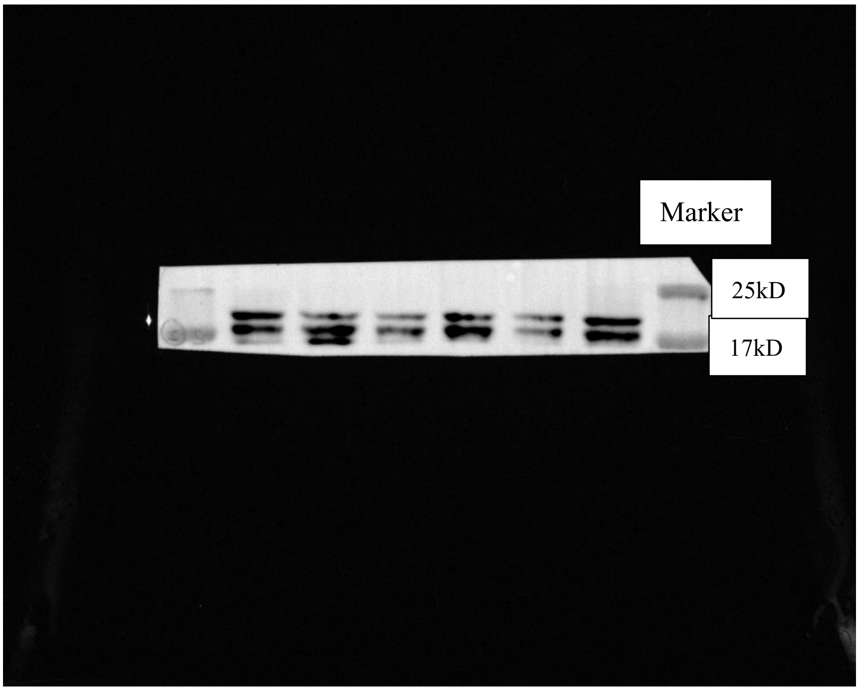

Bcl2 (26kD)

Repeat test 1

Left to right: HGC-27 (shNC + inhibitor-NC, shFAAH + inhibitor-NC, shFAAH + inhibitor-1275), MKN-1 (pcDNA + mimic-NC, pcFAAH + mimic-NC, pcFAAH + mimic-1275)

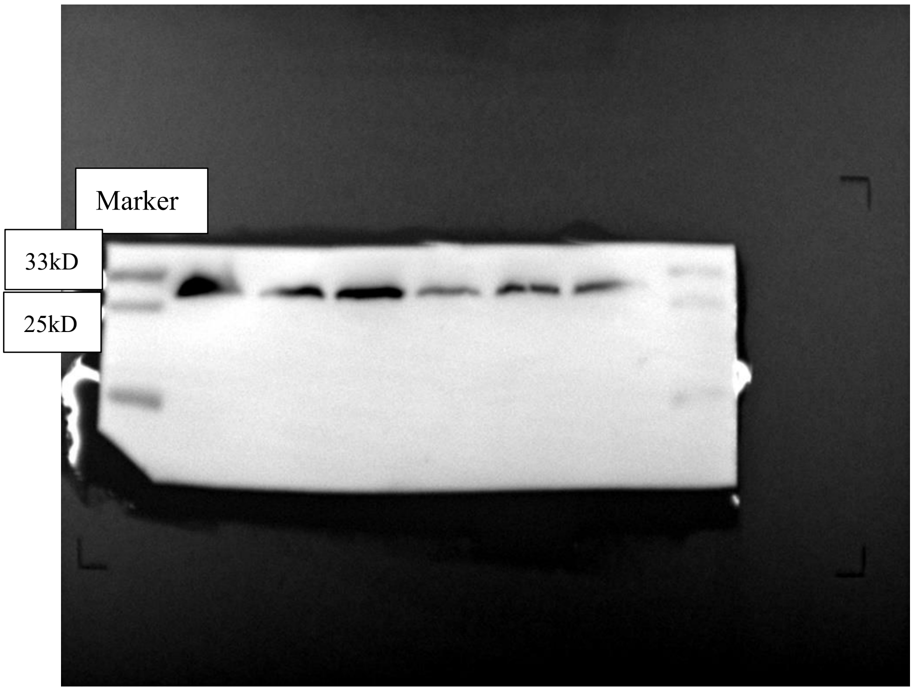

Repeat test 2

Left to right: HGC-27 (shNC + inhibitor-NC, shFAAH + inhibitor-NC, shFAAH + inhibitor-1275), MKN-1 (pcDNA + mimic-NC, pcFAAH + mimic-NC, pcFAAH + mimic-1275)

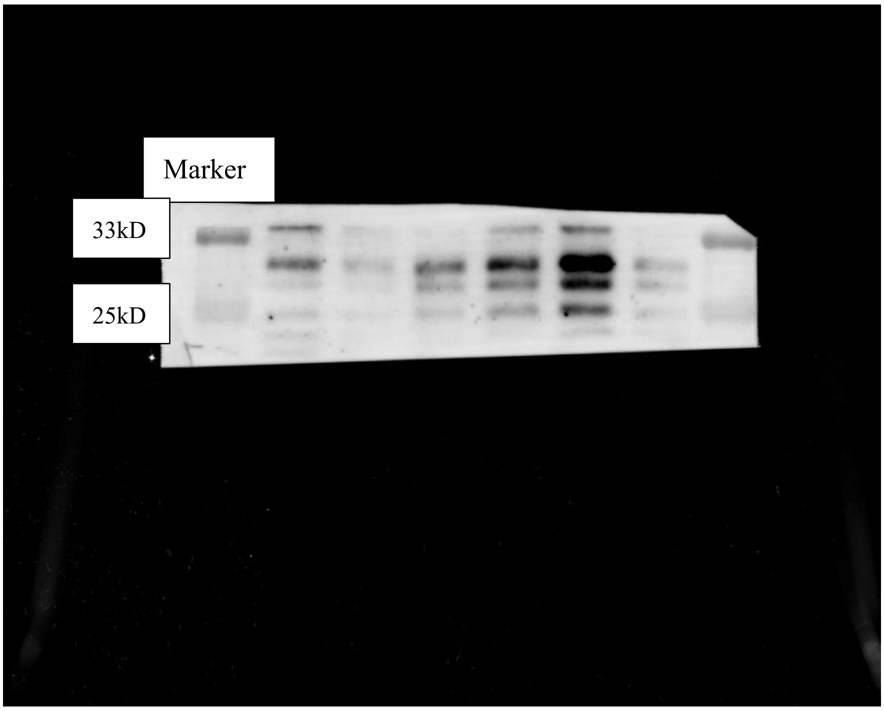

Repeat test 3

Left to right: HGC-27 (shNC + inhibitor-NC, shFAAH + inhibitor-NC, shFAAH + inhibitor-1275), MKN-1 (pcDNA + mimic-NC, pcFAAH + mimic-NC, pcFAAH + mimic-1275)

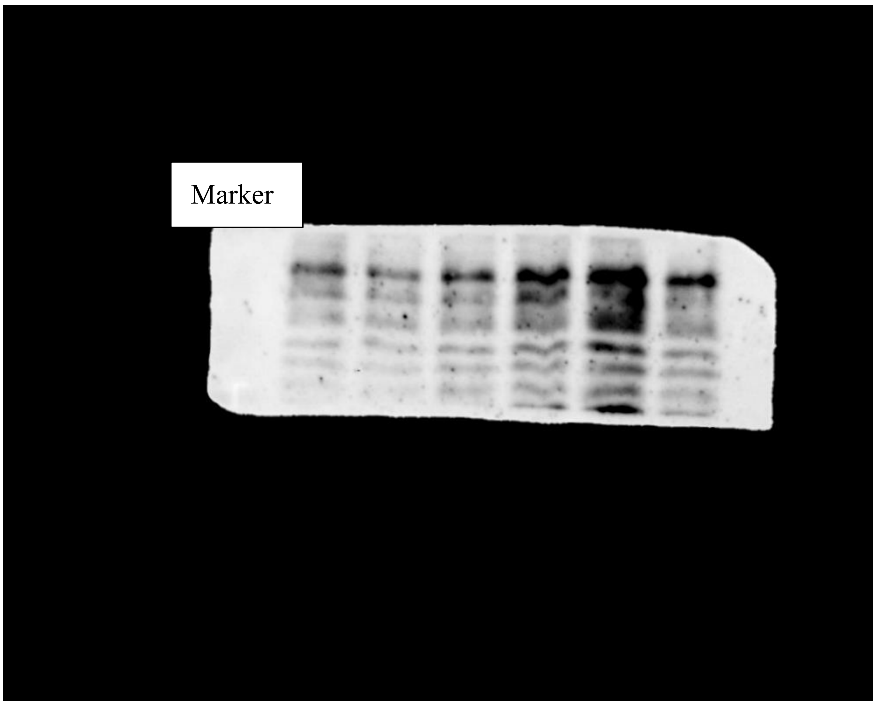

Bax (21kD)

Repeat test 1

Left to right: HGC-27 (shNC + inhibitor-NC, shFAAH + inhibitor-NC, shFAAH + inhibitor-1275), MKN-1 (pcDNA + mimic-NC, pcFAAH + mimic-NC, pcFAAH + mimic-1275)

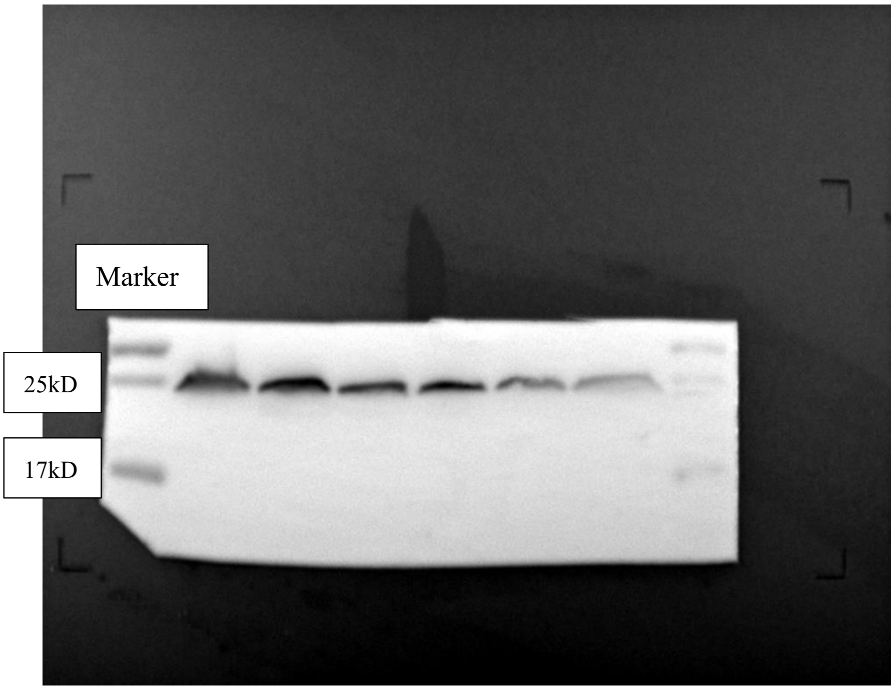

Repeat test 2

Left to right: HGC-27 (shNC + inhibitor-NC, shFAAH + inhibitor-NC, shFAAH + inhibitor-1275), MKN-1 (pcDNA + mimic-NC, pcFAAH + mimic-NC, pcFAAH + mimic-1275)

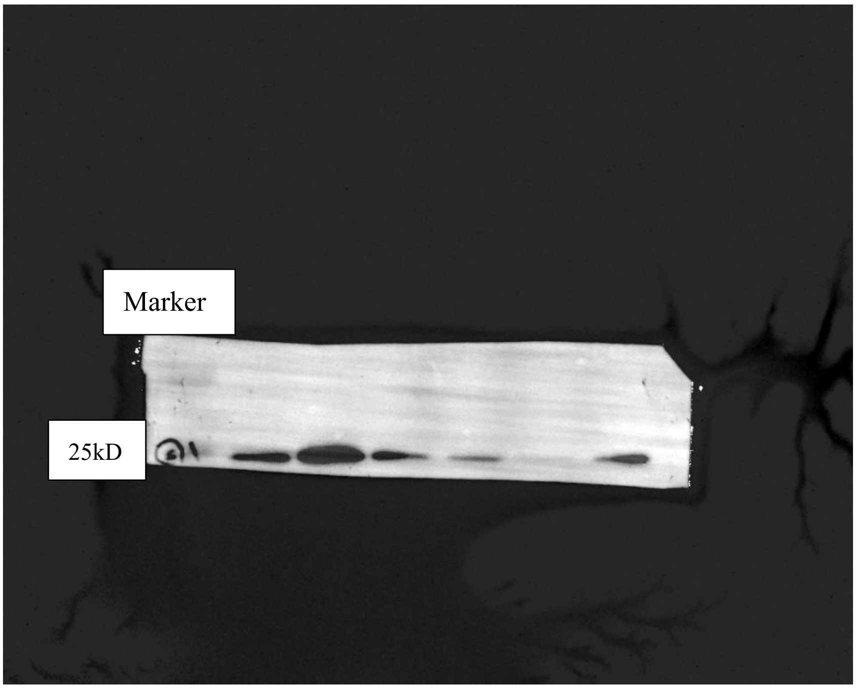

Repeat test 3

Left to right: HGC-27 (shNC + inhibitor-NC, shFAAH + inhibitor-NC, shFAAH + inhibitor-1275), MKN-1 (pcDNA + mimic-NC, pcFAAH + mimic-NC, pcFAAH + mimic-1275)

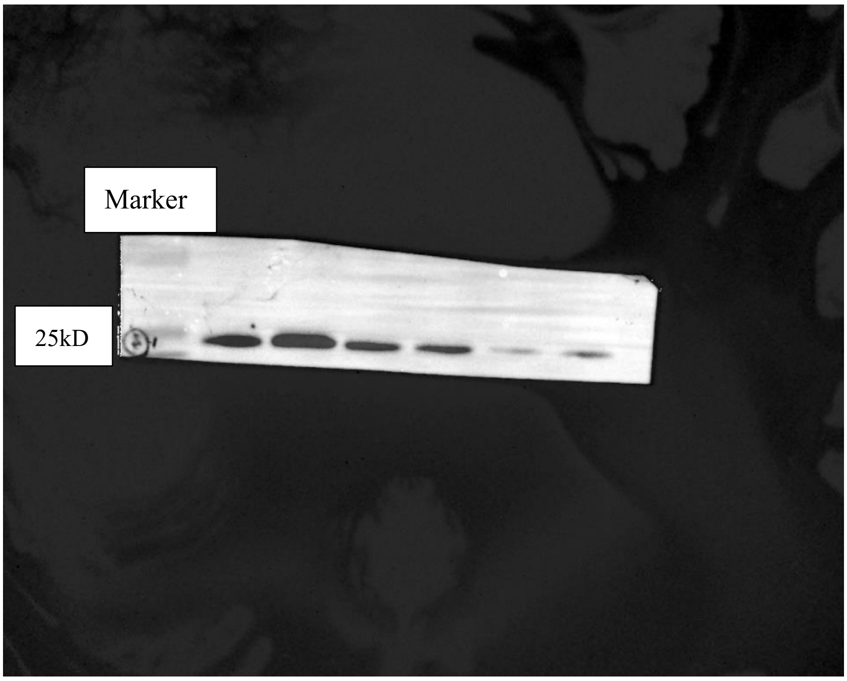

β-actin (42kD)

Repeat test 1

Left to right: HGC-27 (shNC + inhibitor-NC, shFAAH + inhibitor-NC, shFAAH + inhibitor-1275), MKN-1 (pcDNA + mimic-NC, pcFAAH + mimic-NC, pcFAAH + mimic-1275)

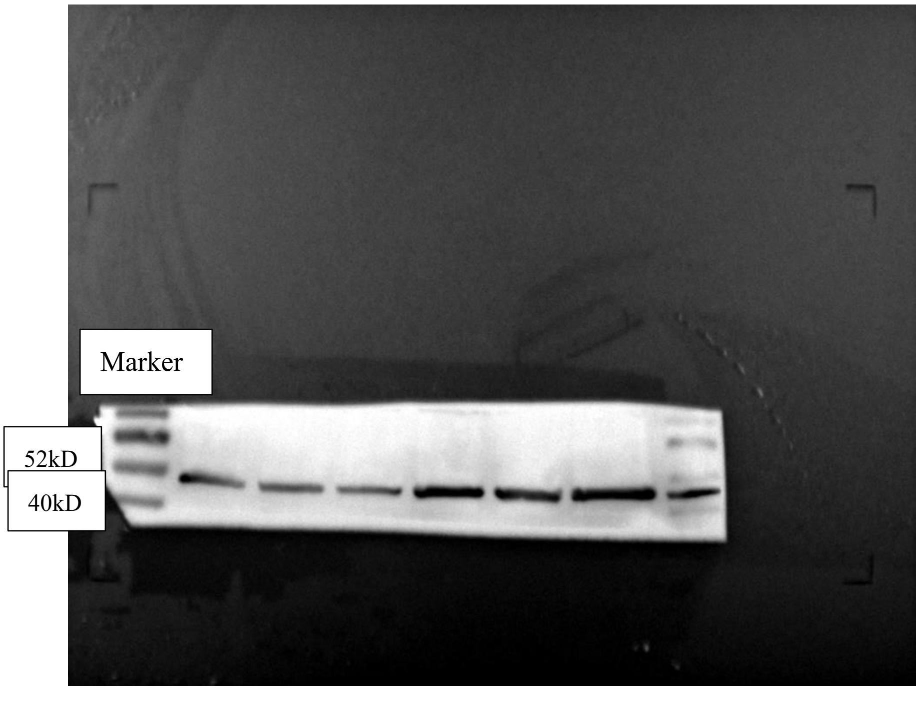

Repeat test 2

Left to right: HGC-27 (shNC + inhibitor-NC, shFAAH + inhibitor-NC, shFAAH + inhibitor-1275), MKN-1 (pcDNA + mimic-NC, pcFAAH + mimic-NC, pcFAAH + mimic-1275)

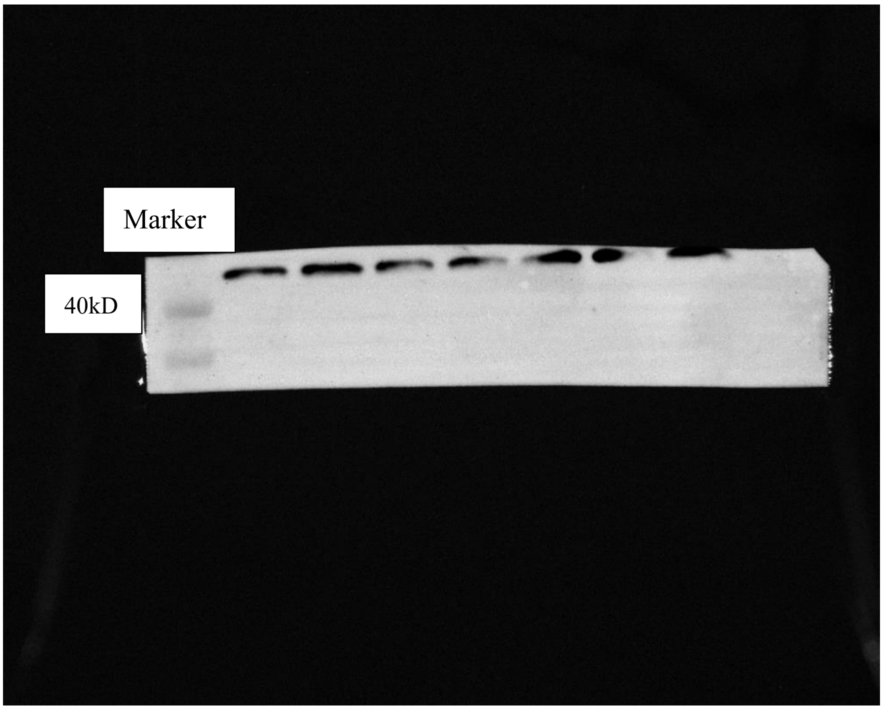

Repeat test 3

Left to right: HGC-27 (shNC + inhibitor-NC, shFAAH + inhibitor-NC, shFAAH + inhibitor-1275), MKN-1 (pcDNA + mimic-NC, pcFAAH + mimic-NC, pcFAAH + mimic-1275)

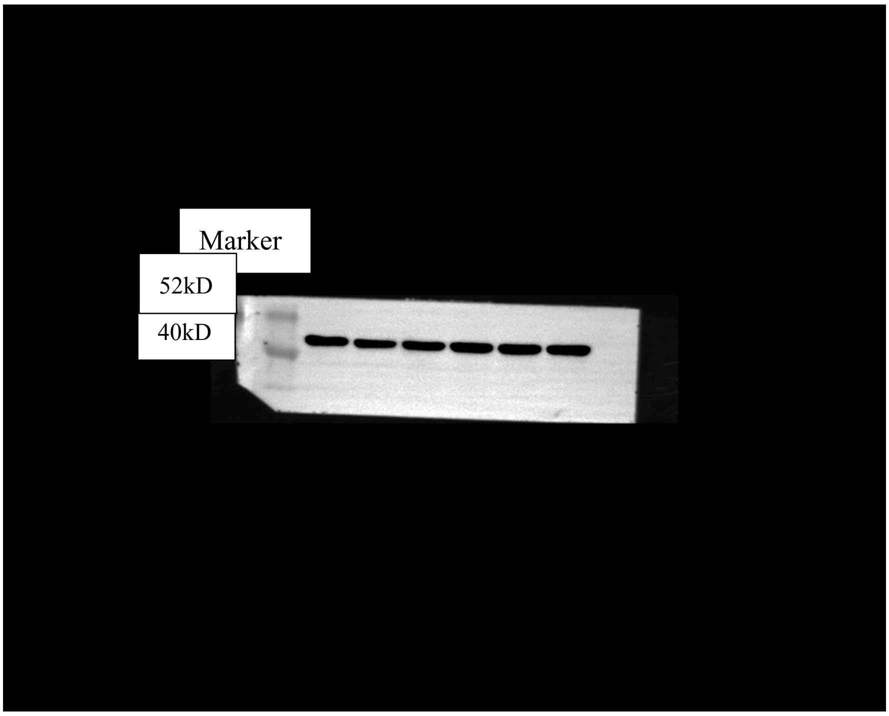

Figure 6f

HIF-1α (120kD)

Left to right: HGC-27 (shNC, shFAAH1, shFAAH2); MKN-1 (shNC, shFAAH1, shFAAH2)

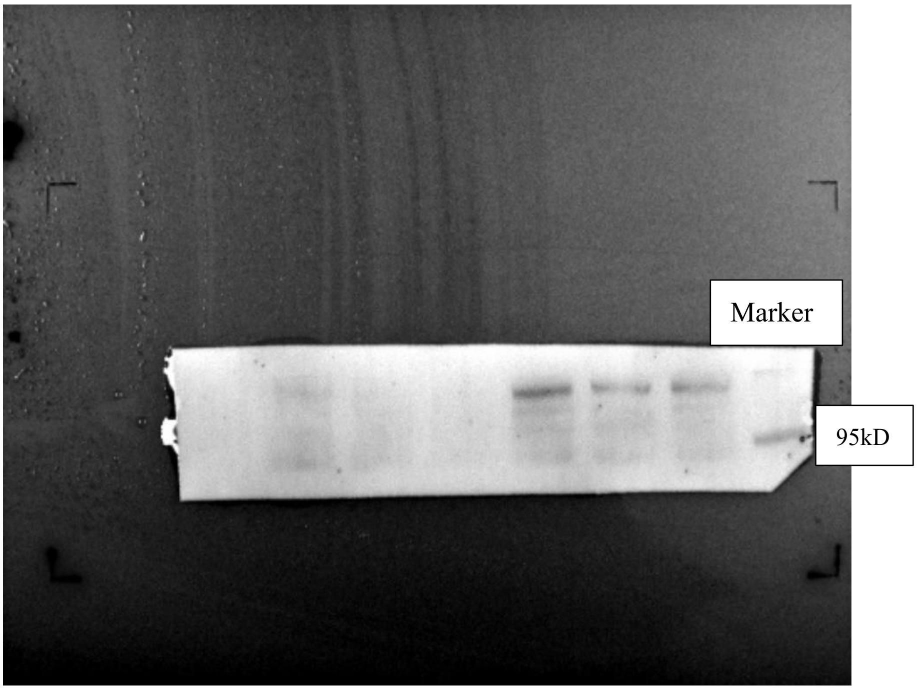

COX-1 (69kD)

Left to right: HGC-27 (shNC, shFAAH1, shFAAH2); MKN-1 (shNC, shFAAH1, shFAAH2)

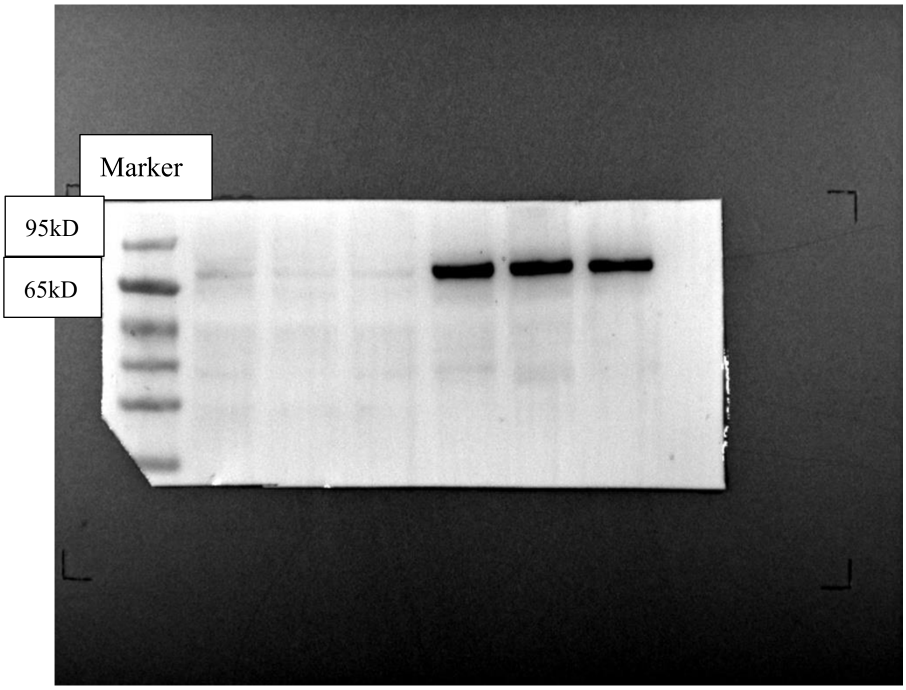

COX-2 (69kD)

Left to right: HGC-27 (shNC, shFAAH1, shFAAH2); MKN-1 (shNC, shFAAH1, shFAAH2)

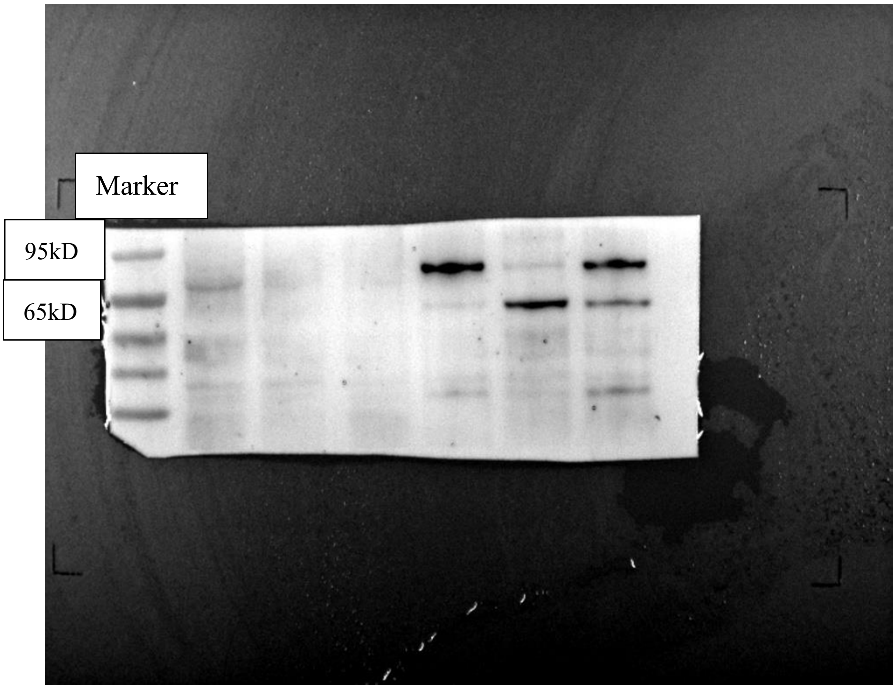

CB1 (60kD)

Left to right: HGC-27 (shNC, shFAAH1, shFAAH2); MKN-1 (shNC, shFAAH1, shFAAH2)

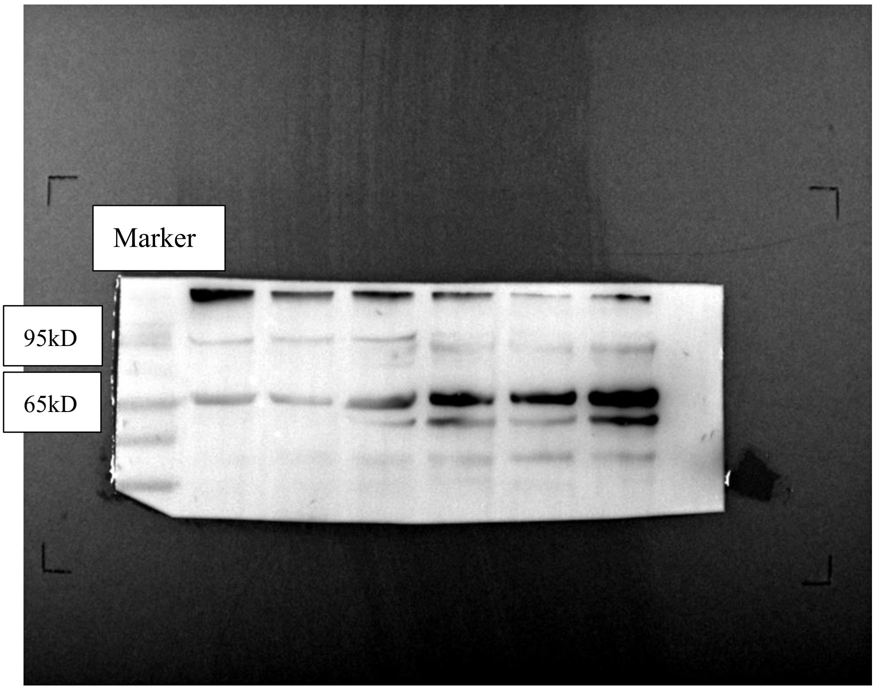

CB2 (40kD)

Left to right: HGC-27 (shNC, shFAAH1, shFAAH2); MKN-1 (shNC, shFAAH1, shFAAH2)

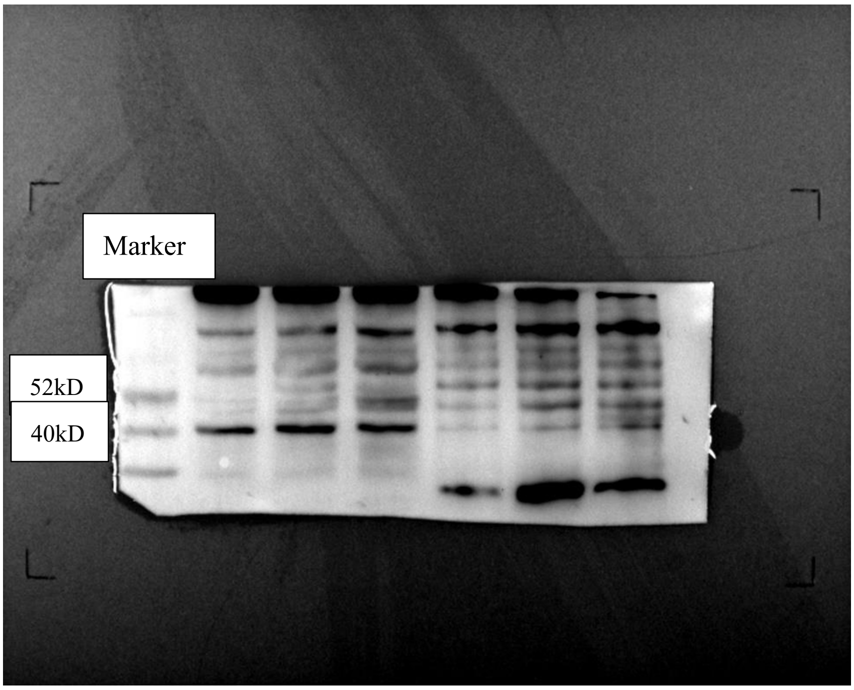

NAT-1 (34kD)

Left to right: HGC-27 (shNC, shFAAH1, shFAAH2); MKN-1 (shNC, shFAAH1, shFAAH2)

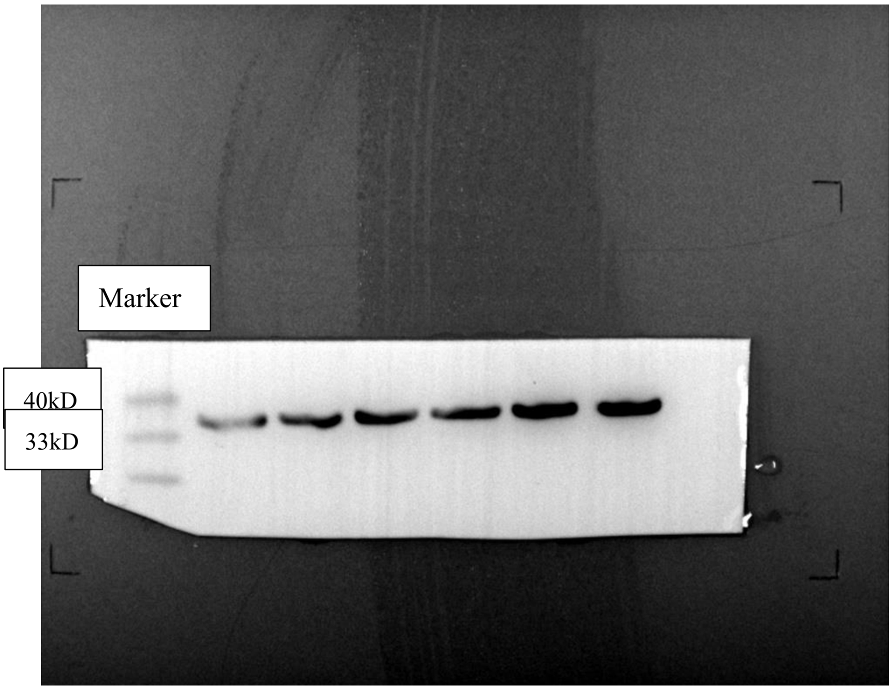

NAPE-PLD (46kD)

Left to right: HGC-27 (shNC, shFAAH1, shFAAH2); MKN-1 (shNC, shFAAH1, shFAAH2)

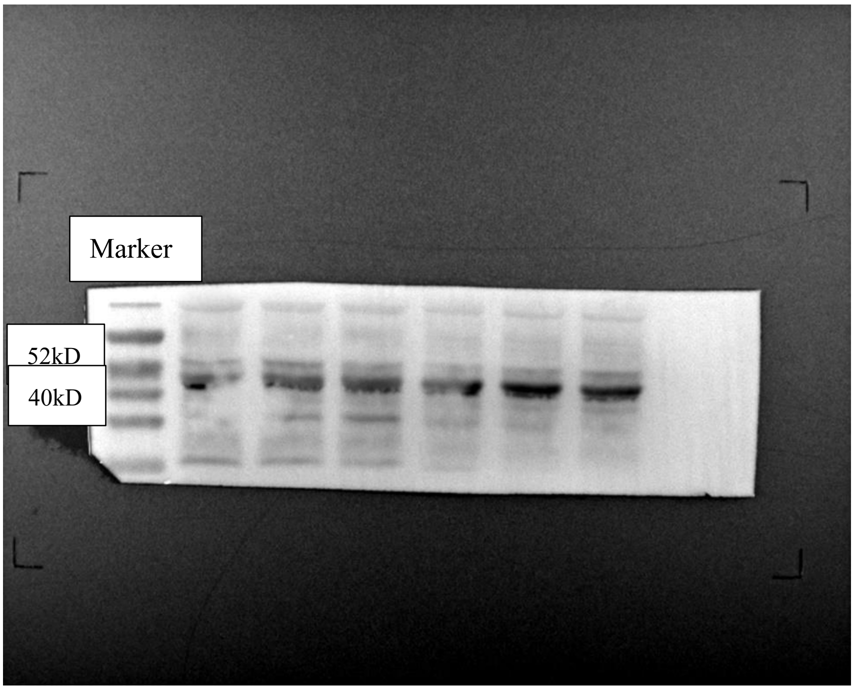

β-actin (42kD)

Left to right: HGC-27 (shNC, shFAAH1, shFAAH2); MKN-1 (shNC, shFAAH1, shFAAH2)

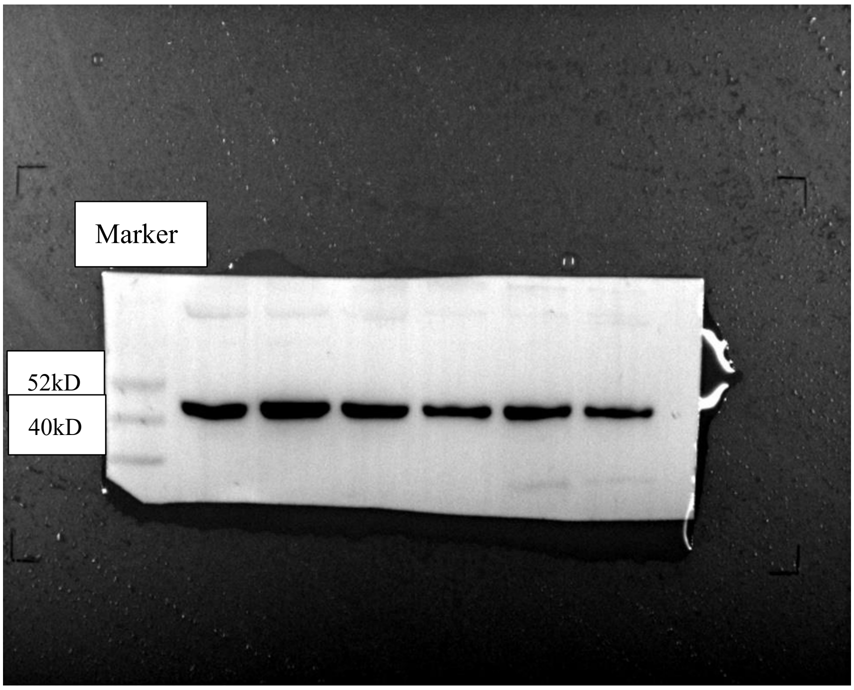

Figure 6h
HIF-1α (120kD)
Repeat test 1
Left to right: HGC-27 (shNC + inhibitor-NC, shFAAH + inhibitor-NC, shFAAH + inhibitor-1275), MKN-1 (pcDNA + mimic-NC, pcFAAH + mimic-NC, pcFAAH + mimic-1275)

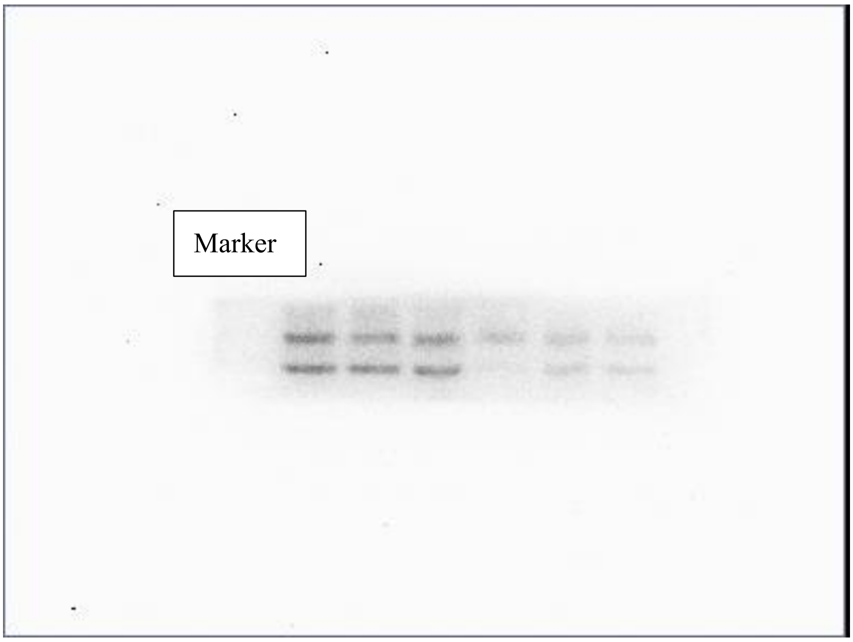

Repeat test 2
Left to right: HGC-27 (shNC + inhibitor-NC, shFAAH + inhibitor-NC, shFAAH + inhibitor-1275), MKN-1 (pcDNA + mimic-NC, pcFAAH + mimic-NC, pcFAAH + mimic-1275)

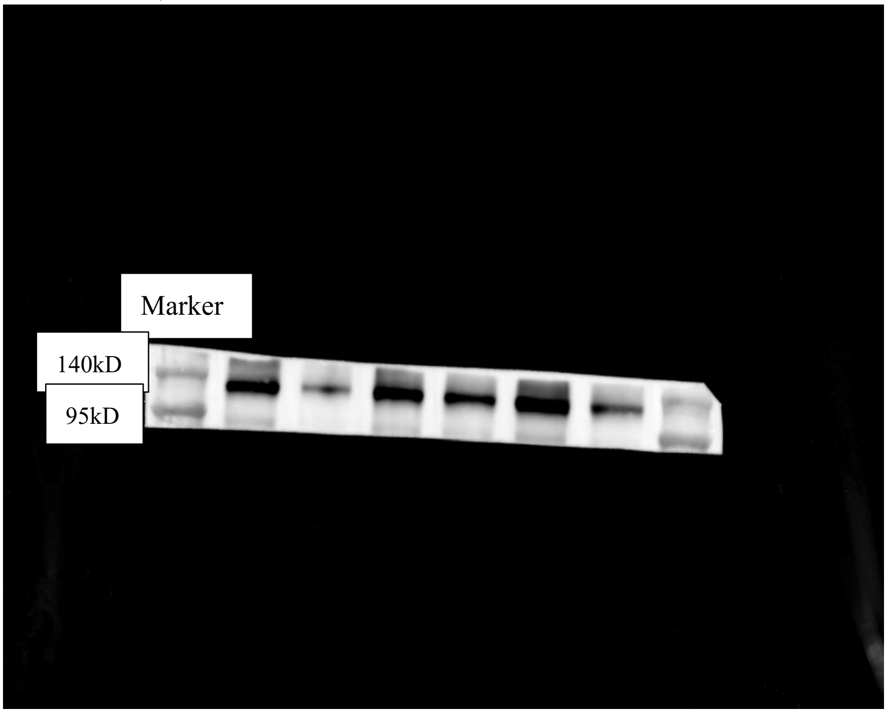

Repeat test 3

Left to right: HGC-27 (shNC + inhibitor-NC, shFAAH + inhibitor-NC, shFAAH + inhibitor-1275), MKN-1 (pcDNA + mimic-NC, pcFAAH + mimic-NC, pcFAAH + mimic-1275)

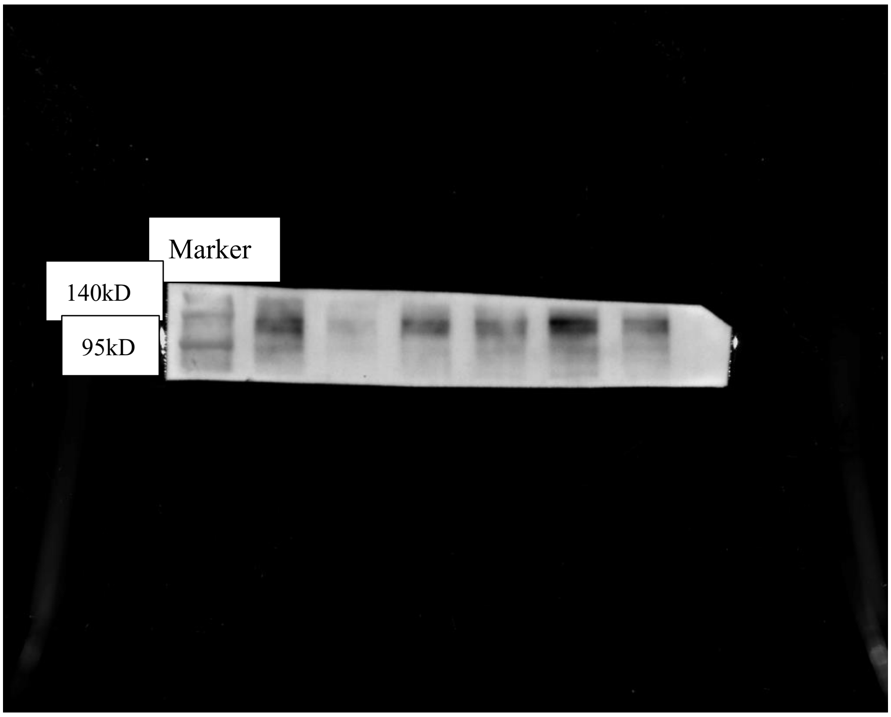

COX-2 (69kD)

Repeat test 1

Left to right: HGC-27 (shNC + inhibitor-NC, shFAAH + inhibitor-NC, shFAAH + inhibitor-1275), MKN-1 (pcDNA + mimic-NC, pcFAAH + mimic-NC, pcFAAH + mimic-1275)

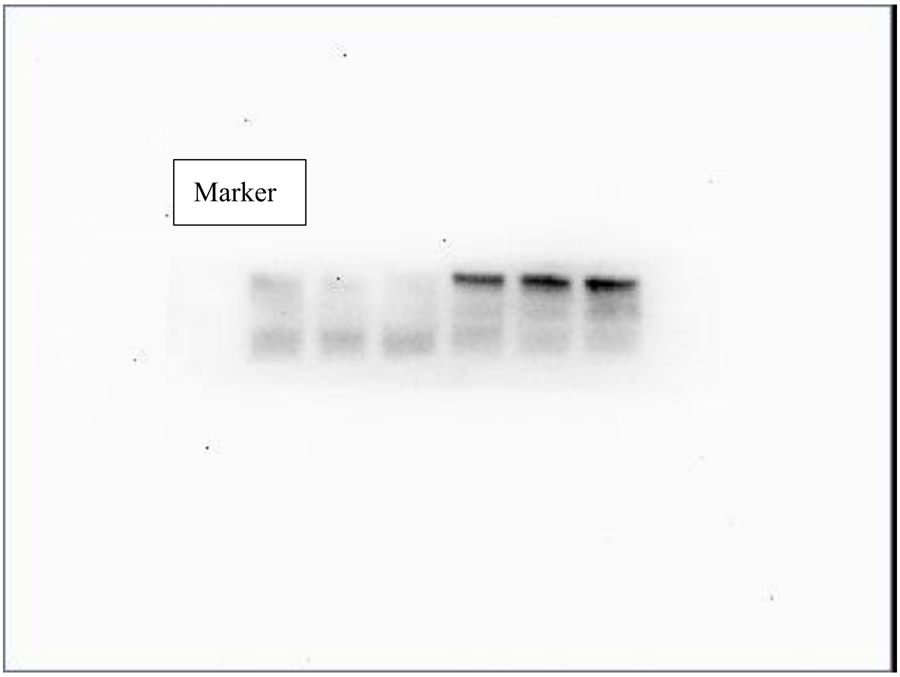

Repeat test 2

Left to right: HGC-27 (shNC + inhibitor-NC, shFAAH + inhibitor-NC, shFAAH + inhibitor-1275), MKN-1 (pcDNA + mimic-NC, pcFAAH + mimic-NC, pcFAAH + mimic-1275)

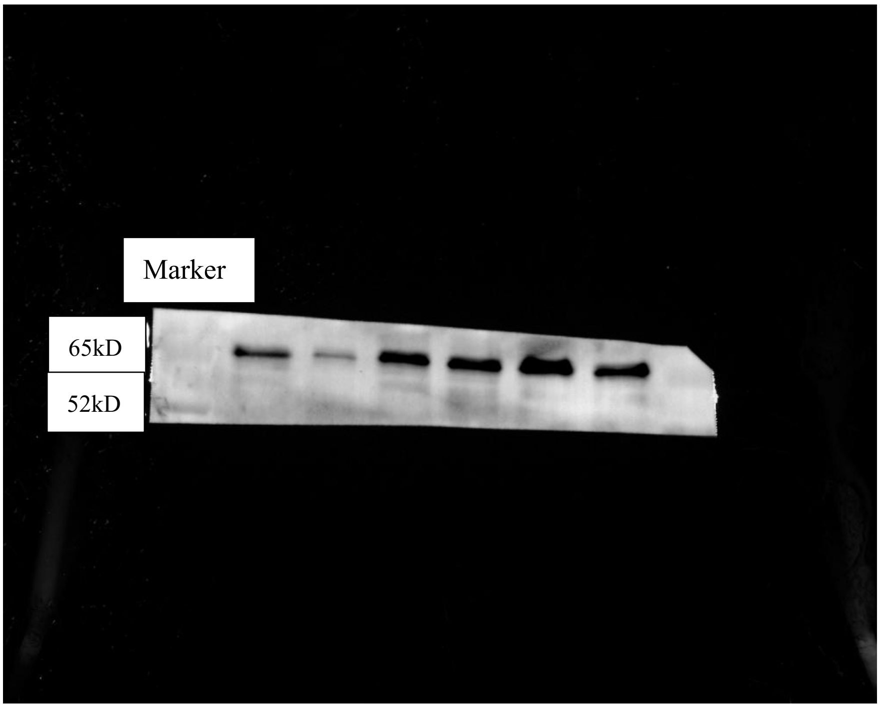

Repeat test 3

Left to right: HGC-27 (shNC + inhibitor-NC, shFAAH + inhibitor-NC, shFAAH + inhibitor-1275), MKN-1 (pcDNA + mimic-NC, pcFAAH + mimic-NC, pcFAAH + mimic-1275)

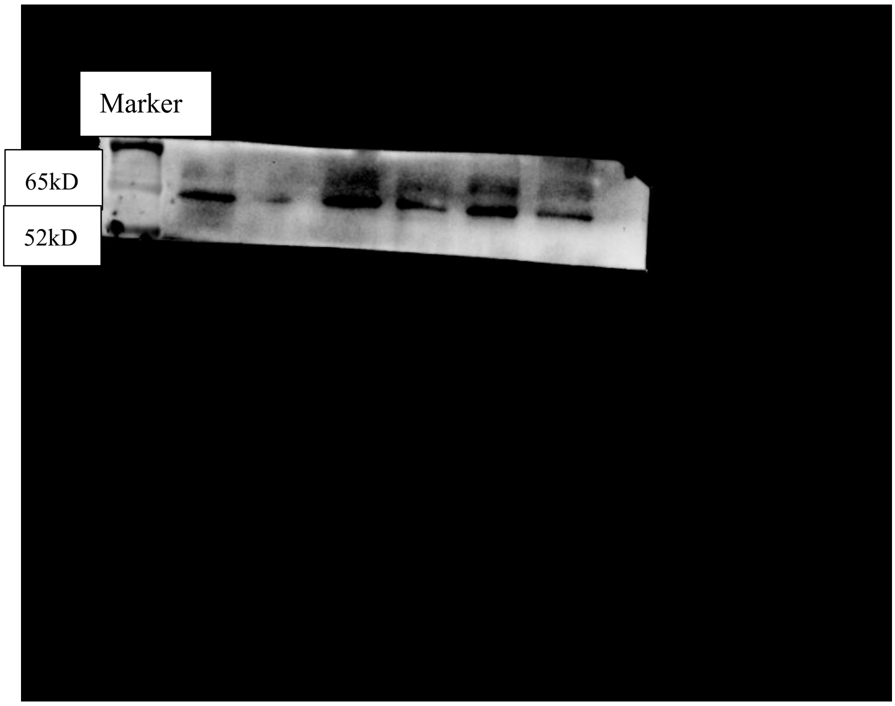

CB1 (60kD)

Repeat test 1

Left to right: HGC-27 (shNC + inhibitor-NC, shFAAH + inhibitor-NC, shFAAH + inhibitor-1275), MKN-1 (pcDNA + mimic-NC, pcFAAH + mimic-NC, pcFAAH + mimic-1275)

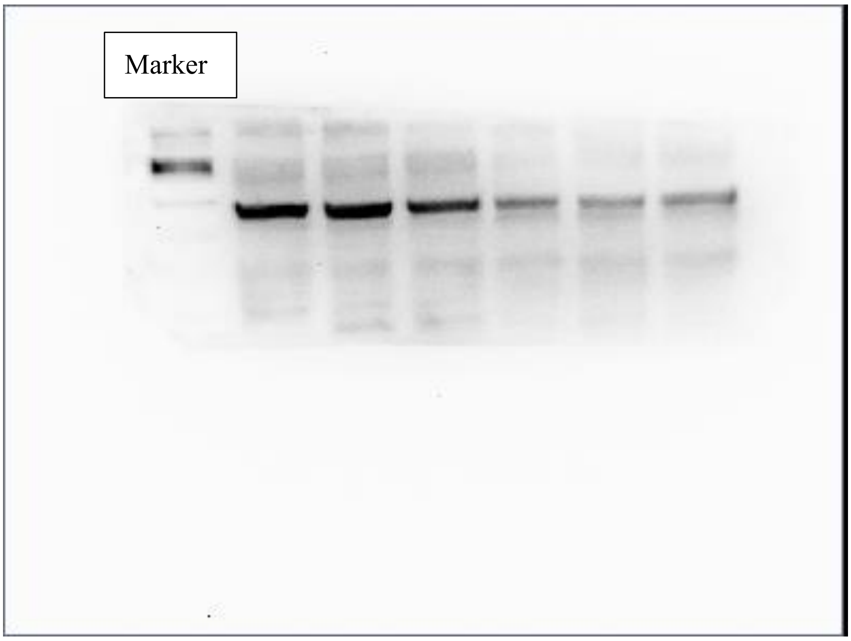

Repeat test 2

Left to right: HGC-27 (shNC + inhibitor-NC, shFAAH + inhibitor-NC, shFAAH + inhibitor-1275), MKN-1 (pcDNA + mimic-NC, pcFAAH + mimic-NC, pcFAAH + mimic-1275)

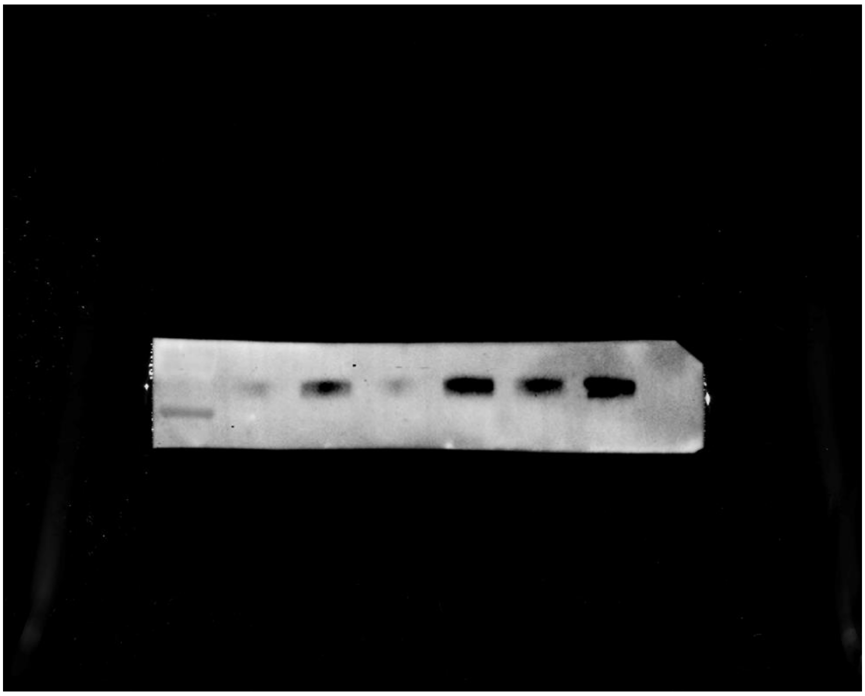

Repeat test 3

Left to right: HGC-27 (shNC + inhibitor-NC, shFAAH + inhibitor-NC, shFAAH + inhibitor-1275), MKN-1 (pcDNA + mimic-NC, pcFAAH + mimic-NC, pcFAAH + mimic-1275)

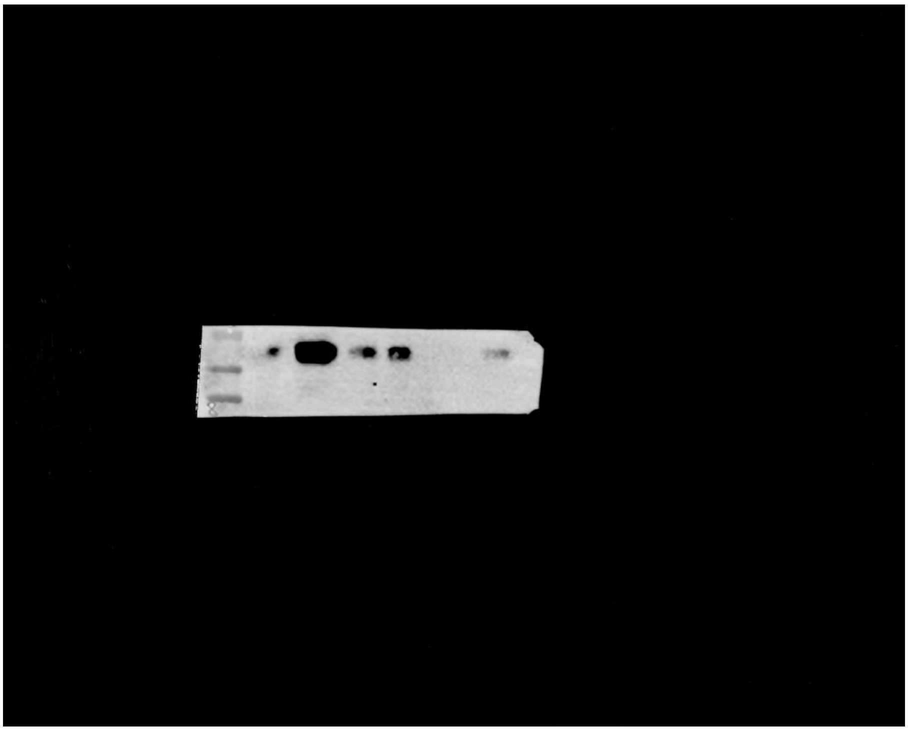

CB2 (40kD)

Repeat test 1

Left to right: HGC-27 (shNC + inhibitor-NC, shFAAH + inhibitor-NC, shFAAH + inhibitor-1275), MKN-1 (pcDNA + mimic-NC, pcFAAH + mimic-NC, pcFAAH + mimic-1275)

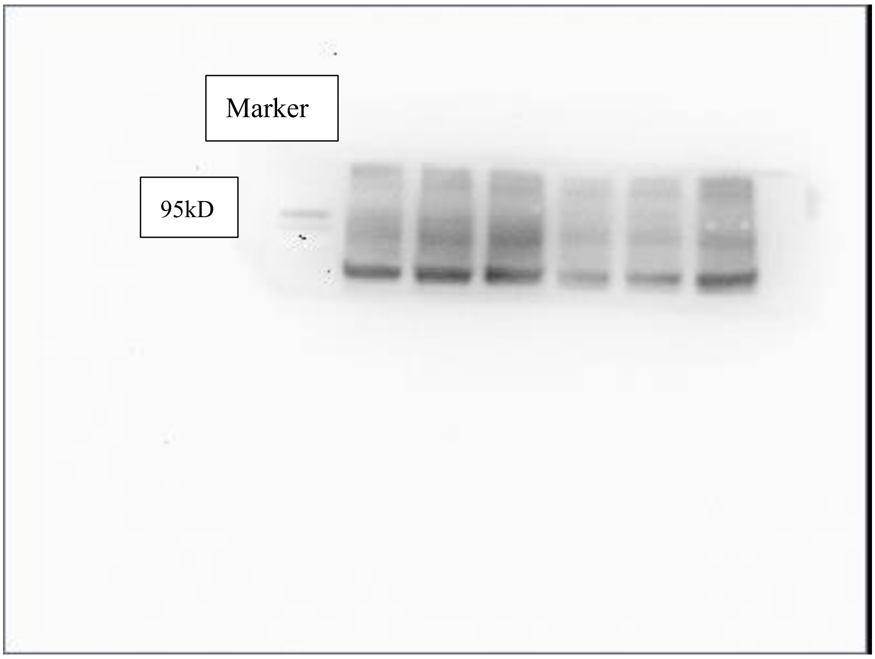

Repeat test 2

Left to right: HGC-27 (shNC + inhibitor-NC, shFAAH + inhibitor-NC, shFAAH + inhibitor-1275), MKN-1 (pcDNA + mimic-NC, pcFAAH + mimic-NC, pcFAAH + mimic-1275)

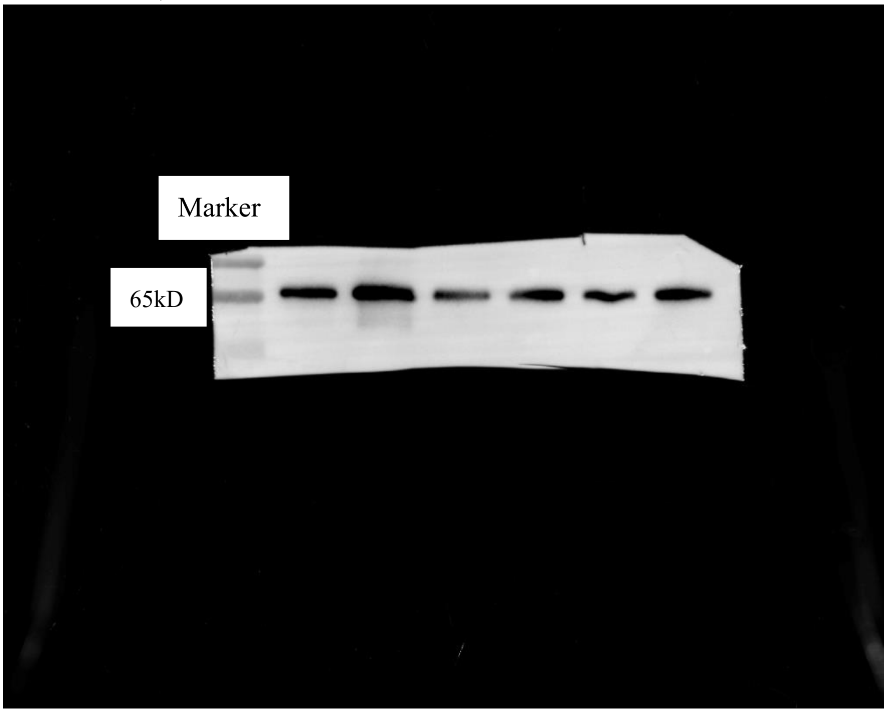

Repeat test 3

Left to right: HGC-27 (shNC + inhibitor-NC, shFAAH + inhibitor-NC, shFAAH + inhibitor-1275), MKN-1 (pcDNA + mimic-NC, pcFAAH + mimic-NC, pcFAAH + mimic-1275)

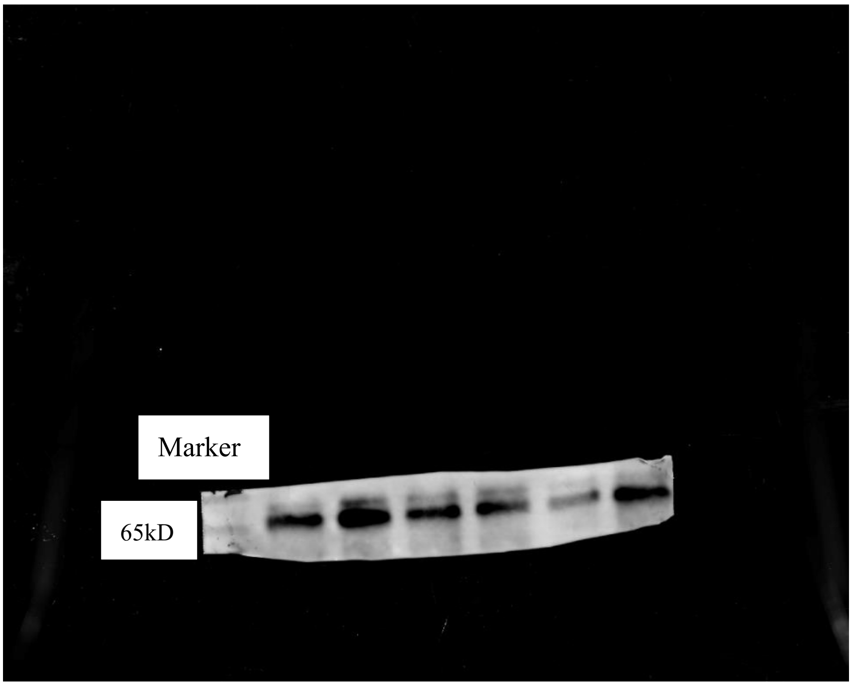

NAT-1 (34kD)

Repeat test 1

Left to right: HGC-27 (shNC + inhibitor-NC, shFAAH + inhibitor-NC, shFAAH + inhibitor-1275), MKN-1 (pcDNA + mimic-NC, pcFAAH + mimic-NC, pcFAAH + mimic-1275)

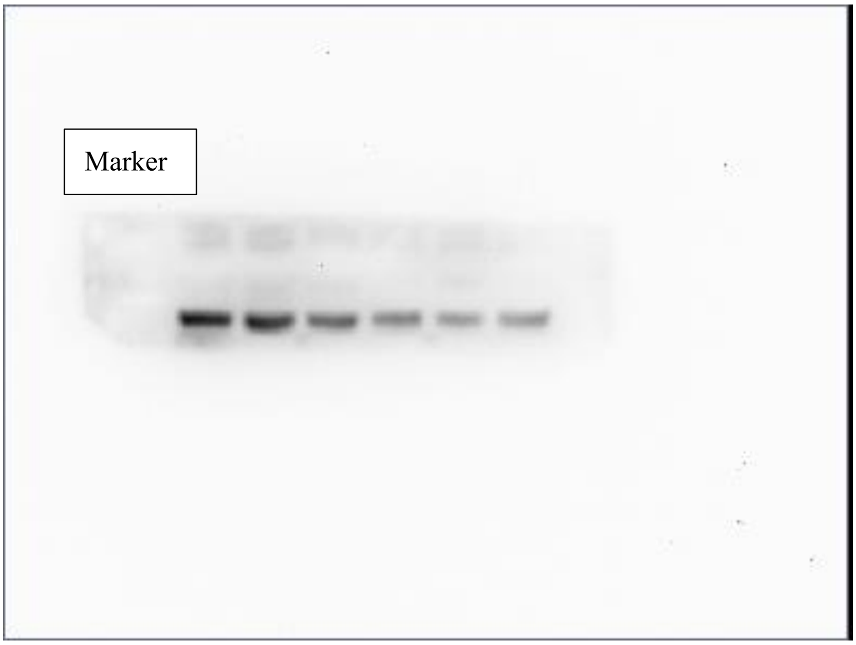

Repeat test 2

Left to right: HGC-27 (shNC + inhibitor-NC, shFAAH + inhibitor-NC, shFAAH + inhibitor-1275), MKN-1 (pcDNA + mimic-NC, pcFAAH + mimic-NC, pcFAAH + mimic-1275)

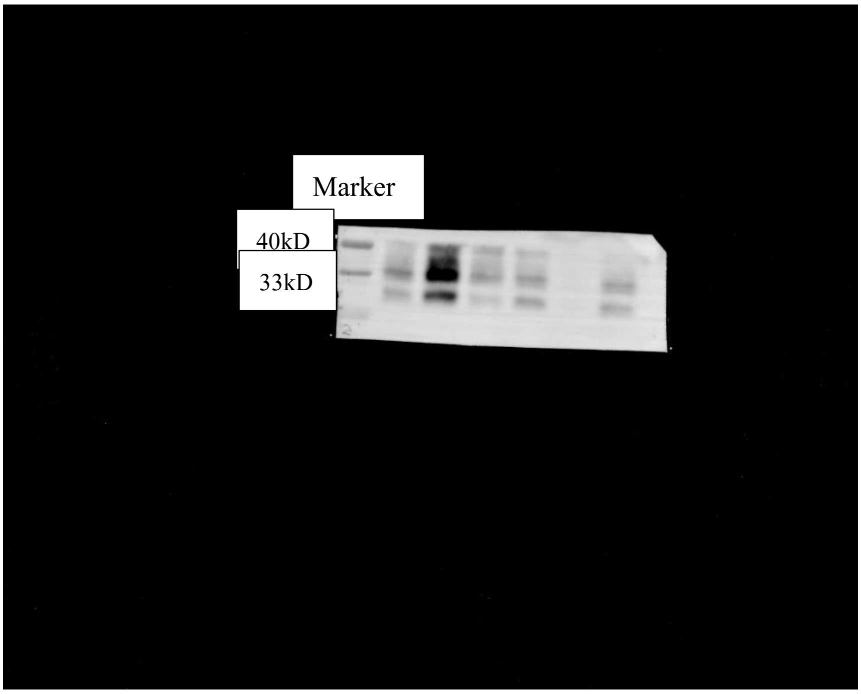

Repeat test 3

Left to right: HGC-27 (shNC + inhibitor-NC, shFAAH + inhibitor-NC, shFAAH + inhibitor-1275), MKN-1 (pcDNA + mimic-NC, pcFAAH + mimic-NC, pcFAAH + mimic-1275)

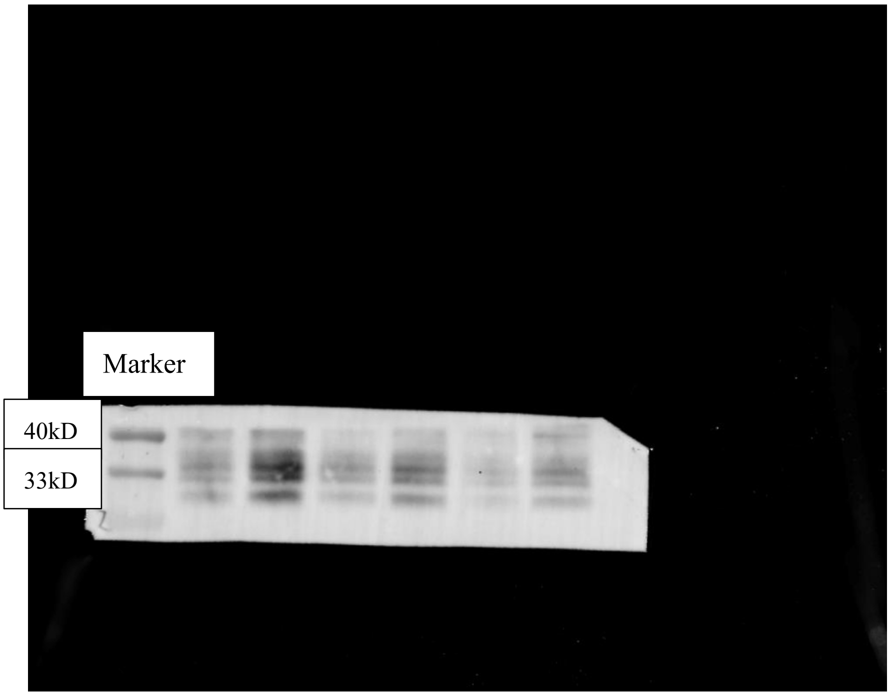

NAPE-PLD (46kD)

Repeat test 1

Left to right: HGC-27 (shNC + inhibitor-NC, shFAAH + inhibitor-NC, shFAAH + inhibitor-1275), MKN-1 (pcDNA + mimic-NC, pcFAAH + mimic-NC, pcFAAH + mimic-1275)

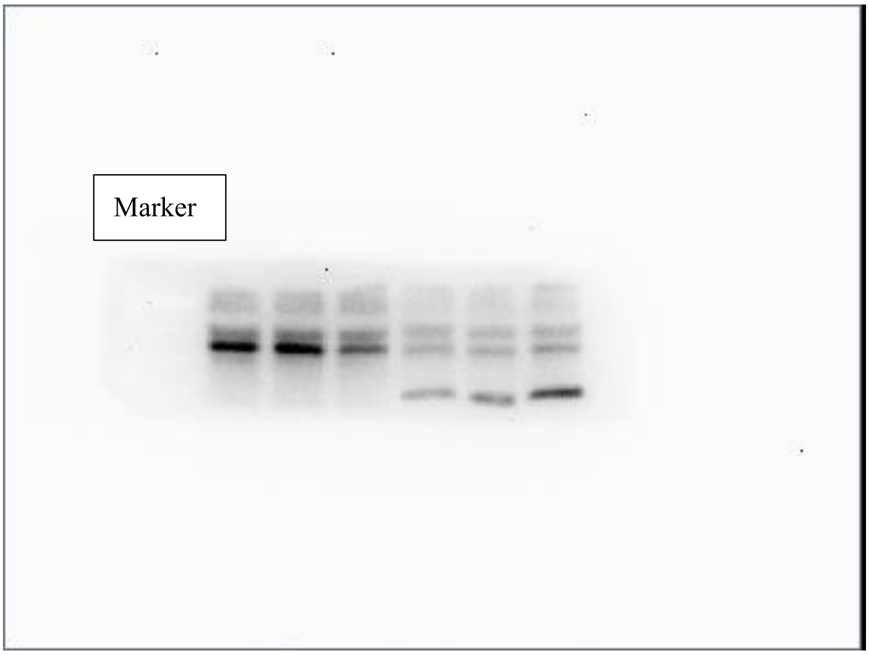

Repeat test 2

Left to right: HGC-27 (shNC + inhibitor-NC, shFAAH + inhibitor-NC, shFAAH + inhibitor-1275), MKN-1 (pcDNA + mimic-NC, pcFAAH + mimic-NC, pcFAAH + mimic-1275)

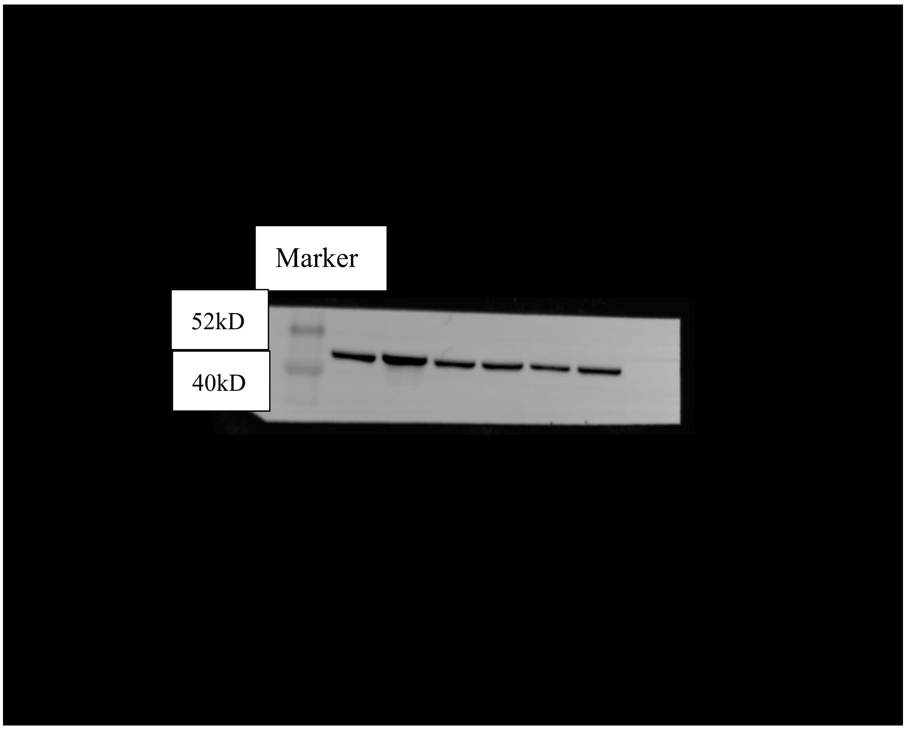

Repeat test 3

Left to right: HGC-27 (shNC + inhibitor-NC, shFAAH + inhibitor-NC, shFAAH + inhibitor-1275), MKN-1 (pcDNA + mimic-NC, pcFAAH + mimic-NC, pcFAAH + mimic-1275)

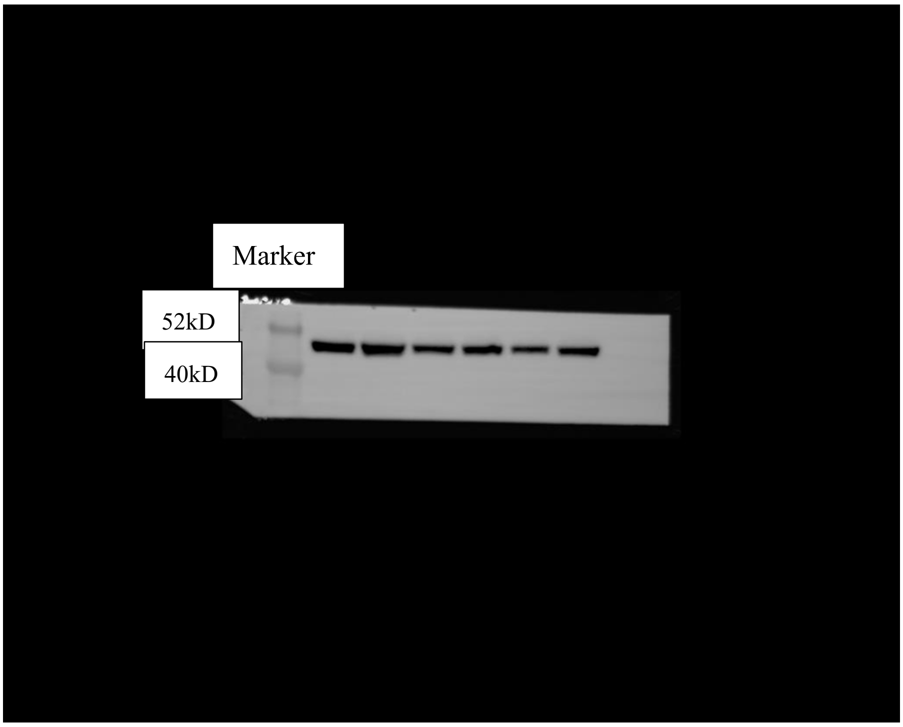

β-actin (42kD)

Repeat test 1

Left to right: HGC-27 (shNC + inhibitor-NC, shFAAH + inhibitor-NC, shFAAH + inhibitor-1275), MKN-1 (pcDNA + mimic-NC, pcFAAH + mimic-NC, pcFAAH + mimic-1275)

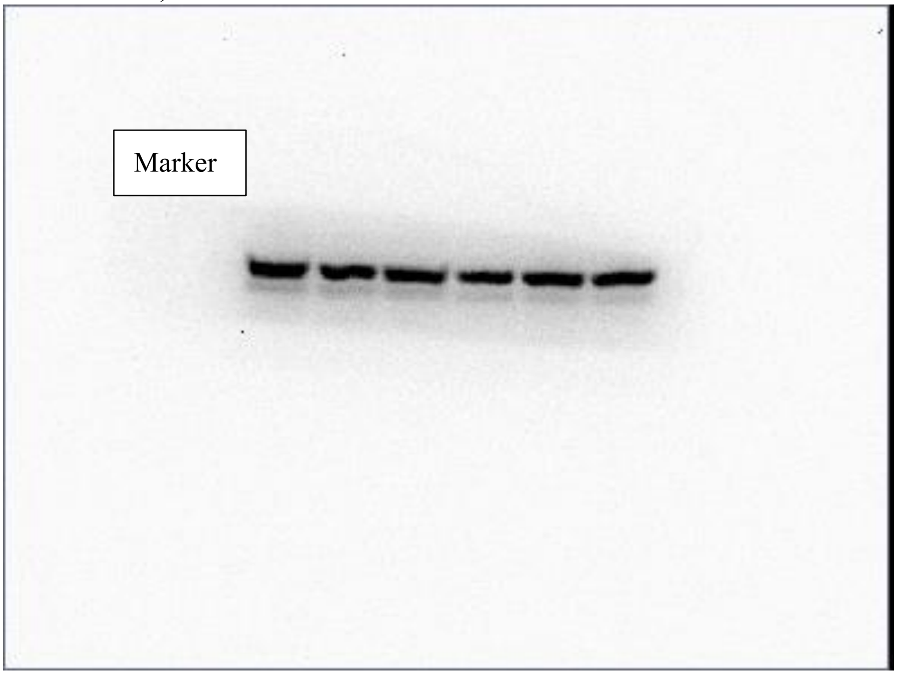

Repeat test 2

Left to right: HGC-27 (shNC + inhibitor-NC, shFAAH + inhibitor-NC, shFAAH + inhibitor-1275), MKN-1 (pcDNA + mimic-NC, pcFAAH + mimic-NC, pcFAAH + mimic-1275)

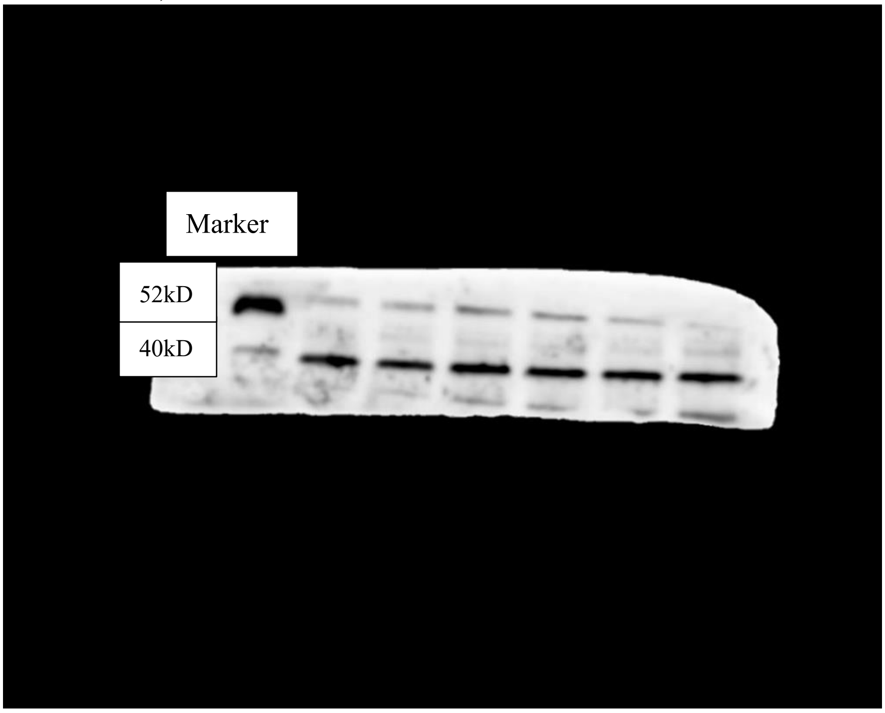

Repeat test 3

Left to right: HGC-27 (shNC + inhibitor-NC, shFAAH + inhibitor-NC, shFAAH + inhibitor-1275), MKN-1 (pcDNA + mimic-NC, pcFAAH + mimic-NC, pcFAAH + mimic-1275)

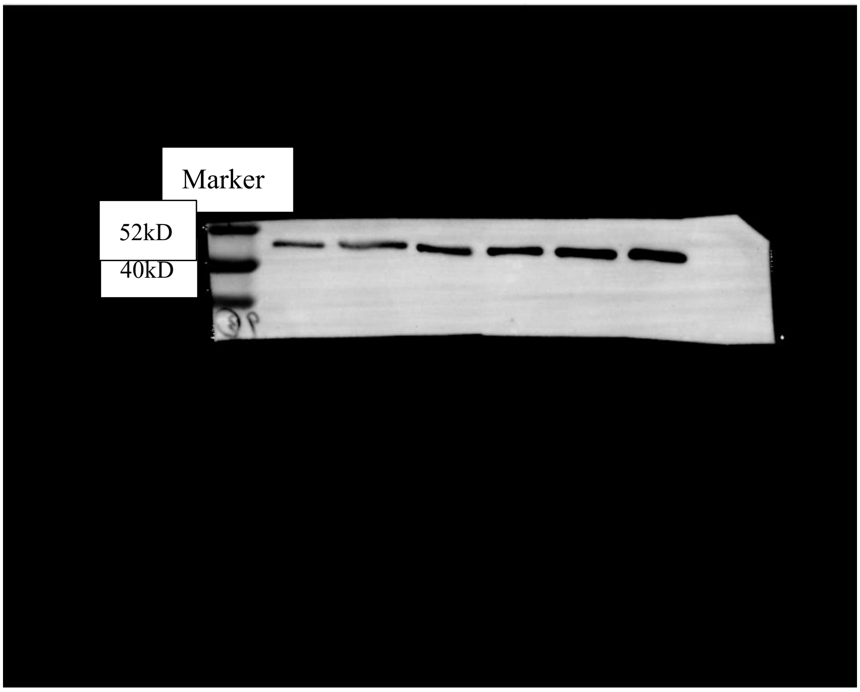

Supplement: Supplementary file 8 — Supplementary material for original western blots [file 41419_2023_5584_MOESM8_ESM.pdf]
